# Management Practices Associated With Prevalence of Lameness in Lambs in 2012–2013 in 1,271 English Sheep Flocks

**DOI:** 10.3389/fvets.2020.519601

**Published:** 2020-10-27

**Authors:** Katharine Eleanor Lewis, Laura Elizabeth Green

**Affiliations:** ^1^School of Life Sciences, Gibbet Hill, Warwick University, Coventry, United Kingdom; ^2^Institute of Microbiology and Infection, College of Life and Environmental Sciences, University of Birmingham, Birmingham, United Kingdom

**Keywords:** sheep, lamb, lameness, footrot, treatment, antibiotics, multinomial model, latent class analysis

## Abstract

The evidence base for management practices associated with low prevalence of lameness in ewes is robust. Current best practice is prompt treatment of even mildly lame sheep with parenteral and topical antibiotics with no routine or therapeutic foot trimming and avoiding routine footbathing. To date, comparatively little is known about management of lameness in lambs. Data came from a questionnaire completed by 1,271 English sheep farmers in 2013. Latent class (LC) analyses were used to investigate associations between treatment of footrot and geometric mean flock prevalence of lameness (GMPL) in lambs and ewes, with multinomial models used to investigate effects of flock management with treatment. Different flock typologies were identified for ewes and lambs. In both ewe and lamb models, there was an LC (1) with GMPL <2%, where infectious causes of lameness were rare, and farmers rarely treated lame animals. There was a second LC in ewes only (GMPL 3.2%) where infectious causes of lameness were present but farmers followed “best practice” and apparently controlled lameness. In other typologies, farmers did not use best practice and had higher GMPL than LC1 (3.9–4.2% and 2.8–3.5%, respectively). In the multinomial model, farmers were more likely to use parenteral antibiotics to treat lambs when more than 2–5% of lambs were lame compared with ≤2%. Once >10% of lambs were lame, while farmers were likely to use parenteral antibiotics, only sheep with locomotion score >2 were considered lame, leaving lame sheep untreated, potentially allowing spread of footrot. These farmers also used poor practices of routine foot trimming and footbathing, delayed culling, and poor biosecurity. We conclude there are no managements beneficial to manage lameness in lambs different from those for ewes; however, currently lameness in lambs is not treated using “best practice.” In flocks with <2% prevalence of all lameness, where infectious causes of lameness were rare, farmers rarely treated lame animals but also did not practice poor managements of routine foot trimming or footbathing. If more farmers adopted “best practice” in ewes and lambs, the prevalence of lameness in lambs could be reduced to <2%, antibiotic use would be reduced, and sheep welfare would be improved.

## Introduction

Lameness in sheep is a serious health and welfare issue. Footrot occurs in sheep-producing countries across the world (1–3) and causes the majority of lameness in the United Kingdom (4, 5). Footrot is an infectious bacterial disease caused by *Dichelobacter nodosus* (6–8) with two clinical presentations: interdigital dermatitis (ID), where there is inflammation of the interdigital skin, and severe footrot (SFR), where the hoof horn separates from the underlying tissue. The cost of lameness in the United Kingdom is estimated to be £6.35 per ewe in flocks with >10% prevalence of lameness, while it falls to £3.90 per ewe in flocks with <5% prevalence of lameness, highlighting that improved control is cost-effective despite costs of treatment (9).

Overall, control of lameness in sheep in England is improving, the global mean prevalence of lameness in ewes (LiE) fell from 10.6% in 2004 (10) to 4.9% in 2013 (5). However, the prevalence of lameness is still higher than the target of <2% of the national flock lame by 2021, set by the Farm Animal and Welfare Council (11), an advisory body to UK Government. A prevalence of LiE of <2% is achievable when sheep with footrot are treated within 0–3 days of becoming lame with parenteral and topical antibiotics without foot trimming (12, 13); this is current “best practice” (14).

In observational studies, recognition of mildly lame sheep (5, 10), treatment of all lame sheep within 3 days of onset of lameness, inspection and isolation of brought-in sheep, vaccination against footrot, selecting replacement breeding ewes from never-lame mothers (5), separating lame sheep at treatment (15, 16), culling sheep lame twice or more in a year (16), and not practicing routine foot trimming or routine footbathing (5, 10, 16) contribute to low flock prevalence of LiE.

Like ewes, lambs are susceptible to both ID and SFR, and outbreaks of ID are common in spring in flocks of ewes with lambs (13, 15). The prevalence of LiE and lambs is positively correlated within a flock (5, 17), and there are associations between prevalence of foot lesions in ewes and foot lesions in lambs (18). To our knowledge, the only management practices associated to date with a lower prevalence of lameness in lambs (LiL) include “always” catching and treating lame ewes with parenteral antibiotics compared with not always doing so (18).

The aim of the current study was to identify management practices associated with the flock prevalence of LiL. Data from a questionnaire sent to farmers in 2013 (5) was used, and two farm-level hypotheses were investigated: (1) that management practices associated with prevalence of LiE are associated with prevalence of LiL and (2) that there are specific managements associated with the prevalence of LiL that are not associated with the prevalence of LiE. The ultimate goal was to provide information for the sheep industry that could be used to reduce prevalence of LiL and so contribute to the FAWC 2011 target of <2% lameness in sheep flocks by 2021.

## Materials and Methods

Ethical approval was obtained from the University of Warwick's Biomedical and Scientific Research Ethics Committee (BSREC), reference number BSREC 159-01-12; approved December 7, 2011. The farmers were all informed of the purpose of the study and their right to withdraw at any point; responding to the questionnaire was indication that they consented to participate. All participants owned or rented a farm, indicating they were older than 16 years and could consent to participation in the study.

### Questionnaire Design and Administration

Data came from a 14-page postal questionnaire (5) that requested information on the mean annual flock prevalence of LiL and ewes and management practices used to control lameness for the period May 2012 to April 2013. The questionnaire (Supplementary Figure 1) was sent to a random sample (stratified by county and size) of 4,000 lowland sheep farmers in England reported to have >200 ewes. Up to two reminder letters were sent to non-respondents with a second copy of the questionnaire with the second reminder. Double data entry was carried out by an outside agency (Wyman Dillon Ltd., Bristol), and then data were cleaned and stored in Microsoft Excel as described by Winter et al. (5). A total of 1,348 questionnaires were returned after two reminders; 1,271 (31.8%) responses were usable. The number and percentage of farmers using different management strategies for lameness are in the Supplementary Material from Winter et al. (5). Responses were excluded from analysis for this study if data on annual mean period prevalence of LiL or LiE or ewe flock size were missing.

### Descriptive Statistics

Data analysis was carried out in R Studio v3.4.1 (19). Flocks were categorized by prevalence of LiL and LiE into ≤2%, >2–5%, >5–10%, and >10%. These categories were based on the FAWC targets of <2% of the national flock lame by 2021 and ≤5% of the national flock lame by 2016 (11); >5–10% from the global mean prevalence of lameness in 2013 and 2004, respectively (5, 10), and >10%, the flock prevalence of LIE deemed unacceptable by farmers (20). The number and percentage of flocks managed using each practice were calculated for each category of LiL (Supplementary Table 1). The relationship between the geometric mean LiE and LiL was investigated using paired Wilcoxon tests and Spearman correlation coefficient tests. The questionnaire included a photograph and descriptions of ID, SFR, contagious ovine dermatitis (CODD), and shelly hoof. Farmers were asked what they would name each lesion and estimate its prevalence in ewes in their flock. For the analysis, the prevalence of each lesion, including correct and incorrect naming of the lesion, was used as in Winter et al. (5) and Reeves et al. (21) because farmers recognize lesions, but do not always name them correctly (4). The prevalence of lesions in lambs was not available.

### Latent Class Analysis of Methods Used by Farmers to Treat Footrot in Ewes and Lambs

Two separate latent class analyses (LCAs), one for lambs and one for ewes, were used to determine typologies of farmers by treatment of footrot using the “*poLCA*” R package (22), which identifies latent classes (LCs) using the expectation–maximization algorithm (22). Models ranging from two to seven classes were obtained by running 500 repetitions of each model using 20,000 iterations of the expectation–maximization algorithm to increase confidence that the final solution for each model had converged on a global maximum solution. Goodness-of-fit statistics [Akaike Information Criterion (AIC), Bayesian Information Criterion (BIC) and goodness-of-fit test] were calculated, with the BIC used as the primary selection criteria (23), to determine the optimum number of classes. The posterior probability for each farmer being in a class and the conditional probability that farmers in a class were practicing a management were calculated for the models with the optimum number of classes for lambs and ewes.

For the optimal models for both treatment of ewes and treatment of lambs, the geometric mean LiE and LiL and associated 95% confidence intervals (CIs) were calculated within LCs. Pairwise Wilcoxon tests with the Benjamini–Hochberg correction, which accounts for multiple comparisons by controlling the false discovery rate (24), were used to investigate differences in prevalence of lameness and foot lesions by LC for lambs and ewes using the *ggpubr* package (25). A Benjamini–Hochberg *p* ≤ 0.05 was used as the significance threshold for a difference between LCs.

### Structure of Data and Associations Between Variables

Associations between two explanatory variables were investigated using Pearson χ^2^-test for associations between two categorical variables, with Cramer *V* statistic to indicate the strength of the association calculated using the *lsr* R package (26), Kruskal–Wallis tests for a categorical and continuous variable (27), and Pearson correlation coefficient tests for two continuous variables (28). When a question asked about sheep and did not specify lambs or ewes, it was assumed to relate to management of both groups.

### Multinomial Modeling of Associations With Management Practices and Prevalence of Lameness in Lambs and Ewes

Three models were created; these were (1) LiL with flock management practices and options for treatment of footrot in lambs as explanatory variables (Model 1), (2) LiL with flock management practices and options for treatment of footrot in ewes as explanatory variables (Model 2), and (3) LiE with flock management practices and treatment of ewes as explanatory variables (Model 3).

The models took the form:

$$\begin{array}{l} \text{logit }\left(\pi_{1\text{k}/\text{pi}0\text{k}}\right)=\;\beta_{0\text{k}}+\;\sum \beta_{0}\text{x}+\;{\text{e}}_{\text{k}}\\ \text{logit }\left(\pi_{2\text{k}/\text{pi}0\text{k}}\right)=\;\beta_{1\text{k}}+\;\sum \beta_{1}\text{x}+\;{\text{e}}_{\text{k}}\\ \text{logit }\left(\pi_{3\text{k}/\text{pi}0\text{k}}\right)=\;\beta_{2\text{k}}+\;\sum \beta_{2}\text{x}+\;{\text{e}}_{\text{k}} \end{array}$$

where the baseline is ≤2% lameness and *logit*(π_1k/pi0k_) = the probability of having >2–5% lameness, *logit*(π_2k/pi0k_)= the probability of >5–10% lameness, logit(π_3k/pi0k_) = the probability of having >10% lameness. β_0_*x*, β_1_*x*, and β_2_*x* are a series of coefficients for explanatory variables for each category of prevalence of lameness, and *e*_*k*_ is the residual variance. The “multinom” function from the “nnet” package (29) was used to fit multinomial log-linear models via neural networks.

The questions on management of lameness from the questionnaire were grouped into 10 categories: recognizing and catching lame sheep, treatment of footrot in lambs, treatment of footrot in ewes, routine trimming of sheep, footbathing, culling, and replacing ewes, vaccination, whole flock antibiotic treatment, farm biosecurity, and farm and farmer characteristics. The 10 categories listed were used to build submodels in order to investigate potentially related variables. In each submodel, the univariable associations between the prevalence of lameness and each explanatory variable were assessed. Submodels were then built using a manual forward stepwise process (30), with the variable with *p* ≤ 0.05 and the lowest AIC score used to select the variables added to the multivariable model at each step. Interactions between variables were tested by fitting the same model with an interaction term, these would have been included if they improved the AIC score and were biologically plausible. A final model was built from the 10 submodels using a manual forward stepwise approach. Finally, all variables not in the final model were retested to check for residual confounding (31). Model fit was assessed using the Hosmer–Lemeshow test for multinomial models using the “*generalhoslem*” package (32).

## Results

### The Relationships Between Prevalence of Lameness in Ewes and Lambs

The geometric mean prevalence (GMP) of LiL and LiE were 2.4% (95% CI = 2.1–2.6) and 3.4% (95% CI = 3.2–3.7), respectively; the LiL and LiE within a flock were significantly positively correlated (Spearman ρ = 0.62, *p* < 0.01), and LiL was significantly lower than LiE (paired Wilcoxon test *p* < 0.01). The geometric mean LiL was also lower than LiE in each of the ≤2%, >2–5%, and >5–10% lameness categories but higher in the >10% lameness category (Table 1), indicating that the distribution of prevalence of LiL was more dispersed than LiE. Numbers and percentages of farmers performing each management practice in each pre-established category of prevalence of lameness are found in Supplementary Table 1.

**Table 1:** Geometric mean period flock prevalence of lameness and 95% CI in lambs and ewes by pre-established category of prevalence of lameness from 1,271 flocks in England.

**Pre-established category of lameness (%)**	**Lambs**		**Ewes**	
	**GM prevalence of lameness and 95% CI (%)**	**No. (%) of flocks**	**GM prevalence of lameness and 95% CI (%)**	**No. (%) of flocks**
≤2	0.7 (0.6–0.9)	553 (43.5)	1.1 (1.0–1.3)	413 (32.5)
>2–5	4.1 (4.0–4.2)	456 (35.9)	4.1 (4.0–4.2)	544 (42.8)
>5–10	8.7 (8.4–8.9)	165 (13.0)	8.6 (8.4–8.8)	222 (17.5)
>10	19.4 (18.0–20.9)	97 (7.6)	18.3 (17.2–19.4)	92 (7.2)

*GM, geometric mean; 95% CI, 95% confidence interval*.

### Latent Class Analyses of Treatments for Footrot in Ewes and Lambs

The LCAs for lambs and ewes were both optimal with four typologies of treatment for footrot, although the class attributes were different for lambs and ewes. Fit statistics for all tested models (two to seven classes) are shown in Supplementary Tables 10a,b; standard errors for class conditional probabilities are shown in Supplementary Tables 11a,b.

For typologies of treatment of lambs, the geometric mean LiL ranged from 1.0 to 3.5% (Table 2). Flocks in LC1 had significantly lower LiL (BH-adjusted Wilcoxon *p* ≤ 0.05) than flocks in LC2, LC3, and LC4 (Table 2). The prevalence of LiE did not differ significantly across typologies for treatment of lambs.

**Table 2:** Latent class models for treatment of lambs and ewes.

**Latent class**	**No. of flocks**	**GM prevalence of lameness in lambs (95% CI)**	**BH-adjusted** ***P***			**GM prevalence of lameness in ewes (95% CI)**	**BH-adjusted** ***P***		
			**LC2**	**LC3**	**LC4**		**LC2**	**LC3**	**LC4**
**Treatment of lambs latent class model**									
LC1	117	1.0 (0.6–1.7)^a^	<0.001	<0.001	<0.001	2.8 (2.0–3.9)^a^	0.70	0.70	0.70
LC2	214	2.8 (2.2–3.5)^b^		0.60	0.50	3.7 (3.2–4.3)^a^		0.90	0.90
LC3	257	3.1 (2.3–3.7)^b^			0.74	4.0 (3.6–4.4)^a^			0.90
LC4	235	3.5 (3.1–4.1)^b^			–	3.9 (3.5–4.3)^a^			–
**Treatment of ewes latent class model**									
LC1	86	1.1 (0.6–2.1)^a^	0.15	0.04	<0.01	1.8 (1.0–3.1)^a^	0.62	0.01	0.01
LC2	134	2.4 (1.8–3.2)^ab^		0.45	0.15	3.2 (2.9–3.7)^a^		0.02	0.01
LC3	198	2.5 (1.9–3.3)^b^			0.45	3.9 (3.5–4.4)^b^			0.78
LC4	490	3.0 (2.6–3.4)^b^			–	4.2 (3.9–4.5)^b^			–

*Number of flocks, geometric mean period prevalence of lameness and 95% confidence intervals for treatment of lambs (823 flocks) and treatment of ewes (908 flocks) with footrot in England, 2012–2013*.

*GM, geometric mean; 95% CI, 95% confidence interval (lower, upper); BH, Benjamini–Hochberg. Where superscripts (a, b) differ across rows, prevalence of lesion or lameness differs (BH-adjusted Wilcoxon p ≤ 0.05) between latent classes. BH-adjusted Wilcoxon p-values are shown for all pairwise comparisons of prevalence of lameness between latent classes*.

For typologies of treatment for ewes, the geometric mean LiE ranged from 1.8 to 4.2% (Table 2). In contrast to treatment of lambs, LC1 and LC2 had significantly (BH-adjusted Wilcoxon *p* ≤ 0.05) lower LiE than both LC3 and LC4, with no significant difference LiE between LC1 and LC2 or LC3 and LC4 (Table 2).

#### Description of Typologies for Use of Treatment of Footrot in Ewes and Lambs

**Figure 1:**
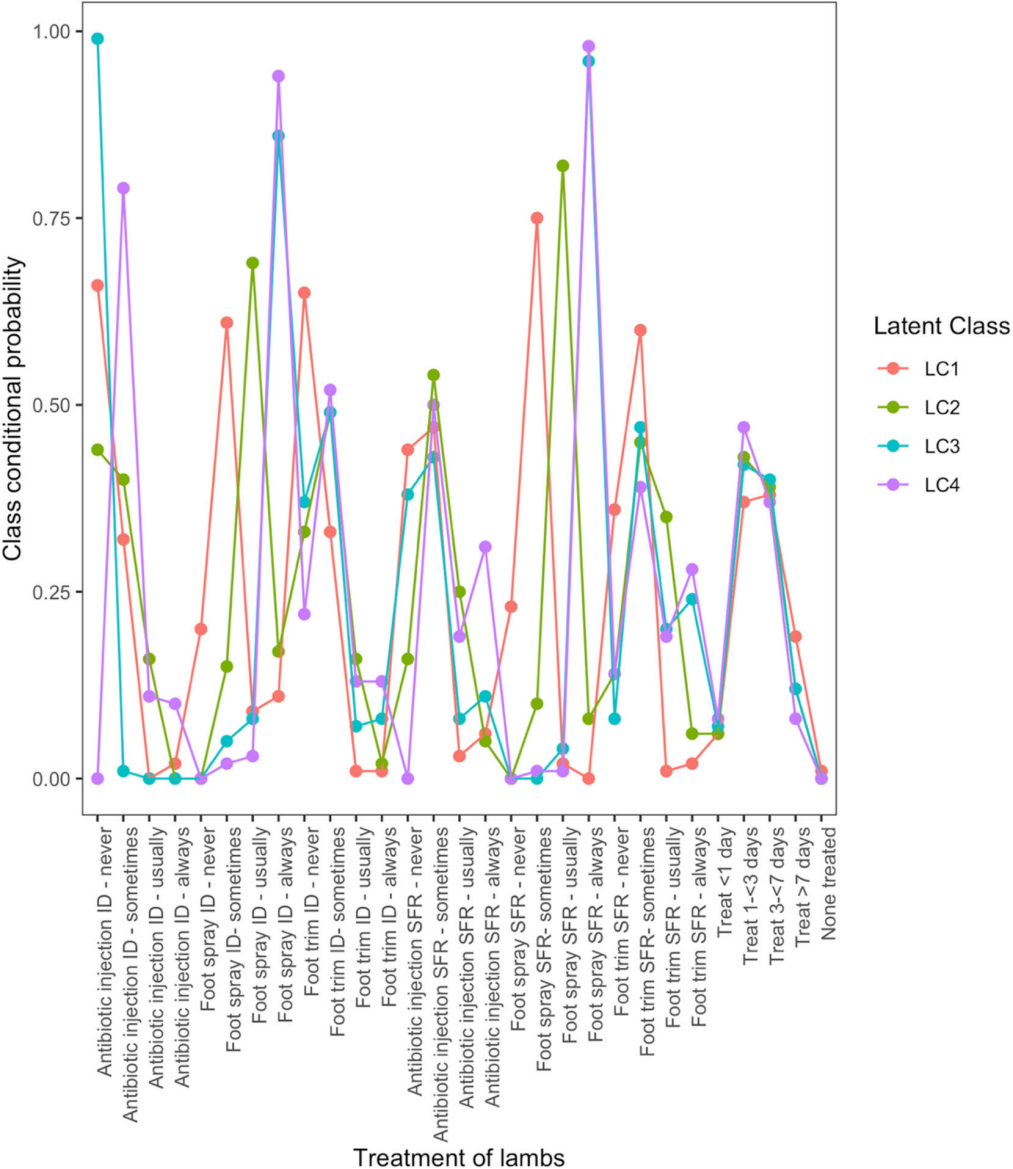
Conditional probabilities that a farmer used a type and frequency of treatment on lambs with interdigital dermatitis or severe footrot from a four-class latent class model, for 823 flocks of sheep in England, 2012–2013.

There were four typologies for treatment of footrot in lambs. LC1 (Figure 1) was characterized by little treatment of lambs with farmers “sometimes” using antibiotic injection, foot spray, or foot trimming. LC2 was similar to LC1, but with more use of treatments, 86 and 96% of farmers “always” used topical spray to treat ID and SFR, respectively, but 99 and 33% of farmers “never” used antibiotic injection to treat lambs with ID and SFR, respectively. Farmers in LC3 treated lambs “always” with topical spray for ID (86%) and SFR (96%) but were reluctant to use antibiotic injection to treat lambs (38% of farmers never using to treat lambs with SFR). Farmers in LC4 were most likely to always treat lambs, with 31% “always” using antibiotic injection to treat lambs with SFR and the majority “always” using foot spray to treat lambs with ID (94%) or SFR (98%).

**Figure 2:**
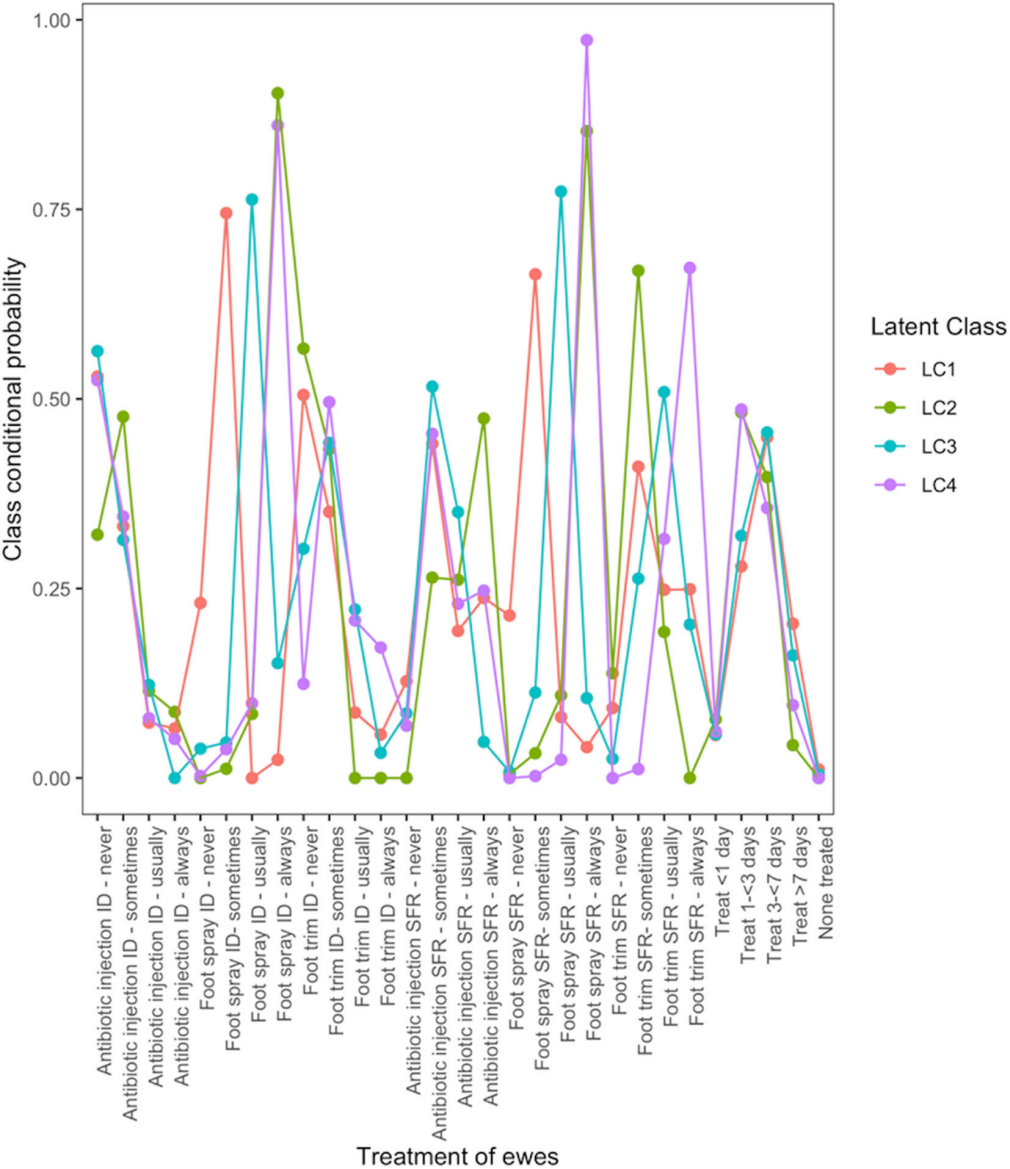
Conditional probabilities that a farmer used a type and frequency of treatment on ewes with interdigital dermatitis or severe footrot from a four-class latent class model for 908 flocks of sheep in England, 2012–2013.

There were four typologies of treatment for lame ewes (Figure 2). Farmers in LC1 used treatment for ID and SFR “sometimes.” Farmers in LC2 followed “best practice” most with 90 and 85% of farmers using foot spray to treat ID and SFR but only 47% using antibiotic injection to treat SFR. Farmers in LC3 did not use parenteral antibiotic but “usually” treated ID and SFR with topical spray (76 and 77% of farmers, respectively), whereas farmers in LC4 were most likely to manage footrot traditionally, by “always” using foot spray to treat ID and SFR (86 and 97%, respectively), and also foot trimming to treat SFR (67%).

#### Associations Between Typology for Use of Treatment and Prevalence of Lameness and Foot Lesions

LC analyses identified one typology (LC1) both in lambs (Figure 1) and ewes (Figure 2), where farmers had little lameness and used treatment infrequently, suggesting a low prevalence of lameness in some flocks that was stable with only using treatments for ID and SFR “sometimes.” In these flocks, some farmers reported zero lameness (Table 3), and ewes in flocks in LC1 were less likely to have ID (GMP = 1.06, 95% CI = 0.48–2.34) than LC2 (GMP = 4.01%, 95% CI = 3.02–5.32) (Table 4). For treatment of ewes but not lambs, there was a typology where farmers followed “best practice” (LC2, Figure 2). Given that there was no significant difference in prevalence of LiE in LC1 and LC2, these farmers were actively controlling lameness successfully. Ewe flocks in LC3 and LC4 had significantly higher prevalence of LiE (LC3: GMP = 3.9%, 95% CI = 3.5–4.4), LC4: GMP = 4.2%, 95% CI = 3.9–4.5) and did not follow “best practice” guidelines. Farmers in LC3 “usually” treated SFR with foot spray and antibiotic injection but were less likely to treat within 3 days (Figure 2), while farmers in LC4 treated SFR with detrimental managements including “always” using foot spray with foot trimming (Figure 2). These flocks also had significantly higher prevalence of CODD lesions than flocks in LC2 (Tables 3, 4). Typologies for treatment of lambs in LC2 to LC4 were less distinct, and the prevalence of LiL was not significantly different.

**Table 3:** Percentage of flocks with lame ewes/lambs and lesions as reported by farmers, by latent class for models of treatment of lambs (823 flocks) and ewes (908 flocks).

**Latent class**	Farmer^*^ reported presence of lameness/lesions	**No. of flocks (percentage)**					
		**Lame ewes**	**Lame lambs**	**Interdigital dermatitis**	**Severe footrot**	**Contagious ovine digital dermatitis**	**Shelly hoof**
**Treatment of lambs latent class model**							
LC1	Absent	3 (2.6)	10 (8.5)	14 (12.0)	20 (17.1)	56 (47.9)	32 (27.4)
	Present	114 (97.4)	107 (91.5)	95 (81.2)	88 (75.2)	53 (45.3)	43 (36.8)
	Not reported^**^	0 (0.0)	0 (0.0)	8 (6.8)	9 (7.7)	8 (6.8)	42 (35.9)
LC2	Absent	1 (0.5)	4 (1.9)	11 (5.1)	21 (9.8)	75 (35.0)	43 (20.1)
	Present	213 (99.5)	210 (98.1)	183 (85.5)	176 (82.2)	119 (55.6)	102 (47.7)
	Not reported^**^	0 (0.0)	0 (0.0)	20 (9.3)	17 (7.9)	20 (9.3)	69 (32.2)
LC3	Absent	0 (0.0)	3 (1.2)	6 (2.3)	23 (8.9)	89 (34.6)	51 (19.8)
	Present	257 (100.0)	254 (98.8)	232 (90.3)	218 (84.8)	149 (58.0)	126 (49.0)
	Not reported^**^	0 (0.0)	0 (0.0)	19 (7.4)	16 (6.2)	19 (7.4)	80 (31.1)
LC4	Absent	0 (0.0)	1 (0.4)	5 (2.1)	27 (11.5)	97 (41.3)	46 (19.6)
	Present	235 (100.0)	234 (99.6)	216 (91.9)	196 (83.4)	120 (51.1)	101 (43.0)
	Not reported^**^	0 (0.0)	0 (0.0)	14 (6.0)	12 (5.1)	18 (7.7)	88 (37.4)
**Treatment of ewes latent class model**							
LC1	Absent	5 (5.8)	7 (8.2)	10 (11.6)	13 (15.1)	35 (40.7)	22 (25.6)
	Present	81 (94.2)	79 (91.9)	72 (83.7)	66 (76.7)	45 (52.3)	41 (47.7)
	Not reported^**^	0 (0.0)	0 (0.0)	4 (4.7)	7 (8.1)	6 (7.0)	23 (26.7)
LC2	Absent	0 (0.0)	3 (2.2)	2 (1.5)	13 (9.7)	68 (50.7)	31 (23.1)
	Present	134 (100.0)	131 (97.8)	126 (94.0)	116 (86.6)	57 (42.5)	49 (36.6)
	Not reported^**^	0 (0.0)	0 (0.0)	6 (4.5)	5 (3.7)	9 (6.7)	54 (40.3)
LC3	Absent	0 (0.0)	5 (2.5)	14 (7.1)	20 (10.1)	73 (36.9)	35 (17.7)
	Present	198 (100.0)	193 (97.5)	164 (82.8)	163 (82.3)	103 (52.0)	85 (42.9)
	Not reported^**^	0 (0.0)	0 (0.0)	20 (10.1)	15 (7.6)	22 (11.1)	78 (39.4)
LC4	Absent	0 (0.0)	7 (1.4)	14 (2.9)	54 (11.0)	186 (38.0)	111 (22.7)
	Present	490 (100.0)	483 (98.6)	444 (90.6)	404 (82.3)	272 (55.5)	223 (45.5)
	Not reported^**^	0 (0.0)	0 (0.0)	32 (6.5)	32 (6.5)	32 (6.5)	156 (31.8)

* *Farmers reported whether they had observed any lame ewes or lame lambs or presence of foot lesions in ewes)*.

** *Farmers did not reply to the question*.

**Table 4:** Geometric mean, 95% confidence intervals, and Benjamini–Hochberg–adjusted Wilcoxon *p*-values for pairwise comparisons of prevalence of foot lesions in ewes by latent class for treatment of lambs (823 flocks) and treatment of ewes (908 flocks).

	**Treatment of lambs latent class model**				**Treatment of ewes latent class model**			
**Latent class**	**GM prevalence of foot lesions in ewes**	**BH-adjusted** ***p***			**GM prevalence of foot lesions in ewes**	**BH-adjusted** ***p***		
		**LC2**	**LC3**	**LC4**		**LC2**	**LC3**	**LC4**
**Interdigital dermatitis**								
LC1	1.09 (0.54–2.18)^a^	0.18	0.03	0.18	1.06 (0.48–2.34)^a^	0.03	0.22	0.01
LC2	2.65 (1.82–3.85)^ab^	–	0.31	0.78	4.01 (3.02–5.32)^bc^	–	0.18	0.79
LC3	3.92 (3.06–5.02)^b^		–	0.20	1.84 (1.18–2.87)^ab^		–	0.04
LC4	3.52 (2.77–4.47)^ab^			–	3.54 (2.93–4.27)^c^			–
**Severe footrot**								
LC1	0.47 (0.21–1.03)^a^	0.49	0.49	0.64	0.51 (0.21–1.24)^a^	0.28	0.04	0.09
LC2	1.09 (0.68–1.73)^a^	–	0.82	0.64	0.98 (0.56–1.72)^ab^	–	0.09	0.34
LC3	1.19 (0.80–1.79)^a^		–	0.68	1.15 (0.71–1.89)^b^		–	0.18
LC4	0.89 (0.56–1.42)^a^			–	0.95 (0.69–1.30)^ab^			–
**Contagious ovine digital dermatitis**								
LC1	0.01 (0.00–0.03)^a^	0.10	0.04	0.32	0.02 (0.01–0.07)^ab^	0.61	0.17	0.12
LC2	0.05 (0.02–0.09)^ab^	–	0.32	0.32	0.01 (0.00–0.02)^b^	–	0.04	0.02
LC3	0.06 (0.03–0.11)^b^		–	0.10	0.04 (0.02–0.08)^a^		–	0.94
LC4	0.03 (0.01–0.05)^ab^			–	0.04 (0.03–0.06)^a^			–
**Shelly hoof**								
LC1	0.03 (0.01–0.10)^a^	0.30	0.18	0.30	0.06 (0.02–0.18)^a^	0.70	0.26	0.26
LC2	0.11 (0.05–0.22)^a^	–	0.30	0.81	0.05 (0.02–0.14)^a^	–	0.26	0.35
LC3	0.13 (0.07–0.27)^a^		–	0.33	0.11 (0.05–0.27)^a^		–	0.60
LC4	0.10 (0.05–0.21)^a^			–	0.08 (0.05–0.14)^a^			–

*GM, geometric mean; 95% CI, 95% confidence interval (lower, upper); BH, Benjamini–Hochberg. Where superscripts (a, b, c) differ across rows, prevalence of lesion or lameness differs (BH-adjusted Wilcoxon p ≤ 0.05) between latent classes*.

### Multivariable Models of Associations Between Management of Lameness and Prevalence of Lameness in Lambs and Ewes

Univariable models and submodels for each group of explanatory variables are in Supplementary Tables 2, 3. Flock managements and treatment choices were similar for ewes and lambs within flocks (Supplementary Table 4), and so separate models were developed to investigate flock managements and treatments of lambs (Model 1, Table 5) and flock management and treatment of ewes (Model 2, Table 5, Supplementary Table 7) associated with LiL and associations between management and treatment of ewes and LiE (Model 3, Table 5, Supplementary Table 8).

**Table 5:** Comparison of the three multivariable multinomial models of factors associated with prevalence of lameness in lambs and ewes in 842, 973, and 964 flocks of sheep (respectively) in England, 2012–2013.

**Predictor**	**Model 1: management of lameness in lambs/prevalence of lameness in lambs**			**Model 2: management of lameness in ewes/prevalence of lameness in lambs**			**Model 3: management of lameness in ewes/prevalence of lameness in ewes**		
**Category of lameness**	**>2–5%**	**>5–10%**	**>10%**	**>2–5%**	**>5–10%**	**>10%**	**>2–5%**	**>5–10%**	**>10%**
Treat lambs with severe footrot with antibiotic injection (baseline always) Usually	0.64 (0.36–1.16)	0.82 (0.34–1.96)	**0.32 (0.10–1.00)**	x	x	x	x	x	x
Sometimes	**0.48 (0.29–0.80)**	1.01 (0.49–2.21)	0.54 (0.23–1.25)	x	x	x	x	x	x
Never	**0.42 (0.24–0.73)**	0.56 (0.24–1.30)	**0.27 (0.09–0.76)**	x	x	x	x	x	x
Treat ewes with severe footrot with antibiotic injection (baseline always) Usually	x	x	x	0.71 (0.47–1.08)	**2.09 (1.15–3.81)**	0.81 (0.39–1.67)	1.29 (0.83–2.01)	1.22 (0.68–2.19)	1.18 (0.51–2.73)
Sometimes	x	x	x	0.75 (0.51–1.09)	1.23 (0.68–2.23)	0.80 (0.41–1.55)	1.08 (0.72–1.63)	0.88 (0.51–1.50)	0.94 (0.44–2.00)
Never	x	x	x	**0.47 (0.25–0.88)**	0.72 (0.26–1.98)	**0.11 (0.01–0.92)**	**0.28 (0.14–0.56)**	**0.32 (0.13–0.80)**	0.21 (0.04–1.07)
Foot trimming used to treat lambs with severe footrot (baseline always) Usually	1.07 (0.64–1.80)	0.82 (0.34–1.96)	1.16 (0.47–2.83)	x	x	x	x	x	x
Sometimes	1.10 (0.69–1.75)	1.01 (0.49–2.21)	1.30 (0.59–2.89)	x	x	x	x	x	x
Never	0.69 (0.04–1.19)	0.56 (0.24–1.30)	**0.11 (0.01–0.87)**	x	x	x	x	x	x
Footbath used to prevent interdigital dermatitis (baseline no) Yes	x	x	x	x	x	x	1.22 (0.86–1.74)	1.01 (0.64–1.59)	**0.44 (0.22–0.87)**
Footbath used to treat severe footrot (baseline no) Yes	1.19 (0.85–1.68)	1.30 (0.81–2.08)	**2.63 (1.45–4.75)**	1.28 (0.94–1.75)	**1.61 (1.04–2.49)**	**2.86 (1.68–4.85)**	**1.51 (1.05–2.18)**	**2.40 (1.52–3.79)**	**2.81 (1.51–5.22)**
Vaccination of sheep with severe footrot (baseline no) Yes	**3.32 (1.03–10.67)**	1.89 (0.39–9.18)	3.65 (0.59–22.71)	**4.46 (1.40–14.20)**	3.40 (0.80–14.48)	**7.00 (1.40–35.07)**	x	x	x
**Vaccination of ewes** (baseline no) Yes	x	x	x	x	x	x	**0.62 (0.41–0.94)**	**0.39 (0.21–0.71)**	0.65 (0.29–1.45)
Routine foot trim the flock (baseline no) Trim no bleeding	1.04 (0.53–2.02)	0.24 (0.05–1.08)	2.91 (0.80–10.57)	0.90 (0.49–1.65)	0.44 (0.15–1.32)	2.04 (0.66–6.27)	1.28 (0.68–2.42)	1.46 (0.59–3.63)	**4.11 (1.15–14.65)**
Bleeding	1.39 (0.99–1.94)	0.79 (0.50–1.27)	**4.16 (2.03–8.53)**	**1.38 (1.01–1.87)**	0.95 (0.62–1.47)	**3.25 (1.77–5.95)**	**1.71 (1.22–2.40)**	**2.42 (1.56–3.76)**	**5.53 (2.80–10.93)**
Locomotion score farmer recognized sheep as lame (baseline score 1) 2	1.30 (0.92–1.83)	1.58 (0.98–2.57)	1.78 (0.94–3.37)	1.33 (0.97–1.83)	1.38 (0.88–2.17)	**1.81 (1.02–3.22)**	x	x	x
3	1.27 (0.72–2.22)	1.18 (0.51–2.75)	**2.83 (1.17–6.82)**	1.08 (0.66–1.78)	0.93 (0.43–2.01)	1.98 (0.87–4.49)	x	x	x
4 or more	1.12 (0.15–7.77)	**7.37 (1.23–44.30)**	**10.61 (1.47–76.28)**	1.31 (0.26–6.68)	**6.14 (1.36–27.69)**	**7.14 (1.28–39.85)**	x	x	x
Number of times sheep lame before culling (baseline no culling for lameness) 1	0.60 (0.25–1.43)	0.48 (0.01–2.35)	0.00 (0.00–4.18e+109)	0.62 (0.27–1.42)	0.59 (0.16–2.14)	**0.00 (0.00–0.00)**	**0.37 (0.15–0.91)**	0.30 (0.08–1.11)	0.38 (0.05–3.19)
1– <2	0.87 (0.53–1.43)	1.41 (0.70–2.82)	0.87 (0.34–2.21)	0.75 (0.47–1.18)	1.10 (0.57–2.10)	0.81 (0.34–1.89)	1.03 (0.64–1.64)	0.71 (0.37–1.36)	0.68 (0.26–1.75)
>2	1.39 (0.94–2.05)	**2.12 (1.25–3.58)**	1.52 (0.78–3.04)	1.21 (0.85–1.73)	**1.63 (1.00–2.66)**	1.39 (0.76–2.56)	**1.82 (1.24–2.72)**	1.25 (0.75–2.09)	1.65 (0.83–3.30)
Persistently lame	**2.10 (1.04–4.21)**	1.24 (0.41–3.74)	1.40 (0.40–4.84)	**2.22 (1.14–4.33)**	1.31 (0.45–3.85)	1.80 (0.58–5.58)	**2.29 (1.06–4.95)**	1.45 (0.55–3.82)	1.70 (0.46–6.36)
Isolation of new sheep on arrival (baseline did not isolate) Sometimes	0.59 (0.29–1.20)	0.46 (0.16–1.33)	0.50 (0.16–1.60)	0.83 (0.43–1.58)	0.52 (0.20–1.35)	0.38 (0.12–1.17)	0.49 (0.23–1.03)	0.68 (0.29–1.63)	**0.18 (0.05–0.72)**
Usually	0.72 (0.38–1.35)	0.66 (0.28–1.55)	0.35 (0.12–1.04)	0.79 (0.44–1.40)	0.70 (0.32–1.53)	0.44 (0.17–1.16)	0.54 (0.28–1.03)	0.85 (0.39–1.85)	0.35 (0.12–1.06)
Always	**0.51 (0.30–0.87)**	0.52 (0.25–1.06)	0.45 (0.19–1.06)	**0.58 (0.36–0.95)**	0.53 (0.27–1.03)	**0.46 (0.21–0.99)**	**0.47 (0.27–0.82)**	**0.34 (0.17–0.67)**	**0.38 (0.16–0.90)**
No new arrivals	**0.53 (0.29–0.97)**	0.71 (0.31–1.64)	**0.29 (0.09–0.88)**	**0.49 (0.28–0.84)**	0.62 (0.30–1.31)	**0.25 (0.09–0.69)**	**0.55 (0.30–1.00)**	**0.46 (0.21–0.98)**	**0.26 (0.09–0.75)**
Home bred replacement ewes (baseline no) Yes	0.78 (0.55–1.12)	**0.55 (0.33–0.89)**	0.82 (0.44–1.52)	x	x	x			
Time to treatment (baseline first day seen lame) <3 days	x	x	x	x	x	x	1.88 (0.95–3.73)	0.94 (0.38–2.35)	3.68 (0.45–30.04)
<7 days	x	x	x	x	x	x	**2.48 (1.22–5.03)**	2.13 (0.85–5.33)	**9.44 (1.15–77.58)**
>7 days	x	x	x	x	x	x	**2.81 (1.19–6.59)**	1.63 (0.54–4.95)	**11.10 (1.20–102.86)**
Did not treat any lame sheep	x	x	x	x	x	x	**0.00 (0.00–0.00**)	**0.00 (0.00–0.00)**	**0.74 (0.74–0.74)**
Number of sheep treated at locomotion score farmer recognized sheep lame (baseline 1 sheep) 2–5	x	x	x	x	x	x	1.19 (0.76–1.86)	**2.78 (1.26–6.10)**	0.46 (0.18–1.18)
6–10	x	x	x	x	x	x	1.52 (0.86–2.67)	**3.85 (1.59–9.29)**	2.34 (0.89–6.15)
>10	x	x	x	x	x	x	**1.88 (1.00–3.51)**	**5.99 (2.36–15.2)**	**2.96 (1.05–8.38)**
Did not treat individuals	x	x	x	x	x	x	2.24 (0.20–28.8)	8.69 (0.56–134)	**0.00 (0.00–0.00)**

*OR, odds ratio; 95% CI, 95% confidence interval (lower, upper); x, management practice not included in model. Odds ratio significantly (p ≤ 0.05) different from the reference category are highlighted in bold (Wald's test). Number and percentage of flocks performing each management practice are found in Supplementary Tables 7a,b, 8*.

Similar to the LC analyses, farmers with ≤2% LiL were less likely to use treatments, whereas farmers with LiL >2% were more likely to use antibiotic injection and foot trimming to treat SFR in lambs and ewes (Table 5, Models 2 and 3, Supplementary Tables 7, 8). Farmers with >10% prevalence of LiE were more likely to delay treatment of lame ewes until >10 sheep in a group were lame compared with one and to treat all sheep >3 days after onset of lameness compared with day 0 (Table 5, Model 3, Supplementary Table 8). In addition, LiL >10% was associated with farmers only recognizing lameness at locomotion score (10) >1 compared with 1 (Table 5). Routine managements that are detrimental to control of lameness (5) were also more frequently used in flocks with higher prevalence of lameness than in flocks with ≤2% lameness: this included farmers more likely to footbath to treat SFR when LiL was >5–10% and >10%, and farmers more likely to vaccinate ewes with Footvax™ to treat footrot when LiL was >2–5% (Table 5). Farmers were also more likely to footbath to treat SFR when LiE >2% and less likely to footbath to prevent ID when LiE >10%. Reduced implementation of biosecurity practices was associated with >2% LiL and LiE (Table 5, Supplementary Tables 7, 8).

## Discussion

This is the first investigation of individual treatment practices and flock managements associated with the prevalence of LiL in the United Kingdom and globally. One highly novel finding was a group of flocks with low prevalence of LiL or ewes that were only sometimes treating sheep lame with footrot (LC1 for lambs and ewes). The most logical explanation for this association is that the causes of lameness were primarily non-infectious; indeed, more farmers in LC1 reported having no ID or SFR lesions in ewes than in other LCs (Table 3). An alternative hypothesis for why lack of treatment was associated with low prevalence of lameness in a flock is that there is a non-linear dynamic infection process in footrot and that at ≤2% prevalence of lameness a low force of infection occurs, and so footrot spreads slowly and prevalence of lameness did not increase. Vaccination would reduce the spread of footrot and flocks with >2–5% or >5–10% LiE were less likely to vaccinate ewes with FootVax™, the commercially available vaccine in the United Kingdom, than those with ≤2% LiE (Table 5, Supplementary Table 8), suggesting it was at least partly effective. Finally, host genetics (33, 34) might have protected flocks sufficiently to reduce the force of infection. Flocks with ≤2% lameness were also not foot trimmed or footbathed, and so feet integrity was protected, and this would have contributed to the low prevalence of lameness (Table 5).

The questionnaire asked farmers for the average prevalence of lameness over 1 year and the treatments used in that same time period. It could be argued that lack of treatment resulted in low prevalence of lameness (reverse causality); however, if infectious lesions were present, and farmers did not use an effective treatment (12), the prevalence of lameness would increase within the 1-year study because of increased incidence and duration of footrot and CODD (13) consequently, reverse causality is an unlikely scenario. In addition, sheep farmers rarely change their managements (35); therefore, it is likely that the responses to management practices were the same as those in the previous year and so practiced before the prevalence of lameness. It is possible that farmers managed their flock differently at certain times during the year, e.g., because of housing, lambing, etc. This was accounted for in the question responses by asking whether a practice was “always, usually, sometimes, or never” performed.

A second novel finding was that no typology was identified where farmers use best practice to treat footrot in lambs. Additionally, farmers with >10% LiL delayed treatment, waited until lambs had more severe locomotion scores, or waited until several sheep in a group were lame before treatment (Model 1). These variables are all correlated (5) and associated with high prevalence of LiE. From the current study, and the infectious nature of footrot, we can conclude that as with ewes (5), prompt treatment of the first mildly lame lamb in a group would reduce the flock prevalence of LiL.

The current British Veterinary Association (BVA) guidelines on appropriate use of antimicrobial products recommend that while use of antimicrobials in farm animals should be minimized, they should be used when appropriate, to treat clinically diseased animals (36). Our results provide evidence that some farmers are using antimicrobial products appropriately to manage lameness. These were farmers with lamb and ewe flocks in LC1 with a low prevalence of infectious causes of lameness where antibiotics were rarely used and ewes in LC2 where “best practice” controlled infectious causes of lameness (12, 13). However, for ewes in flocks in LC3 and LC4 and lambs in flocks in LC2 to LC4, potentially more antibiotic treatments are being used than necessary because they are administered too late to prevent onward spread of infectious causes of lameness.

A third novel finding was that farmers were more likely to practice therapeutic foot trimming of lame lambs in flocks with LiL >10% (Model 1). There was no association between therapeutic foot trimming of ewes and LiL (Model 2), indicating that the effect of trimming feet on lameness applies at an individual level and does not indirectly influence lameness in others in the flock. There is strong evidence that therapeutic foot trimming lame ewes delays recovery from footrot (12) and that foot trimming is associated with development of granulomas (21), which cause chronic lameness, and so the high flock prevalence of LiL associated with foot trimming lambs is not unexpected, but it is useful to have this evidenced. It is encouraging that in flocks where foot trimming is not practiced prevalence of lameness is low (Table 5).

The current guidelines on treatment of lambs with footrot from the BVA via the specialist division of the (37) are to spray with antibiotic injection to treat ID and use injectable antibiotics where there is SFR and to avoid foot trimming. The LC analysis suggested relatively few farmers may be “always” treating SFR in lambs with antibiotic injection, even in LC4 where uptake of antibiotic injection was highest, only 31% of farmers in this LC were “always” using antibiotic injection to treat SFR. Farmers in this LC were also trimming feet with SFR (28% “always”), and stopping this practice would be beneficial.

Considering the evidence base for the efficacy of using “best practice” in ewes in field trials (12, 13) and other studies (5, 38), the current study increases the evidence base that adopting “best practice” to manage footrot (ID and SFR) in lambs would be highly likely to lead to reductions in flock prevalence of lameness. There were no management practices associated with prevalence of LiL that have not previously been associated with LiE. However, those identified could be used to improve control of LiL and so contribute to the FAWC 2021 target of <2% prevalence of lameness in sheep flocks in the United Kingdom. These are discussed briefly below.

Farmers with >2% LiL were less likely to take measures to prevent introduction of disease onto the farm, including introducing new sheep and not quarantining new sheep (Table 5) both previously associated with prevalence of LiE (5, 18). Bringing in new sheep is particularly associated with introduction of CODD (39, 40). Whole flock management practices previously associated with higher prevalence of LiE, and now also associated with higher prevalence of LiL, include feet bleeding during routine foot trimming, culling sheep when persistently lame, and footbathing to treat SFR. Vaccination of ewes is generally used to prevent footrot, but a small proportion of farmers (2.0%) in the current study used vaccination as a treatment (Supplementary Table 1f). There was no association between vaccination of ewes to prevent footrot and prevalence of LiL, but in flocks with >2–5% LiL farmers were more likely to vaccinate ewes to treat SFR than those with <2% LiL, demonstrating a reactive approach to managing lameness rather than a preventive approach and possibly further evidence that using vaccine as a treatment is not effective (41).

The target population for the study was lowland flocks with >200 ewes; this design targeted flocks where the majority of sheep in England are farmed, with 200 being the average number of breeding ewes in a flock in 2011 (42). Therefore, the recommendations from our study are applicable to most sheep in England. Smaller flocks were excluded from the study to avoid identifying managements that could not be scaled up to management of large flocks. There were, however, 206 (16.2%) responses with flocks with <200 ewes. There were no associations between the number of breeding ewes in a flock and prevalence of LiL or LiE in the multinomial models. So, it is likely that the results from our study are also effective in smaller flocks too; however, such flocks might also be managed successfully using different practices.

We have assumed that farmers are as reliable in estimating prevalence of LiL as they are in ewes, where farmers recognize all severities of lameness (43) and estimate prevalence of lameness in their flock with high precision (44). There was a consistent relationship between the prevalence of lameness reported in lambs and ewes (Spearman ρ = 0.62), but distributions were different, suggesting farmers were estimating LiL independently from their ewes. External validation would confirm the assumption that estimation for lambs was equally as reliable as for ewes.

The size of the lamb flocks in the study was not known, preventing analysis of the data on lambs using an overdispersed Poisson model as used by Winter et al. (5) for ewes. The percentage of lame lambs per flock was known, and so consequently, a multinomial model was used. A model for LiE was built to investigate risk factors for ewes using the same family of models and compared with the results of Winter et al. (5). Not as many risk factors for ewes were significant in the multinomial model as in the overdispersed Poisson model, which might be explained by the categorization of the outcome variable, which can result in data loss potentially reducing the power to detect significant associations (45).

## Conclusions

In summary, we identified three distinct flock types and farmer behaviors: (1) low prevalence of lameness with little treatment and avoiding poor management practices; (2) low prevalence of lameness where “best practice” treatment and vaccination of ewes were used, including avoiding foot trimming or footbathing; and (3) high prevalence of lameness associated with foot trimming, footbathing, poor biosecurity, and poor treatment approaches. We conclude that a low flock prevalence of LiL was associated with low prevalence of infectious foot diseases in ewes and avoiding foot trimming and footbathing, which are as detrimental for lambs as ewes. No farmers were treating lambs using current best practice. We conclude that adopting best practice in all flocks, including avoiding foot trimming and footbathing and practicing good biosecurity, would reduce the prevalence of LiL and contribute to the FAWC target of <2% flock prevalence of lameness by 2021.

## Data Availability Statement

The datasets generated for this study will not be made publicly available. The dataset analyzed for this article is not publicly available to protect participant confidentiality as consent was not obtained for the data to become open source at the time of collection.

## Ethics Statement

The studies involving human participants were reviewed and approved by University of Warwick's Biomedical and Scientific Research Ethics Committee (BSREC), reference number BSREC 159-01-12; approved 07 December, 2011. Written informed consent for participation was not required for this study in accordance with the national legislation and the institutional requirements.

## Author Contributions

LG conceived the idea. KL analyzed the data. All authors wrote the paper.

## Conflict of Interest

The authors declare that the research was conducted in the absence of any commercial or financial relationships that could be construed as a potential conflict of interest.
