# Supplementary material for: Management Practices Associated With Prevalence of Lameness in Lambs in 2012–2013 in 1,271 English Sheep Flocks
Source: Front Vet Sci. 2020 Oct 27;7:519601. doi: 10.3389/fvets.2020.519601 (PMC7653190; doi:10.3389/fvets.2020.519601)
Supplement: Supplementary file 1 [file Data_Sheet_1.docx]

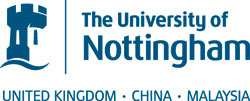

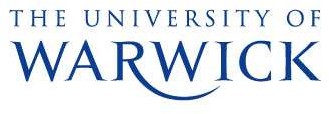
Footrot and scald in sheep: your choices and opinions

## Please ensure that **only one person** answers this questionnaire. Please answer all the questions as accurately and honestly as possible, there are no right or wrong answers to any questions. Your opinions are very valuable and important to us and all answers are entirely confidential.

We would like you to answer all questions on what you did between **May 2012 and April 2013**

**Section 1. Recognising and catching lame sheep**

## The table below describes severity of lameness and gives each level a number between 0 and 6 where 0 is a sound sheep and 6 is the most severely lame sheep. Please answer questions 1 - 3 using this table.

| **Lameness score** | | **Description of sheep when lying, standing and walking** |
| --- | --- | --- |
| **Increasing severity of lameness** | 0 | Sound (not lame), bears weight on all four legs when walking |
|  | 1 | Uneven posture, shortened stride on one leg when walking |
|  | 2 | Visible nodding of head when walking in time with a shortened stride |
|  | 3 | Not weight bearing on affected leg when standing, weight bearing when walking |
|  | 4 | Not weight bearing on affected leg when standing or walking |
|  | 5 | Difficulty rising, reluctant to move, lame on more than one limb |
|  | 6 | Will not stand or move |

1. Using the table above, what is the lowest score you would recognise a sheep as lame?

(*Please circle only one answer*)

**1 2 3 4 5 6**

1. Using the table above, between May 2012 and April 2013 what was the lowest score that you caught an individual lame sheep to treat it? (*Please circle only one answer*)

**Did not treat**

**1 2 3 4 5 6**

**individuals**

1. Between May 2012 and April 2013 how many sheep in the group would have been lame at this score for you to have caught them to treat them? (*Please circle only one answer*)

**1 2-5 6-10 More than 10**

**Did not treat individuals**

1. Between May 2012 and April 2013 when you saw lame sheep how soon did you treat them?

(*Please circle only one answer*)

**The first day you saw them**

**Within three days**

**Within one week**

**Within two weeks**

**Longer than two weeks**

**Did not treat any lame sheep**

1. Generally how easy do you find it to catch an individual lame sheep? (*Please circle only one answer*)

**Very difficult Difficult Neither easy nor**

**difficult**

**Easy Very easy**

1. How do you catch individual lame sheep in your flock *(Please circle all that apply)*

| **Bring group to** | **Use mobile** | **In the** | **Using a dog** | **Using a dog** | **With** | **From a** |
| --- | --- | --- | --- | --- | --- | --- |
| **central handling** | **handling** | **corner of** | **to gather** | **that can catch** | **food** | **vehicle** |
| **facility** | **facility** | **the field** | **the flock** | **individuals** |  |  |

Other *(please state)*

**Section 2. Causes of lameness**

## In the table below, please read the description in the left column and look at the picture and then answer questions 7 and 8 for each lesion.

| What you might notice when you look at the foot | Example picture of the lesion | 7. What did you call this lesion?  *Circle only one answer in each box below* | 8. Did you see this lesion in your flock between May 2012 and April 2013?  *Circle one answer and fill in %*  *for each box below* |
| --- | --- | --- | --- |
| - Red, wet interdigital space - Foul smell - May be grey pasty | 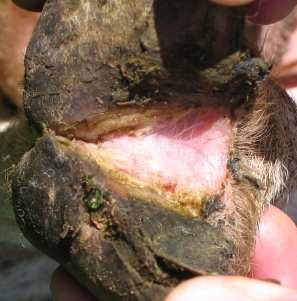 | **Footrot Scald** | **Yes**  ***If yes,* what percentage of ewes had this lesion? % No**  **Do not know** |
| scum   - Loss of hair in |  | **CODD**  **Shelly hoof** |  |
| interdigital space |  |  |  |
|  |  | **Other *(please state)*** |  |
| - Some separation   of horn from | 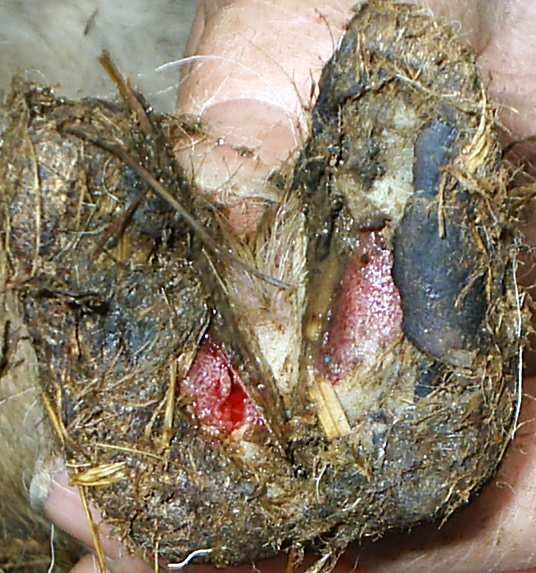 | **Footrot** | **Yes**  ***If yes,* what percentage of ewes had this lesion? % No**  **Do not know** |
| underlying live |  | **Scald** |  |
| foot   - Foul smelling |  | **CODD** |  |
| blackish slimy  dead tissue |  | **Shelly hoof** |  |
|  |  | **Other *(please state)*** |  |
| - Abnormal at   coronary band | 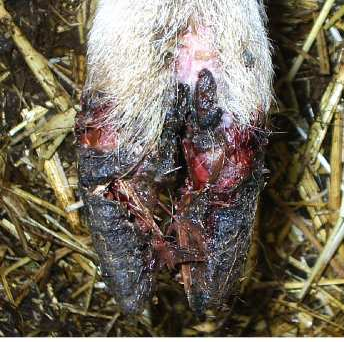 | **Footrot** | **Yes**  ***If yes,* what percentage of ewes had this lesion? % No**  **Do not know** |
| (top of foot)   - Loss of hair above |  | **Scald** |  |
| coronary band   - There may be |  | **CODD**  **Shelly hoof** |  |
| complete |  |  |  |
| detachment of |  | **Other *(please state)*** |  |
| hoof |  |  |  |
| - Some separation   of horn from the | 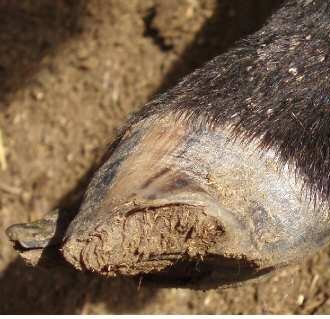 | **Footrot** | **Yes**  ***If yes,* what percentage of ewes had this lesion? % No**  **Do not know** |
| wall, may or may |  | **Scald** |  |
| not see pus   - A pocket |  | **CODD** |  |
| impacted with soil   - Half- moon |  | **Shelly hoof** |  |
| appearance |  | **Other *(please state)*** |  |

**Section 5. Treatment of ewes and lambs with footrot and scald**

## Please complete the table below to tell us about your treatment of sheep with footrot and scald

**between May 2012 and April 2013.** The first row is filled in as an example.

| 13.  Did you | Sheep lame with footrot  ***Please tick one answer for each question*** | | | | Sheep lame with scald  ***Please tick one answer for each question*** | | | |
| --- | --- | --- | --- | --- | --- | --- | --- | --- |
|  | Never | Sometimes | Usually | Always | Never | Sometimes | Usually | Always |
| **e.g. use a turnover crate ?** |  | ✓ |  |  |  | ✓ |  |  |
| Trim the feet of ewes? |  |  |  |  |  |  |  |  |
| Treat ewes with an antibiotic  injection? |  |  |  |  |  |  |  |  |
| Use a foot spray on ewes? |  |  |  |  |  |  |  |  |
| Separate ewes from the flock? |  |  |  |  |  |  |  |  |
| Trim the feet of lambs? |  |  |  |  |  |  |  |  |
| Treat lambs with an antibiotic  injection? |  |  |  |  |  |  |  |  |
| Use a foot spray on lambs? |  |  |  |  |  |  |  |  |
| Other treatments *(please state what you did)* |  |  |  |  |  |  |  |  |

1. Which foot spray did you use to treat sheep with footrot or scald? (*Please circle all that apply*)

**Lincospectin solution Antibiotic aerosol Disinfectant aerosol Did not use**

**Other** *(please state, including product name)*

1. Which injectable antibiotic did you use to treat sheep with footrot or scald? (*Please circle all that apply)*

**Oxytetracycline LA Draxxin PenStrep Did not use**

**Other** *(please state, including product name)*

**Section 6. Routine foot trimming the flock**

1. How many times did you routinely foot trim your flock between May 2012 and April 2013?

(*Please circle only one answer*)

**Never Once Twice More than twice**

*(If Never, please go to Section 7)*

1. Approximately what percentage of sheep did you trim when you did a routine foot trim?

(*Please circle only one answer*)

**Less than 25% 25% 50 % 75% 100%**

1. Approximately what percentage of sheep bled when you did a routine trim? %

**Section 7. Footbathing the flock**

1. Did you footbath your flock between May 2012 and April 2013? (*Please circle all that apply*)

**Yes, ewes Yes, lambs No** *(If No, please go to Section 8)*

1. What did you use footbathing for? (*Please circle all that apply*)

**Treating scald Treating footrot Preventing scald Preventing footrot**

1. What did you use to footbath your sheep? (*Please circle all that apply*)

**Zinc suphate**

**Copper sulphate**

**Formalin Lincospectin Other** *(please state)*

1. When did you footbath your sheep between May 2012 and April 2013? *(Please tick all that apply)*

|  | Before housing | At turnout | Moving  between fields | After gathering | New sheep on arrival | Sheep  returning to the farm | Other  *(please state)* |
| --- | --- | --- | --- | --- | --- | --- | --- |
| Lambs |  |  |  |  |  |  |  |
| Ewes |  |  |  |  |  |  |  |

1. How often did you routinely footbath your ewes at pasture between May 2012 and April 2013?

(*Please circle only one answer*)

**Once a week**

**Once a fortnight**

**Once a month**

**Other** *(please state)* **Did not routinely footbath**

**ewes at pasture**

1. How often did you routinely footbath your lambs at pasture between May 2012 and April 2013?

(*Please circle only one answer*)

**Once a week**

**Once a fortnight**

**Once a month**

**Other** *(please state)* **Did not routinely footbath**

**lambs at pasture**

1. How often did you routinely footbath your ewes when housed between May 2012 and April 2013?

(*Please circle only one answer*)

**Once a week**

**Once a fortnight**

**Once a month**

**Other** *(please state)* **Did not**

**routinely footbath ewes**

**when housed**

**Did not house ewes**

1. How often did you routinely footbath your lambs when housed between May 2012 and April 2013?

(*Please circle only one answer*)

**Once a week**

**Once a fortnight**

**Once a month**

**Other** *(please state)* **Did not**

**routinely footbath lambs**

**when housed**

**Did not house lambs**

**Section 8. Culling and replacement ewes**

1. Did you cull any sheep when they were lame between May 2012 and April 2013?

(*Please circle only one answer*)

**No Yes** *If yes,* **How many?**

1. Did you cull any sheep because they had been lame between May 2012 and April 2013?

(*Please circle only one answer*)

**No Yes** *If yes,* **How many?**

*(If No go to question 31)*

1. Approximately how many times were sheep lame before you culled them?
2. How did you identify sheep for culling that had been lame? (*Please circle all that apply*)

**Memory EID ear tag Non EID tag Coloured spray Other** *(please state)*

1. Did you avoid selecting replacement ewes from mothers that were repeatedly lame?

(*Please circle all that apply*)

**No Yes for my flock Yes for other people’s flocks**

**I did not breed replacement ewe**

**Section 9. Vaccination against footrot**

1. Did you vaccinate any of your sheep with Footvax between May 2012 and April 2013?

(*Please circle all that apply*)

**No Yes**

**Yes**

**Yes sheep**

**Yes bought-in**

**Other** *(please state)*

*(If No, please go to Section 10)*

**ewes**

**rams**

**with footrot**

**sheep**

1. How often did you vaccinate these sheep? (*Please circle all that apply*)

**Once a year**

**Twice a year**

**Before an expected peak in footrot**

**Once in a sheep’s lifetime**

**Other** *(please state)*

**Section 10. Whole flock antibiotic treatment**

1. Did you give all ewes in the flock an antibiotic injection at one time between May 2012 and April 2013? (*Please circle only one answer*)

**Yes No** *(If No, please go to Section 11)*

1. Why was the antibiotic injection given? (*Please circle all that apply*)

**Footrot Toxoplasma abortion Enzootic abortion Other** *(please state)*

1. Which injectable antibiotic did you use? (*Please circle all that apply)*

**Oxytetracycline LA PenStrep Micotil Draxxin Other** *(please state product name)*

**Section 11. Biosecurity**

1. Did you check the feet of sheep before purchase between May 2012 and April 2013?

(*Please circle only one answer*)

**Never Sometimes Usually Always Did not purchase**

1. Did you check the feet of new sheep upon arrival at your farm between May 2012 and April 2013?

(*Please circle only one answer*)

**Never Sometimes Usually Always No new arrivals**

1. Did you treat new sheep with footrot or scald upon arrival between May 2012 and April 2013?

(*Please circle only one answer*)

**Never Sometimes Usually Always No new arrivals**

1. Did you isolate new sheep upon arrival at your farm between May 2012 and April 2013?

(*Please circle only one answer*)

**Never Sometimes Usually Always No new arrivals**

*(If Never, please go (If No new arrivals, please go*

*to question 42) to question 42)*

1. How long did you keep new sheep isolated? (*Please circle only one answer*)

**Less than 1 week Between 1 and 3 weeks More than 3 weeks**

1. Did any of your sheep leave the farm and later return between May 2012 and April 2013?

(*Please circle all that apply*)

| **No** | **Yes, for** | **Yes, when** | **Yes, for** | **Yes, for** | **Yes, back** | **Yes, other** |
| --- | --- | --- | --- | --- | --- | --- |
| *(If No, please go* | **shows** | **sharing** | **summer** | **winter** | **from** | *(please state)* |
| *to question 45)* |  | **rams** | **grazing** | **grazing** | **market** |  |

1. Did you isolate returning sheep coming onto the farm between May 2012 and April 2013?

(*Please circle only one answer*)

**Never Sometimes Usually Always**

*(If Never, please go to question 45)*

1. How long did you keep returning sheep isolated? (*Please circle only one answer*)

**Less than 1 week Between 1 and 3 weeks More than 3 weeks**

1. Did your flock mix with sheep from neighbouring flocks between May 2012 and April 2013?

(*Please circle only one answer*)

**Yes No I do not know**

**Section 18. You, your farm and your flock**

1. For how many years have you been farming sheep? years
2. Are you: **Male Female** *(Please circle one answer)*
3. In which age category are you? (*Please circle only one answer*)

**Less than 25 26-35 36-45 46-55 56-65 over 65**

**Do not wish to say**

1. What percentage of your time did you spend with your flock May 2012 - April 2013 %?
2. How many other people worked (paid or unpaid) full time and part time with your sheep flock between May 2012 and April 2013?
3. What type of land was the majority of your farm between May 2012 and April 2013?

(*Please circle only one answer*)

**Hill Upland Lowland**

1. Was your farm organic between May 2012 and April 2013? (*Please circle only one answer)*

**Yes No Other** *(please state)*

1. Approximately what stocking rate did you use for the ewes between May 2012 and April 2013?

(*Please circle only one answer*)

**Less than 4 ewes per acre**

**4 - 8 ewes per acre**

**More than 8 ewes per acre**

1. Between May 2012 and April 2013 ……..
   1. How many ewes did you have in your breeding flock? ewes
   2. What was the main breed of your flock? breed
   3. What was the average level of lameness in ewes in your flock %
   4. What was the average level of lameness in lambs in your flock %
2. From where did you get replacement ewes between May 2012 and April 2013? (*Please circle all that apply*)

**Bought in Home bred**

1. Between May 2012 and April 2013 which of the following did you produce for sale?

(*Please circle all that apply*)

**Finished lambs Store lambs Breeding stock**

1. Did you house your sheep? (*Please circle all that apply*)

**Yes, ewes Yes, finishing lambs No** *(If No, please go to question 67)*

1. How often did you add fresh bedding to sheep pens? (*Please tick one answer per row*)

|  | **Daily** | **Every 2 days** | **Weekly** | **Other** *(please state)* |
| --- | --- | --- | --- | --- |
| **Ewes** |  |  |  |  |
| **Finishing lambs** |  |  |  |  |

1. What was the condition of the bedding in the sheep pen when you added fresh bedding?

(*Please tick all that apply*)

|  | **Dry** | **Damp** | **Wet** | **Soiled** |
| --- | --- | --- | --- | --- |
| **Ewes** |  |  |  |  |
| **Finishing lambs** |  |  |  |  |

1. Between May 2012 and April 2013 approximately which dates did you house, lamb, wean and sell your sheep? *(Please complete the table below)*

|  | **Start date (DD/MM)** | **End date (DD/MM)** |
| --- | --- | --- |
| **House ewes for winter / lambing** |  |  |
| **Lambing** |  |  |
| **Weaning** |  |  |
| **Selling lambs** |  |  |

**END OF QUESTIONNAIRE**

# Thank you for completing this questionnaire.

**Your help is very much appreciated**

Would you like to be entered into a prize draw for a chance to win one of five prizes of £50 of vouchers of your choice?

### Yes No

We will be sending a second questionnaire in August 2014 and we would like the same person to complete this second questionnaire so please put your name below

**Your name**

Please check and update your details below and include your email address and telephone number if you are willing. All of your details will be kept confidential and will not form any part of this survey. Details provided will not be used for any other purpose or passed on to anyone else.

Tel:

Email

Please return your completed questionnaire in the FREEPOST envelope provided

If you would like to receive the results from this survey and others or participate in further research into lameness or mastitis in sheep please complete the section below

**Research results and further research**

Would you like to receive a summary of the results of this survey when they are available?

### Yes No

Would you like to receive a summary of results from other research when they are available?

### Yes No

Are you interested in helping with future research at the University of Warwick into lameness and mastitis in sheep?

### Yes No

### Supplementary Figure 1: the selected questions relevant to management lameness from the 14-page questionnaire sent to 4000 sheep farmers in England.

**Supplementary Table 1:** Percentage of farmers using each flock management technique in each category of prevalence in lambs in 1271 flocks of sheep in England, 2012-2013.

1. Recognising and catching lame sheep.

| **Variable** | **Responses in lameness prevalence category (%)** | | | | **Total**   **responses** |
| --- | --- | --- | --- | --- | --- |
|  | **≤2%** | **>2-5%** | **>5-10%** | **>10%** |  |
| **Locomotion score farmer recognised sheep** **recognised as lame** | |  |  |  |  |
| 1 | 56.4 | 51.5 | 48.8 | 42.0 | 1266 |
| 2 | 31.5 | 37.0 | 40.1 | 35.0 |  |
| 3 | 11.5 | 10.4 | 6.8 | 16.0 |  |
| 4 or more | 0.7 | 1.1 | 4.3 | 7.0 |  |
| **Minimum locomotion score when farmer decided to treat lame sheep** | | |  |  |  |
| 1 | 29.9 | 24.3 | 20.5 | 29.0 | 1254 |
| 2 | 40.2 | 42.0 | 42.2 | 41.9 |  |
| 3 | 23.0 | 25.9 | 27.3 | 23.7 |  |
| 4 | 4.7 | 5.8 | 8.7 | 4.3 |  |
| 5 | 0.7 | 0.4 | 0.6 | 1.1 |  |
| 6 | 0.0 | 0.2 | 0.0 | 0.0 |  |
| Did not treat individuals | 1.5 | 1.3 | 0.6 | 0.0 |  |
| **Number of sheep lame when treated at minimum locomotion score recognised as lame** | | |  |  |  |
| 1 | 17.6 | 11.8 | 10.6 | 9.5 | 1250 |
| 2-5 | 53.8 | 54.0 | 45.0 | 42.1 |  |
| 6-10 | 15.1 | 17.6 | 24.4 | 24.2 |  |
| >10 | 11.9 | 15.8 | 20.0 | 24.2 |  |
| Did not treat individuals | 1.7 | 0.9 | 0.0 | 0.0 |  |
| **Time to treat lame sheep from first day observed as lame** | | | | |  |
| <1 day | 10.1 | 6.0 | 4.3 | 2.1 | 1256 |
| 1-<3 days | 45.3 | 42.7 | 37.0 | 43.2 |  |
| >3-7 days | 33.5 | 40.5 | 45.1 | 43.2 |  |
| >7 days | 10.4 | 10.6 | 13.6 | 11.6 |  |
| Individuals not treated | 0.7 | 0.2 | 0.0 | 0.0 |  |
| **Ease of catching lame sheep*** | | |  |  |  |
| Very difficult | 3.9 | 4.0 | 2.4 | 6.4 | 1251 |
| Difficult | 29.6 | 27.8 | 31.7 | 24.5 |  |
| Neither easy or difficult | 42.8 | 46.8 | 47.0 | 45.7 |  |
| Easy | 19.4 | 16.6 | 14.0 | 20.2 |  |
| Very easy | 4.3 | 4.9 | 4.9 | 3.2 |  |
| **Used central handling facility to catch lame sheep** | | | |  |  |
| No | 44.7 | 44.1 | 38.8 | 30.9 | 1271 |
| Yes | 55.3 | 55.9 | 61.2 | 69.1 |  |
| **Used mobile handling facility to catch lame sheep** | | | |  |  |
| No | 80.5 | 78.7 | 77.0 | 78.4 | 1271 |
| Yes | 19.5 | 21.3 | 23.0 | 21.7 |  |
| **Used corner of field to catch lame sheep*** | | | |  |  |
| No | 71.1 | 72.4 | 67.3 | 70.1 | 1271 |
| Yes | 28.9 | 27.6 | 32.7 | 29.9 |  |
| **Used a dog that can catch the flock to catch lame sheep** | | | | |  |
| No | 87.7 | 86.2 | 81.8 | 79.4 | 1271 |
| Yes | 12.3 | 13.8 | 18.2 | 20.6 |  |
| **Used a dog that can catch individuals to catch lame sheep*** | |  |  |  |  |
| No | 67.6 | 66.9 | 70.9 | 71.1 | 1271 |
| Yes | 32.4 | 33.1 | 29.1 | 28.9 |  |
| **Used food to catch lame sheep** | | |  |  |  |
| No | 29.4 | 24.0 | 9.2 | 5.4 | 1271 |
| Yes | 14.1 | 11.9 | 3.8 | 2.2 |  |
| **Used a vehicle to catch lame sheep** | | |  |  |  |
| No | 87.2 | 87.5 | 87.9 | 89.7 | 1271 |
| Yes | 12.8 | 12.5 | 12.1 | 10.3 |  |

1. Treating lame ewes and lambs with severe footrot (SFR) and interdigital dermatitis (ID)

| **Variable** | **Responses in lameness prevalence category (%)** | | | | **Total responses** |
| --- | --- | --- | --- | --- | --- |
|  | **<2%** | **>2-5%** | **>5-10%** | **>10%** |  |
| **Treat ewes with SFR with foot trim** | | |  |  |  |
| Never | 5.3 | 2.8 | 1.3 | 0.0 | 1181 |
| Sometimes | 20.7 | 19.8 | 18.1 | 18.9 |  |
| Usually | 31.6 | 35.6 | 35.6 | 35.6 |  |
| Always | 42.4 | 41.8 | 45.0 | 45.6 |  |
| **Treat ewes with SFR with antibiotic injection** | | | |  |  |
| Never | 9.7 | 6.5 | 4.6 | 3.3 | 1178 |
| Sometimes | 40.6 | 44.4 | 40.4 | 41.3 |  |
| Usually | 25.4 | 23.0 | 35.1 | 31.5 |  |
| Always | 24.4 | 26.1 | 19.9 | 23.9 |  |
| **Treat ewes with SFR with foot spray** | | |  |  |  |
| Never | 3.7 | 1.6 | 1.3 | 1.1 | 1196 |
| Sometimes | 10.8 | 8.0 | 10.5 | 12.8 |  |
| Usually | 24.1 | 20.5 | 22.4 | 25.5 |  |
| Always | 61.5 | 69.9 | 65.8 | 60.6 |  |
| **Separate ewes with SFR from the main flock** | | | |  |  |
| Never | 48.9 | 46.9 | 45.8 | 44.7 | 1120 |
| Sometimes | 41.3 | 45.0 | 46.5 | 50.6 |  |
| Usually | 7.6 | 5.5 | 5.6 | 2.4 |  |
| Always | 2.3 | 2.6 | 2.1 | 2.4 |  |
| **Treat lambs with SFR with foot trim** | | |  |  |  |
| Never | 24.2 | 12.8 | 6.3 | 3.5 | 1082 |
| Sometimes | 61.2 | 68.3 | 69.2 | 77.0 |  |
| Usually | 8.0 | 8.0 | 2.7 | 1.8 |  |
| Always | 14.5 | 18.8 | 24.5 | 19.5 |  |
| **Treat lambs with SFR with antibiotic injection** | | | |  |  |
| Never | 27.2 | 22.4 | 15.2 | 15.3 | 1080 |
| Sometimes | 45.9 | 45.1 | 57.2 | 51.8 |  |
| Usually | 13.6 | 16.0 | 13.1 | 12.9 |  |
| Always | 13.4 | 16.5 | 14.5 | 20.0 |  |
| **Treat lambs with SFR foot spray** | | |  |  |  |
| Never | 6.1 | 2.2 | 1.4 | 1.2 | 1094 |
| Sometimes | 17.4 | 13.3 | 10.3 | 9.4 |  |
| Usually | 24.8 | 21.0 | 28.3 | 25.9 |  |
| Always | 51.6 | 63.5 | 60.0 | 63.5 |  |
| **Treat ewes with ID with foot trim** | | |  |  |  |
| Never | 27.9 | 26.8 | 25.4 | 13.6 | 1049 |
| Sometimes | 45.2 | 45.1 | 43.0 | 50.6 |  |
| Usually | 15.4 | 17.3 | 18.3 | 22.2 |  |
| Always | 11.5 | 10.8 | 13.4 | 13.6 |  |
| **Treat ewes with ID with antibiotic injection** | | |  |  |  |
| Never | 49.9 | 49.9 | 48.3 | 37.5 | 1031 |
| Sometimes | 34.2 | 37.5 | 36.6 | 43.8 |  |
| Usually | 9.2 | 8.0 | 11.0 | 16.3 |  |
| Always | 6.7 | 4.6 | 4.1 | 2.5 |  |
| **Treat ewes with ID with foot spray** | | |  |  |  |
| Never | 5.1 | 1.5 | 2.1 | 3.5 | 1098 |
| Sometimes | 11.9 | 11.1 | 9.0 | 14.0 |  |
| Usually | 24.8 | 22.3 | 23.5 | 26.7 |  |
| Always | 58.3 | 65.1 | 65.5 | 55.8 |  |
| **Separate ewes with ID from the main flock** | | |  |  |  |
| Never | 72.7 | 76.2 | 73.3 | 72.0 | 1008 |
| Sometimes | 23.3 | 21.1 | 23.0 | 26.7 |  |
| Usually | 2.8 | 2.6 | 1.5 | 0.0 |  |
| Always | 1.2 | 0.5 | 2.2 | 1.3 |  |
| **Treat lambs with ID with foot trim** | | |  |  |  |
| Never | 41.0 | 34.6 | 32.9 | 22.5 | 1034 |
| Sometimes/Usually | 45.8 | 46.7 | 45.5 | 55.0 |  |
| Usually | 8.6 | 11.1 | 9.1 | 11.3 |  |
| Always | 4.6 | 7.7 | 12.6 | 11.3 |  |
| **Treat lambs with ID with antibiotic injection** | | |  |  |  |
| Never | 53.2 | 53.1 | 51.7 | 40.8 | 1032 |
| Sometimes | 35.5 | 33.9 | 38.6 | 47.4 |  |
| Usually | 7.8 | 8.0 | 5.5 | 4.0 |  |
| Always | 3.5 | 4.9 | 4.1 | 7.9 |  |
| **Treat lambs with ID with foot spray** | | |  |  |  |
| Never | 5.7 | 1.3 | 2.0 | 0.0 | 1087 |
| Sometimes | 18.1 | 14.5 | 9.1 | 13.4 |  |
| Usually | 21.8 | 21.3 | 26.6 | 22.0 |  |
| Always | 54.6 | 62.9 | 62.3 | 64.6 |  |
| **Use Lincospectin solution foot spray** | | |  |  |  |
| No | 92.2 | 89.0 | 87.3 | 81.4 | 1271 |
| Yes | 7.8 | 11.0 | 12.7 | 18.6 |  |
| **Use antibiotic aerosol foot spray** | |  |  |  |  |
| No | 16.5 | 12.3 | 11.5 | 7.2 | 1271 |
| Yes | 83.5 | 87.7 | 88.5 | 92.8 |  |
| **Use disinfectant aerosol foot spray** | | |  |  |  |
| No | 87.5 | 86.8 | 88.5 | 91.8 | 1271 |
| Yes | 12.5 | 13.2 | 11.5 | 8.3 |  |
| **Did not use foot spray** | |  |  |  |  |
| No | 98.0 | 99.3 | 98.8 | 100.0 | 1271 |
| Yes | 2.0 | 0.7 | 1.2 | 0.0 |  |
| **Use oxytetracycline LA injectable antibiotic** | | |  |  |  |
| No | 40.1 | 38.8 | 35.8 | 32.0 | 1271 |
| Yes | 59.9 | 61.2 | 64.2 | 68.0 |  |
| **Use Draxxin injectable antibiotic** | |  |  |  |  |
| No | 97.8 | 98.7 | 100.0 | 97.9 | 1271 |
| Yes | 2.2 | 1.3 | 0.0 | 2.1 |  |
| **Used PenStrep injectable antibiotic** | | |  |  |  |
| No | 65.5 | 64.5 | 65.5 | 69.1 | 1271 |
| Yes | 34.5 | 35.5 | 34.6 | 30.9 |  |
| **Did not use injectable antibiotics** | |  |  |  |  |
| No | 93.5 | 93.4 | 96.4 | 97.9 | 1271 |
| Yes | 6.5 | 6.6 | 3.6 | 2.1 |  |

1. Routine foot trim the flock

| **Variable** | **Responses in lameness prevalence category (%)** | | | | **Total responses** |
| --- | --- | --- | --- | --- | --- |
|  | **≤2%** | **>2-5%** | **>5-10%** | **>10%** |  |
| **Routine foot trim the flock** | |  |  |  |  |
| Did not trim | 46.2 | 42.3 | 48.4 | 25.5 | 1206 |
| Trimmed but no bleeding | 8.8 | 5.5 | 4.6 | 5.3 |  |
| Caused bleeding | 45.1 | 52.2 | 47.1 | 69.2 |  |

1. Footbath the flock

| **Variable** | **Responses in lameness prevalence category (%)** | | | | **Total responses** |
| --- | --- | --- | --- | --- | --- |
|  | **≤2%** | **>2-5%** | **>5-10%** | **>10%** |  |
| **Footbath ewes** |  |  |  |  |  |
| No | 46.5 | 37.3 | 33.3 | 27.8 | 1271 |
| Yes | 53.5 | 62.7 | 66.7 | 72.2 |  |
| **Footbath lambs** |  |  |  |  |  |
| No | 52.4 | 39.3 | 33.9 | 28.9 | 1271 |
| Yes | 47.6 | 60.8 | 66.1 | 71.1 |  |
| **Did not footbath** |  |  |  |  |  |
| No | 57.5 | 66.9 | 71.5 | 76.3 | 1271 |
| Yes | 42.5 | 33.1 | 28.5 | 23.7 |  |
| **Footbath to treat ID** | |  |  |  |  |
| No | 59.0 | 50.4 | 35.2 | 38.1 | 1271 |
| Yes | 41.1 | 49.6 | 64.9 | 61.9 |  |
| **Footbath to treat SFR** | |  |  |  |  |
| No | 69.6 | 62.5 | 60.6 | 45.4 | 1271 |
| Yes | 30.4 | 37.5 | 39.4 | 54.6 |  |
| **Footbath to prevent ID** | |  |  |  |  |
| No | 66.4 | 62.7 | 61.2 | 66.0 | 1271 |
| Yes | 33.6 | 37.3 | 38.8 | 34.0 |  |
| **Footbath to prevent SFR** | |  |  |  |  |
| No | 65.1 | 63.8 | 66.1 | 60.8 |  |
| Yes | 34.9 | 36.2 | 33.9 | 39.2 | 1271 |
| **Use zinc sulphate to footbath** | |  |  |  |  |
| No | 80.3 | 77.4 | 80.0 | 73.2 |  |
| Yes | 19.7 | 22.6 | 20.0 | 26.8 | 1271 |
| **Used copper sulphate to footbath** | | |  |  |  |
| No | 95.3 | 93.0 | 92.7 | 93.8 |  |
| Yes | 4.7 | 7.0 | 7.3 | 6.2 | 1271 |
| **Use Formalin to footbath** | |  |  |  |  |
| No | 60.8 | 50.9 | 41.8 | 47.4 |  |
| Yes | 39.2 | 49.1 | 58.2 | 52.6 | 1271 |
| **Use Lincospectin to footbath** | |  |  |  |  |
| No | 92.2 | 89.3 | 86.7 | 83.5 |  |
| Yes | 7.8 | 10.8 | 13.3 | 16.5 | 1271 |
| **Footbath lambs before housing** | | |  |  |  |
| No | 93.9 | 93.6 | 88.5 | 89.7 |  |
| Yes | 6.2 | 6.4 | 11.5 | 10.3 | 1271 |
| **Footbath lambs at turnout** | |  |  |  |  |
| No | 97.5 | 96.1 | 98.2 | 95.9 |  |
| Yes | 2.5 | 4.0 | 1.8 | 4.1 | 1271 |
| **Footbath lambs when moving field** | | |  |  |  |
| No | 82.5 | 79.0 | 75.2 | 72.2 |  |
| Yes | 17.5 | 21.1 | 24.9 | 27.8 | 1271 |
| **Footbath lambs after gathering** | | |  |  |  |
| No | 74.9 | 71.9 | 59.4 | 55.7 |  |
| Yes | 25.1 | 28.1 | 40.6 | 44.3 | 1271 |
| **Footbath lambs when new sheep arrived on farm** | | |  |  |  |
| No | 91.7 | 92.3 | 86.7 | 90.7 | 1271 |
| Yes | 8.3 | 7.7 | 13.3 | 9.3 |  |
| **Footbath lambs when sheep returned to the farm** | | |  |  |  |
| No | 94.0 | 94.5 | 89.7 | 87.6 | 1271 |
| Yes | 6.0 | 5.5 | 10.3 | 12.4 |  |
| **Footbath ewes before housing** | |  |  |  |  |
| No | 78.7 | 77.9 | 74.6 | 80.4 | 1271 |
| Yes | 21.3 | 22.2 | 25.5 | 19.6 |  |
| **Footbath ewes at housing** | |  |  |  |  |
| No | 95.1 | 95.2 | 95.2 | 95.9 | 1271 |
| Yes | 4.9 | 4.8 | 4.9 | 4.1 |  |
| **Footbath ewes at turnout*** |  |  |  |  |  |
| No | 95.1 | 95.2 | 95.2 | 95.9 | 1271 |
| Yes | 5.6 | 5.7 | 6.0 | 5.6 |  |
| **Footbath ewes when moving fields** | | |  |  |  |
| No | 82.6 | 80.3 | 76.4 | 69.1 | 1271 |
| Yes | 17.4 | 19.7 | 23.6 | 30.9 |  |
| **Footbath ewes after gathering** | | |  |  |  |
| No | 75.4 | 69.1 | 61.2 | 59.8 | 1271 |
| Yes | 24.6 | 30.9 | 38.8 | 40.2 |  |
| **Footbath new sheep at arrival** | | |  |  |  |
| No | 83.9 | 83.8 | 77.6 | 81.4 | 1271 |
| Yes | 16.1 | 16.2 | 22.4 | 18.6 |  |
| **Footbath ewes when sheep returned to the farm** | | |  |  |  |
| No | 91.9 | 91.7 | 86.7 | 86.6 | 1271 |
| Yes | 8.1 | 8.3 | 13.3 | 13.4 |  |
| **Frequency of routine footbathing of ewes at pasture** | | | |  |  |
| Once a week | 0.9 | 2.0 | 0.9 | 0.0 | 827 |
| Once a fortnight | 10.6 | 8.5 | 9.4 | 10.7 |  |
| Once a month | 21.9 | 31.4 | 26.5 | 37.3 |  |
| Other | 26.8 | 23.9 | 25.6 | 21.3 |  |
| Did not do routinely | 39.8 | 34.3 | 37.6 | 30.7 |  |
| **Frequency of routine footbathing of lambs at pasture** | | | |  |  |
| Once a week | 0.9 | 2.3 | 5.0 | 2.6 | 833 |
| Once a fortnight | 10.9 | 11.4 | 11.8 | 14.5 |  |
| Once a month | 20.2 | 28.3 | 27.7 | 31.6 |  |
| Other | 22.4 | 21.2 | 21.0 | 26.3 |  |
| Did not do routinely | 45.6 | 36.8 | 34.5 | 25.0 |  |
| **Frequency of routine footbathing of ewes when housed** | | | |  |  |
| Once a week | 6.6 | 3.0 | 5.4 | 2.9 | 781 |
| Once a fortnight | 6.9 | 7.1 | 10.7 | 4.4 |  |
| Once a month | 10.5 | 12.2 | 14.3 | 14.7 |  |
| Other | 0.3 | 0.7 | 0.9 | 1.5 |  |
| Did not do routinely | 51.8 | 48.7 | 45.5 | 50.0 |  |
| Did not house ewes | 23.9 | 28.4 | 23.2 | 26.5 |  |
| **Frequency of routine footbathing of lambs when housed** | | | | |  |
| Once a week | 3.7 | 3.8 | 4.6 | 5.5 | 772 |
| Once a fortnight | 5.4 | 4.8 | 10.1 | 6.9 |  |
| Once a month | 2.7 | 4.5 | 5.5 | 4.1 |  |
| Other | 0.3 | 1.0 | 0.0 | 0.0 |  |
| Did not do routinely | 27.8 | 21.3 | 21.1 | 31.5 |  |
| Did not house lambs | 60.2 | 64.6 | 58.7 | 52.1 |  |

1. Culling and replacing ewes

| **Variable** | **Responses in lameness prevalence category (%)** | | | | **Total responses** |
| --- | --- | --- | --- | --- | --- |
|  | **≤2%** | **>2-5%** | **>5-10%** | **>10%** |  |
| **Culling of ewes** |  |  |  |  |  |
| Did not cull when lame | 51.4 | 52.1 | 45.2 | 46.9 | 1085 |
| Had been lame once | 5.7 | 2.2 | 2.6 | 0.0 |  |
| Had been lame twice | 15.8 | 11.1 | 12.3 | 11.1 |  |
| Had been lame 2 or more times | 23.5 | 27.8 | 36.1 | 33.3 |  |
| If persistently lame | 3.6 | 6.9 | 3.9 | 8.6 |  |
| **Identified sheep for culling using memory*** | | |  |  |  |
| No | 86.8 | 88.2 | 82.4 | 81.4 | 1271 |
| Yes | 13.2 | 11.8 | 17.6 | 18.6 |  |
| **Identified sheep for culling with an EID ear tag** | | | |  |  |
| No | 93.3 | 96.5 | 92.7 | 96.9 | 1271 |
| Yes | 6.7 | 3.5 | 7.3 | 3.1 |  |
| **Identified sheep for culling with a non-EID ear tag** | | | |  |  |
| No | 89.9 | 87.9 | 86.1 | 86.6 | 1271 |
| Yes | 10.1 | 12.1 | 13.9 | 13.4 |  |
| **Identified sheep for culling using coloured spray** | | | |  |  |
| No | 64.0 | 62.5 | 61.8 | 60.8 | 1271 |
| Yes | 36.0 | 37.5 | 38.2 | 39.2 |  |
| **Did not avoid selecting replacement ewes from repeatedly lame mothers*** | | | | | |
| No | 54.6 | 56.4 | 58.2 | 53.6 | 1271 |
| Yes | 45.4 | 43.6 | 41.8 | 46.4 |  |
| **Avoided selecting replacement ewes from repeatedly lame mothers for own flock** | | | | | |
| No | 72.2 | 72.8 | 70.3 | 68.0 | 1271 |
| Yes | 27.9 | 27.2 | 29.7 | 32.0 |  |
| **Avoided selecting replacement ewes from repeatedly lame mothers for other flock** | | | | | |
| No | 96.2 | 97.2 | 95.8 | 97.9 | 1271 |
| Yes | 3.8 | 2.9 | 4.2 | 2.1 |  |
| **Did not breed replacement ewes** | |  |  |  |  |
| No | 78.1 | 72.4 | 77.0 | 76.3 | 1271 |
| Yes | 21.9 | 27.6 | 23.0 | 23.7 |  |

1. Vaccinating the flock with FootVax

| **Variable** | **Responses in lameness prevalence category (%)** | | | | **Total responses** |
| --- | --- | --- | --- | --- | --- |
|  | **≤2%** | **>2-5%** | **>5-10%** | **>10%** |  |
| **Did not vaccinate sheep with FootVax** | | |  |  |  |
| No | 24.1 | 22.4 | 26.7 | 21.7 | 1271 |
| Yes | 76.0 | 77.6 | 73.3 | 78.4 |  |
| **Vaccinated ewes with FootVax** | | |  |  |  |
| No | 83.4 | 84.2 | 80.0 | 84.5 | 1271 |
| Yes | 16.6 | 15.8 | 20.0 | 15.5 |  |
| **Vaccinate rams with FootVax** | | |  |  |  |
| No | 81.0 | 82.7 | 79.4 | 82.5 | 1271 |
| Yes | 19.0 | 17.3 | 20.6 | 17.5 |  |
| **Vaccinate sheep with footrot with FootVax** | | | |  |  |
| No | 98.0 | 96.3 | 97.0 | 95.9 | 1271 |
| Yes | 2.0 | 3.7 | 3.0 | 4.1 |  |
| **Vaccinate bought in sheep with FootVax** | | | |  |  |
| No | 93.0 | 92.3 | 95.8 | 94.9 | 1271 |
| Yes | 7.1 | 7.7 | 4.2 | 5.2 |  |
| **Vaccinate with FootVax for other purposes** | | | |  |  |
| No | 99.8 | 99.8 | 100.0 | 100.0 | 1271 |
| Yes | 0.2 | 0.2 | 0.0 | 0.0 |  |
| **Vaccinate once a year** | |  |  |  |  |
| No | 83.4 | 84.0 | 82.4 | 82.5 | 1271 |
| Yes | 16.6 | 16.0 | 17.6 | 17.5 |  |
| **Vaccinate twice a year** | |  |  |  |  |
| No | 95.8 | 96.5 | 93.3 | 96.9 | 1271 |
| Yes | 4.2 | 3.5 | 6.7 | 3.1 |  |
| **Vaccinate before an expected peak in footrot** | | |  |  |  |
| No | 98.4 | 98.0 | 97.0 | 96.9 | 1271 |
| Yes | 1.6 | 2.0 | 3.0 | 3.1 |  |
| **Vaccinate once in a sheep's lifetime** | | |  |  |  |
| No | 98.0 | 97.2 | 100.0 | 100.0 | 1271 |
| Yes | 2.0 | 2.9 | 0.0 | 0.0 |  |
| **Vaccinate at other frequency** | | |  |  |  |
| No | 100.0 | 99.6 | 100.0 | 100.0 | 1271 |
| Yes | 0.0 | 0.4 | 0.0 | 0.0 |  |

1. Whole flock antibiotic treatment

| **Variable** | **Responses in lameness prevalence category (%)** | | | | **Total responses** |
| --- | --- | --- | --- | --- | --- |
|  | **<2%** | **>2-5%** | **>5-10%** | **>10%** |  |
| **Were all ewes given an antibiotic injection at one time?** | | | |  |  |
| Yes | 5.9 | 7.5 | 9.2 | 9.4 | 1256 |
| No | 94.1 | 92.5 | 90.9 | 90.6 |  |
| **Use whole flock antibiotic injection for footrot** | | | |  |  |
| No | 96.4 | 95.0 | 95.2 | 93.8 | 1271 |
| Yes | 3.6 | 5.0 | 4.9 | 6.2 |  |
| **Use whole flock antibiotic injection for toxoplasma** | | | |  |  |
| No | 97.5 | 96.3 | 96.4 | 97.9 | 1271 |
| Yes | 2.5 | 3.7 | 3.6 | 2.1 |  |
| **Use whole flock antibiotic injection for enzootic abortion** | | | | |  |
| No | 95.3 | 95.4 | 93.9 | 92.8 | 1271 |
| Yes | 4.7 | 4.6 | 6.1 | 7.2 |  |
| **Use whole flock antibiotic injection for other reason** | | | |  |  |
| No | 98.2 | 98.5 | 98.2 | 97.9 | 1271 |
| Yes | 1.8 | 1.5 | 1.8 | 2.1 |  |
| **Use oxytetracycline LA to treat the whole flock** | | | |  |  |
| No | 93.9 | 92.3 | 90.3 | 86.6 | 1271 |
| Yes | 6.2 | 7.7 | 9.7 | 13.4 |  |
| **Use PenStrep to treat the whole flock** | | |  |  |  |
| No | 97.3 | 98.0 | 98.2 | 99.0 | 1271 |
| Yes | 2.7 | 2.0 | 1.8 | 1.0 |  |
| **Use Micotil to treat the whole flock** | | |  |  |  |
| No | 99.3 | 99.3 | 98.8 | 100.0 |  |
| Yes | 0.7 | 0.7 | 1.2 | 0.0 |  |
| **Use Draxxin to treat the whole flock** | | |  |  |  |
| No | 99.8 | 99.6 | 100.0 | 99.0 | 1271 |
| Yes | 0.2 | 0.4 | 0.0 | 1.0 |  |
| **Use another injectable antibiotic to treat the whole flock** | | | | |  |
| No | 98.7 | 96.9 | 98.2 | 97.9 | 1271 |
| Yes | 1.3 | 3.1 | 1.8 | 2.1 |  |

1. Biosecurity

| **Variable** | **Responses in lameness prevalence category (%)** | | | | **Total responses** |
| --- | --- | --- | --- | --- | --- |
|  | **≤2%** | **>2-5%** | **>5-10%** | **>10%** |  |
| **Check the feet of sheep before purchase** | |  |  |  |  |
| Never | 13.4 | 16.7 | 15.7 | 16.5 | 1252 |
| Sometimes | 12.8 | 16.0 | 14.5 | 19.6 |  |
| Usually | 16.8 | 24.3 | 19.5 | 27.8 |  |
| Always | 32.2 | 24.7 | 27.7 | 20.6 |  |
| Did not purchase new sheep | 24.9 | 18.3 | 22.6 | 15.5 |  |
| **Check the feet of new sheep on arrival to the farm*** | | |  |  |  |
| Never | 11.0 | 13.5 | 12.3 | 10.5 | 1232 |
| Sometimes | 11.9 | 13.0 | 11.6 | 14.7 |  |
| Usually | 16.0 | 24.7 | 20.0 | 29.5 |  |
| Always | 36.8 | 30.5 | 36.8 | 31.6 |  |
| Did not purchase new sheep | 24.3 | 18.4 | 19.4 | 13.7 |  |
| **Treat feet of new sheep with ID/SFR at arrival** | | |  |  |  |
| Never | 27.6 | 27.2 | 28.0 | 25.3 | 1187 |
| Sometimes | 15.6 | 19.0 | 16.0 | 17.6 |  |
| Usually | 7.9 | 12.6 | 10.7 | 12.1 |  |
| Always | 24.0 | 22.8 | 24.7 | 30.8 |  |
| Did not purchase new sheep | 25.0 | 18.5 | 20.7 | 14.3 |  |
| **Isolation of new sheep at arrival** | |  |  |  |  |
| Never | 10.5 | 13.2 | 15.3 | 14.7 | 1235 |
| Sometimes | 7.5 | 10.1 | 7.0 | 8.4 |  |
| Usually | 12.7 | 15.7 | 17.2 | 17.9 |  |
| Always | 46.6 | 43.6 | 40.8 | 45.3 |  |
| Did not purchase new sheep | 22.8 | 17.5 | 19.8 | 13.7 |  |
| **Length of time of isolation of new sheep*** | |  |  |  |  |
| <1 week | 8.8 | 8.5 | 13.3 | 15.2 | 852 |
| 1-3 weeks | 49.5 | 54.3 | 45.7 | 54.6 |  |
| >3 weeks | 41.8 | 37.2 | 41.0 | 30.3 |  |
| **No sheep left and returned to the farm** | |  |  |  |  |
| No | 37.6 | 37.5 | 46.1 | 50.5 | 1271 |
| Yes | 62.4 | 62.5 | 53.9 | 49.5 |  |
| **Sheep returned to farm from shows** | |  |  |  |  |
| No | 95.5 | 95.2 | 92.7 | 93.8 | 1271 |
| Yes | 4.5 | 4.8 | 7.3 | 6.2 |  |
| **Sheep returned to farm from sharing rams** | | |  |  |  |
| No | 98.2 | 97.6 | 97.0 | 99.0 | 1271 |
| Yes | 1.8 | 2.4 | 3.0 | 1.0 |  |
| **Sheep returned to farm from summer grazing*** | | |  |  |  |
| No | 87.0 | 84.7 | 84.9 | 78.4 | 1271 |
| Yes | 13.0 | 15.4 | 15.2 | 21.7 |  |
| **Sheep returned to farm from winter grazing** | | |  |  |  |
| No | 76.0 | 75.4 | 67.9 | 66.0 | 1271 |
| Yes | 24.1 | 24.6 | 32.1 | 34.0 |  |
| **Sheep returned to farm from market*** | |  |  |  |  |
| No | 96.9 | 96.7 | 97.6 | 93.8 | 1271 |
| Yes | 3.1 | 3.3 | 2.4 | 6.2 |  |
| **Sheep returned to farm for other reasons** | |  |  |  |  |
| No | 99.1 | 99.3 | 100.0 | 97.9 | 1271 |
| Yes | 0.9 | 0.7 | 0.0 | 2.1 |  |
| **Isolation of returning sheep** | |  |  |  |  |
| Never | 50.5 | 57.1 | 45.1 | 66.1 | 558 |
| Sometimes | 16.1 | 16.3 | 24.4 | 12.5 |  |
| Usually | 20.1 | 14.8 | 18.3 | 17.9 |  |
| Always | 13.4 | 11.7 | 12.2 | 3.6 |  |
| **Length of time of isolation of returning sheep** | |  |  |  |  |
| <1 week | 19.5 | 24.4 | 23.4 | 5.0 | 275 |
| 1-3 weeks | 56.8 | 52.2 | 59.6 | 75.0 |  |
| >3 weeks | 23.7 | 23.3 | 17.0 | 20.0 |  |
| **Did the flock mix with neighbouring flocks?** | |  |  |  |  |
| Yes | 3.5 | 6.3 | 8.1 | 2.1 | 1238 |
| No | 95.7 | 93.0 | 91.9 | 97.9 |  |
| Unknown | 0.8 | 0.7 | 0.0 | 0.0 |  |

1. Farm and farmer characteristics

| **Variable** | **Responses in lameness prevalence category (%)** | | | | **Total responses** |
| --- | --- | --- | --- | --- | --- |
|  | **≤2%** | **>2-5%** | **>5-10%** | **>10%** |  |
| **Farmer sex** |  |  |  |  |  |
| Male | 84.4 | 87.1 | 90.9 | 81.7 | 1259 |
| Female | 15.6 | 12.9 | 9.1 | 18.3 |  |
| **Farmer age** |  |  |  |  |  |
| <25 | 1.8 | 2.2 | 1.8 | 0.0 | 1264 |
| 26-35 | 6.5 | 5.3 | 8.5 | 5.2 |  |
| 36-45 | 12.7 | 15.9 | 17.1 | 17.7 |  |
| 46-55 | 33.8 | 33.8 | 30.5 | 31.3 |  |
| 56-65 | 27.0 | 24.5 | 27.4 | 29.2 |  |
| >65 | 17.8 | 17.7 | 14.6 | 16.7 |  |
| Do not wish to say | 0.4 | 0.7 | 0.0 | 0.0 |  |
| **Land type** |  |  |  |  |  |
| Hill | 3.3 | 3.4 | 5.0 | 3.2 | 1249 |
| Upland | 10.3 | 11.2 | 13.7 | 13.7 |  |
| Lowland | 86.4 | 85.5 | 81.4 | 83.2 |  |
| **Organic status** | |  |  |  |  |
| Yes | 8.4 | 2.7 | 3.7 | 1.0 | 1256 |
| No | 91.6 | 97.3 | 96.3 | 99.0 |  |
| **Ewe stocking rate** | |  |  |  |  |
| <4 ewes/acre | 45.0 | 47.8 | 45.2 | 32.6 | 1229 |
| 4-8 ewes/acre | 50.3 | 48.2 | 51.6 | 61.1 |  |
| >8 ewes/acre | 4.7 | 4.0 | 3.2 | 6.3 |  |
| **Bought in replacement ewes** | | |  |  |  |
| No | 47.9 | 37.7 | 46.1 | 35.1 | 1271 |
| Yes | 52.1 | 62.3 | 53.9 | 65.0 |  |
| **Home bred replacement ewes** | | |  |  |  |
| No | 28.9 | 36.8 | 33.9 | 39.2 | 1271 |
| Yes | 71.1 | 63.2 | 66.1 | 60.8 |  |
| **Production of finished lambs** | | |  |  |  |
| No | 8.0 | 9.7 | 5.5 | 7.2 | 1271 |
| Yes | 92.0 | 90.4 | 94.6 | 92.8 |  |
| **Production of store lambs** | | |  |  |  |
| No | 70.5 | 72.6 | 77.0 | 72.2 | 1271 |
| Yes | 29.5 | 27.4 | 23.0 | 27.8 |  |
| **Production of breeding stock*** | | |  |  |  |
| No | 69.1 | 73.5 | 73.9 | 76.3 | 1271 |
| Yes | 30.9 | 26.5 | 26.1 | 23.7 |  |
| **Farm housed ewes** | |  |  |  |  |
| No | 26.6 | 29.0 | 29.1 | 23.7 | 1271 |
| Yes | 73.4 | 71.1 | 70.9 | 76.3 |  |
| **Farm housed lambs** | |  |  |  |  |
| No | 80.7 | 80.9 | 79.4 | 76.3 | 1271 |
| Yes | 19.4 | 19.1 | 20.6 | 23.7 |  |
| **Did not house sheep** | |  |  |  |  |
| No | 76.7 | 72.8 | 74.6 | 77.3 | 1271 |
| Yes | 23.3 | 27.2 | 25.5 | 22.7 |  |
| **Frequency of adding bedding to ewe pens** | | |  |  |  |
| Daily | 37.1 | 37.0 | 32.6 | 30.1 | 858 |
| Every 2 weeks | 30.3 | 31.5 | 36.1 | 30.1 |  |
| Weekly | 8.7 | 8.6 | 6.3 | 10.8 |  |
| Other | 0.6 | 1.0 | 0.7 | 1.1 |  |
| **Frequency of adding bedding to lamb pens** | | |  |  |  |
| Daily | 16.8 | 24.1 | 28.6 | 15.4 | 261 |
| Every 2 weeks | 43.4 | 37.9 | 37.1 | 38.5 |  |
| Weekly | 38.9 | 35.6 | 31.4 | 42.3 |  |
| Other | 0.9 | 2.3 | 2.9 | 3.9 |  |
| **Bedding condition when fresh added to ewe pens** | | | |  |  |
| ***Dry*** |  |  |  |  |  |
| No | 56.8 | 59.9 | 63.6 | 58.8 | 1271 |
| Yes | 43.2 | 40.1 | 36.4 | 41.2 |  |
| ***Damp*** |  |  |  |  |  |
| No | 70.9 | 70.8 | 67.9 | 68.0 | 1271 |
| Yes | 29.1 | 29.2 | 32.1 | 32.0 |  |
| ***Wet*** |  |  |  |  |  |
| No | 98.2 | 98.3 | 99.4 | 99.0 | 1271 |
| Yes | 1.8 | 1.8 | 0.6 | 1.0 |  |
| ***Soiled*** |  |  |  |  |  |
| No | 83.5 | 83.1 | 83.6 | 90.7 | 1271 |
| Yes | 16.5 | 16.9 | 16.4 | 9.3 |  |
| **Bedding condition when fresh added to lamb pens** | | | |  |  |
| ***Dry*** |  |  |  |  |  |
| No | 87.9 | 89.7 | 89.7 | 87.6 | 1271 |
| Yes | 12.1 | 10.3 | 10.3 | 12.4 |  |
| ***Damp*** |  |  |  |  |  |
| No | 93.0 | 91.2 | 89.1 | 85.6 | 1271 |
| Yes | 7.1 | 8.8 | 10.9 | 14.4 |  |
| ***Wet*** |  |  |  |  |  |
| No | 99.8 | 99.3 | 98.8 | 100.0 | 1271 |
| Yes | 0.2 | 0.7 | 1.2 | 0.0 |  |
| ***Soiled*** |  |  |  |  |  |
| No | 94.9 | 95.4 | 96.4 | 94.9 | 1271 |
| Yes | 5.1 | 4.6 | 3.6 | 5.2 |  |

*: Risk factor identified as significantly associated with prevalence of lameness in ewes by Winter et al., (2015), but not by this paper.

1. Number of responses, arithmetic mean and 95% confidence interval within each lamb lameness category for continuous variables collected, for 1271 sheep flocks in England, 2012-2013

| **Continuous Variables** | **Prevalence of lameness (%)** | **Mean** | **95% CI** | | **Total responses** |
| --- | --- | --- | --- | --- | --- |
| Average annual prevalence of ewe lameness | ≤2 | 3.0 | 2.8 | 3.3 | 1271 |
|  | >2-5 | 5.3 | 4.9 | 5.6 |  |
|  | >5-10 | 8.1 | 7.2 | 9.1 |  |
|  | >10 | 12.4 | 11.0 | 13.9 |  |
| Average annual prevalence of lamb lameness | ≤2 | 1.4 | 1.3 | 1.4 | 1271 |
|  | >2-5 | 4.2 | 4.1 | 4.3 |  |
|  | >5-10 | 8.8 | 8.6 | 9.1 |  |
|  | >10 | 21.0 | 18.8 | 23.2 |  |
| Farmer experience (years) | ≤2 | 32.4 | 31.2 | 33.5 | 1251 |
|  | >2-5 | 33.4 | 32.2 | 34.6 |  |
|  | >5-10 | 31.9 | 29.9 | 33.8 |  |
|  | >10 | 32.9 | 30.3 | 35.5 |  |
| Farmer time spent with flock (%) | ≤2 | 40.5 | 38.3 | 42.6 | 1189 |
|  | >2-5 | 41.4 | 39.0 | 43.7 |  |
|  | >5-10 | 46.4 | 42.4 | 50.3 |  |
|  | >10 | 49.0 | 43.8 | 54.1 |  |
| Ewe numbers* | ≤2 | 461.3 | 421.6 | 500.9 | 1271 |
|  | >2-5 | 438.4 | 402.3 | 474.4 |  |
|  | >5-10 | 480.1 | 418.0 | 542.3 |  |
|  | >10 | 476.7 | 352.5 | 600.8 |  |
| Number of full-time employees | ≤2 | 0.7 | 0.7 | 0.8 | 819 |
|  | >2-5 | 0.8 | 0.7 | 0.8 |  |
|  | >5-10 | 0.8 | 0.7 | 0.9 |  |
|  | >10 | 0.8 | 0.6 | 0.9 |  |
| Number of part-time employees | ≤2 | 1.4 | 1.3 | 1.5 | 1079 |
|  | >2-5 | 1.3 | 1.2 | 1.4 |  |
|  | >5-10 | 1.3 | 1.2 | 1.5 |  |
|  | >10 | 1.4 | 1.1 | 1.8 |  |

**Supplementary Table 2:** Univariable multinomial models of factors associated with prevalence of lameness in lambs in 1271 flocks of sheep in England, 2012-2013.

| **Prevalence of lameness and variable** | | **N** | **%** | | **OR** | | **Upper and lower 95% CI** | | **P value** |
| --- | --- | --- | --- | --- | --- | --- | --- | --- | --- |
| Locomotion score farmer recognised sheep as lame at | | | | |  | |  |  |  |
| ≤2% | 1 | 310 | 56.4 | | Reference category | | |  |  |
|  | 2 | 173 | 31.5 | | - | |  |  |  |
|  | 3 | 63 | 11.5 | | - | |  |  |  |
|  | 4 or more | 4 | 0.7 | | - | |  |  |  |
| >2-5% | 1 | 234 | 51.5 | | - | |  |  |  |
|  | 2 | 168 | 37.0 | | 1.29 | | 0.98 | 1.69 | 0.07 |
|  | 3 | 47 | 10.4 | | 0.99 | | 0.65 | 1.50 | 0.96 |
|  | 4 or more | 5 | 1.1 | | 1.66 | | 0.44 | 6.23 | 0.46 |
| >5-10% | 1 | 79 | 48.8 | | - | |  |  |  |
|  | **2** | 65 | 40.1 | | **1.47** | | 1.01 | 2.15 | 0.04 |
|  | 3 | 11 | 6.8 | | 0.69 | | 0.34 | 1.36 | 0.28 |
|  | **4 or more** | 7 | 4.3 | | **6.87** | | 1.96 | 24.05 | <0.01 |
| >10% | 1 | 42 | 43.8 | | - | |  |  |  |
|  | 2 | 35 | 36.5 | | 1.49 | | 0.92 | 2.43 | 0.11 |
|  | **3** | 16 | 16.7 | | **1.87** | | 0.99 | 3.54 | 0.05 |
|  | **4 or more** | 3 | 3.1 | | **5.54** | | 1.20 | 25.60 | 0.03 |
| Minimum locomotion score when farmer decided to treat lame sheep | | | | | | | |  |  |
| ≤2% | 1 | 164 | 29.9 | | Reference category | | |  |  |
|  | 2 | 220 | 40.1 | | - | |  |  |  |
|  | 3 | 126 | 23.0 | | - | |  |  |  |
|  | 4 | 26 | 4.7 | | - | |  |  |  |
|  | 5 | 4 | 0.7 | | - | |  |  |  |
|  | 6 | 0 | 0.0 | | - | |  |  |  |
|  | Individuals not treated | 8 | 1.5 | | - | |  |  |  |
| >2-5% | 1 | 110 | 24.3 | | - | |  |  |  |
|  | 2 | 190 | 42.0 | | 1.29 | | 0.94 | 1.76 | 0.11 |
|  | 3 | 117 | 25.9 | | 1.38 | | 0.98 | 1.96 | 0.07 |
|  | 4 | 26 | 5.8 | | 1.49 | | 0.82 | 2.70 | 0.19 |
|  | 5 | 2 | 0.4 | | 0.75 | | 0.13 | 4.14 | 0.74 |
|  | **6** | 1 | 0.2 | | **4.28E+05** | | 4.28E+05 | 4.28E+05 | <0.001 |
|  | Individuals not treated | 6 | 1.3 | | 1.12 | | 0.38 | 3.31 | 0.84 |
| >5-10% | 1 | 33 | 20.5 | | - | |  |  |  |
|  | 2 | 68 | 42.2 | | 1.54 | | 0.97 | 2.44 | 0.07 |
|  | **3** | 44 | 27.3 | | **1.74** | | 1.04 | 2.88 | 0.03 |
|  | **4** | 14 | 8.7 | | **2.68** | | 1.26 | 5.66 | 0.01 |
|  | 5 | 1 | 0.6 | | 1.24 | | 0.13 | 11.47 | 0.85 |
|  | **6** | 0 | 0.0 | | **0.22** | | 0.22 | 0.22 | <0.001 |
|  | Individuals not treated | 1 | 0.6 | | 0.62 | | 0.08 | 5.14 | 0.66 |
| >10% | 1 | 27 | 29.0 | | - | |  |  |  |
|  | 2 | 39 | 41.9 | | 1.08 | | 0.63 | 1.83 | 0.78 |
|  | 3 | 22 | 23.7 | | 1.06 | | 0.58 | 1.95 | 0.85 |
|  | 4 | 4 | 4.3 | | 0.93 | | 0.30 | 2.89 | 0.91 |
|  | 5 | 1 | 1.1 | | 1.52 | | 0.16 | 14.11 | 0.71 |
|  | **6** | 0 | 0.0 | | **0.27** | | 0.27 | 0.27 | <0.001 |
|  | Did not treat individuals | 0 | 0.0 | | **0.00** | | 0.00 | 0.00 | <0.001 |
| Number of sheep lame when treated at minimum locomotion score | | | | | | |  |  |  |
| ≤2% | 1 | 96 | 17.6 | | Reference category | | |  |  |
|  | 2-5 | 293 | 53.8 | | - | |  |  |  |
|  | 6-10 | 82 | 15.0 | | - | |  |  |  |
|  | >10 | 65 | 11.9 | | - | |  |  |  |
|  | Individuals not treated | 9 | 1.7 | | - | |  |  |  |
| >2-5% | 1 | 53 | 11.8 | | - | |  |  |  |
|  | **2-5** | 243 | 54.0 | | **1.50** | | 1.03 | 2.19 | 0.03 |
|  | **6-10** | 79 | 17.6 | | **1.75** | | 1.11 | 2.75 | 0.02 |
|  | **>10** | 71 | 15.8 | | **1.98** | | 1.23 | 3.18 | <0.001 |
|  | Individuals not treated | 4 | 0.9 | | 0.81 | | 0.24 | 2.74 | 0.73 |
| >5-10% | 1 | 17 | 10.6 | | - | |  |  |  |
|  | 2-5 | 72 | 45.0 | | 1.39 | | 0.78 | 2.47 | 0.27 |
|  | **6-10** | 39 | 24.4 | | **2.69** | | 1.41 | 5.10 | <0.001 |
|  | **>10** | 32 | 20.0 | | **2.78** | | 1.43 | 5.42 | <0.001 |
|  | Individuals not treated | 0 | 0.0 | | 0.00 | | 0.00 | 5.72E+292 | 0.97 |
| >10% | 1 | 9 | 9.5 | | - | |  |  |  |
|  | 2-5 | 40 | 42.1 | | 1.46 | | 0.68 | 3.11 | 0.33 |
|  | **6-10** | 23 | 24.2 | | **2.99** | | 1.31 | 6.83 | 0.01 |
|  | **>10** | 23 | 24.2 | | **3.77** | | 1.64 | 8.68 | <0.001 |
|  | **Individuals not treated** | 0 | 0.0 | | **0.00** | | 0.00 | 0.00 | <0.001 |
| Time to treat lame sheep from first day observed lame | | | | |  | |  |  |  |
| ≤2% | <1 day | 55 | 10.1 | | Reference category | | |  |  |
|  | 1-<3 days | 248 | 45.3 | | - | |  |  |  |
|  | >3-7 days | 183 | 33.5 | | - | |  |  |  |
|  | >7 days | 57 | 10.4 | | - | |  |  |  |
|  | Individuals not treated | 4 | 0.7 | | - | |  |  |  |
| >2-5% | <1 day | 27 | 6.0 | | - | |  |  |  |
|  | 1-3 days | 193 | 42.7 | | 1.59 | | 0.96 | 2.61 | 0.07 |
|  | **3-7 days** | 183 | 40.5 | | **2.04** | | 1.23 | 3.37 | 0.01 |
|  | >7 days | 48 | 10.6 | | 1.72 | | 0.94 | 3.12 | 0.08 |
|  | Individuals not treated | 1 | 0.2 | | 0.51 | | 0.05 | 4.78 | 0.55 |
| >5-10% | <1 day | 7 | 4.3 | | - | |  |  |  |
|  | 1-3 days | 60 | 37.0 | | 1.90 | | 0.82 | 4.38 | 0.13 |
|  | **3-7 days** | 73 | 45.1 | | **3.13** | | 1.36 | 7.20 | 0.01 |
|  | **>7 days** | 22 | 13.6 | | **3.03** | | 1.20 | 7.67 | 0.02 |
|  | **Individuals not treated** | 0 | 0.0 | | **0.00** | | 0.00 | 0.00 | <0.001 |
| >10% | <1 day | 2 | 2.1 | | - | |  |  |  |
|  | **1-3 days** | 41 | 43.2 | | **4.54** | | 1.07 | 19.33 | 0.04 |
|  | **3-7 days** | 41 | 43.2 | | **6.16** | | 1.44 | 26.25 | 0.01 |
|  | **>7 days** | 11 | 11.6 | | **5.30** | | 1.12 | 25.00 | 0.04 |
|  | Individuals not treated | 0 | 0.0 | | 0.00 | | 0.00 | 1.20E+214 | 0.97 |
| Use central handling facility to catch lame sheep | | | | |  | |  |  |  |
| ≤2% | No | 247 | 44.7 | | Reference category | | |  |  |
|  | Yes | 306 | 55.3 | | - | |  |  |  |
| >2-5% | No | 201 | 44.1 | | - | |  |  |  |
|  | Yes | 255 | 55.9 | | 1.02 | | 0.80 | 1.31 | 0.85 |
| >5-10% | No | 64 | 38.8 | | - | |  |  |  |
|  | Yes | 101 | 61.2 | | 1.27 | | 0.89 | 1.82 | 0.18 |
| >10% | No | 30 | 30.9 | | - | |  |  |  |
|  | **Yes** | 67 | 69.1 | | **1.80** | | 1.14 | 2.86 | 0.01 |
| Use dog that can catch flock to catch lame sheep | | | | |  | |  |  |  |
| ≤2% | No | 393 | 44.7 | | Reference category | | |  |  |
|  | Yes | 160 | 55.3 | | - | |  |  |  |
| >2-5% | No | 325 | 44.1 | | - | |  |  |  |
|  | Yes | 131 | 55.9 | | 0.99 | | 0.75 | 1.30 | 0.94 |
| >5-10% | No | 101 | 38.8 | | - | |  |  |  |
|  | **Yes** | 64 | 61.2 | | **1.56** | | 1.08 | 2.24 | 0.02 |
| >10% | No | 69 | 30.9 | | - | |  |  |  |
|  | Yes | 28 | 69.1 | | 1.00 | | 0.62 | 1.60 | 0.99 |
| Treat ewes with SFR with foot trim | |  |  | |  | |  |  |  |
| ≤2% | Always | 160 | 31.6 | | Reference category | | |  |  |
|  | Usually | 105 | 20.7 | | - | |  |  |  |
|  | Sometimes | 27 | 5.3 | | - | |  |  |  |
|  | Never | 215 | 42.4 | | - | |  |  |  |
| >2-5% | Always | 155 | 35.6 | | - | |  |  |  |
|  | Usually | 86 | 19.8 | | 1.14 | | 0.85 | 1.54 | 0.37 |
|  | Sometimes | 12 | 2.8 | | 0.97 | | 0.68 | 1.37 | 0.85 |
|  | Never | 182 | 41.8 | | 0.53 | | 0.26 | 1.07 | 0.07 |
| >5-10% | Always | 53 | 35.6 | | - | |  |  |  |
|  | Usually | 27 | 18.1 | | 1.06 | | 0.70 | 1.61 | 0.77 |
|  | Sometimes | 2 | 1.3 | | 0.83 | | 0.50 | 1.37 | 0.45 |
|  | **Never** | 67 | 45.0 | | **0.24** | | 0.06 | 1.03 | 0.05 |
| >10% | Always | 32 | 35.6 | | - | |  |  |  |
|  | Usually | 17 | 18.9 | | 1.05 | | 0.63 | 1.74 | 0.85 |
|  | Sometimes | 0 | 0.0 | | 0.85 | | 0.46 | 1.57 | 0.60 |
|  | Never | 41 | 45.6 | | 0.00 | | 0.00 | 2.18E+51 | 0.88 |
| Treat ewes with SFR with antibiotic injection | | |  | |  | |  |  |  |
| ≤2% | Always | 128 | 25.3 | | Reference category | | |  |  |
|  | Usually | 205 | 40.6 | | - | |  |  |  |
|  | Sometimes | 49 | 9.7 | | - | |  |  |  |
|  | Never | 123 | 24.4 | | - | |  |  |  |
| >2-5% | Always | 99 | 23.0 | | - | |  |  |  |
|  | Usually | 191 | 44.4 | | 0.85 | | 0.59 | 1.23 | 0.38 |
|  | Sometimes | 28 | 6.5 | | 1.02 | | 0.74 | 1.41 | 0.89 |
|  | Never | 112 | 26.0 | | 0.63 | | 0.37 | 1.07 | 0.09 |
| >5-10% | Always | 53 | 35.1 | | - | |  |  |  |
|  | **Usually** | 61 | 40.4 | | **1.70** | | 1.02 | 2.83 | 0.04 |
|  | Sometimes | 7 | 4.6 | | 1.22 | | 0.75 | 1.99 | 0.43 |
|  | Never | 30 | 19.9 | | 0.59 | | 0.24 | 1.42 | 0.24 |
| >10% | Always | 29 | 31.5 | | - | |  |  |  |
|  | Usually | 38 | 41.3 | | 1.27 | | 0.69 | 2.32 | 0.45 |
|  | Sometimes | 3 | 3.3 | | 1.04 | | 0.59 | 1.83 | 0.90 |
|  | Never | 22 | 23.9 | | 0.34 | | 0.10 | 1.20 | 0.09 |
| Treat ewes with SFR with antibiotic injection | | |  | |  | |  |  |  |
| ≤2% | Always | 123 | 24.1 | | Reference category | | |  |  |
|  | Usually | 55 | 10.8 | | - | |  |  |  |
|  | Sometimes | 19 | 3.7 | | - | |  |  |  |
|  | Never | 314 | 61.4 | | - | |  |  |  |
| >2-5% | Always | 90 | 20.5 | | - | |  |  |  |
|  | Usually | 35 | 8.0 | | 0.75 | | 0.55 | 1.02 | 0.07 |
|  | Sometimes | 7 | 1.6 | | 0.65 | | 0.41 | 1.02 | 0.06 |
|  | **Never** | 307 | 69.9 | | **0.38** | | 0.16 | 0.91 | 0.03 |
| >5-10% | Always | 34 | 22.4 | | - | |  |  |  |
|  | Usually | 16 | 10.5 | | 0.87 | | 0.56 | 1.35 | 0.53 |
|  | Sometimes | 2 | 1.3 | | 0.91 | | 0.50 | 1.67 | 0.77 |
|  | Never | 100 | 65.8 | | 0.33 | | 0.08 | 1.44 | 0.14 |
| >10% | Always | 24 | 25.5 | | - | |  |  |  |
|  | Usually | 12 | 12.8 | | 1.07 | | 0.64 | 1.81 | 0.79 |
|  | Sometimes | 1 | 1.1 | | 1.20 | | 0.61 | 2.38 | 0.60 |
|  | Never | 57 | 60.6 | | 0.29 | | 0.04 | 2.21 | 0.23 |
| Treat lambs with SFR with foot trim | |  |  | |  | |  |  |  |
| ≤2% | Always | 87 | 19.2 | | Reference category | | |  |  |
|  | Usually | 191 | 42.1 | | - | |  |  |  |
|  | Sometimes | 110 | 24.2 | | - | |  |  |  |
|  | Never | 66 | 14.5 | | - | |  |  |  |
| >2-5% | Always | 87 | 21.9 | | - | |  |  |  |
|  | Usually | 185 | 46.5 | | 0.88 | | 0.56 | 1.37 | 0.57 |
|  | Sometimes | 51 | 12.8 | | 0.85 | | 0.58 | 1.26 | 0.42 |
|  | **Never** | 75 | 18.8 | | **0.41** | | 0.26 | 0.65 | <0.001 |
| >5-10% | Always | 29 | 20.3 | | - | |  |  |  |
|  | Usually | 70 | 49.0 | | 0.63 | | 0.35 | 1.13 | 0.12 |
|  | Sometimes | 9 | 6.3 | | 0.69 | | 0.42 | 1.13 | 0.14 |
|  | **Never** | 35 | 24.5 | | 0.15 | | 0.07 | 0.34 | <0.001 |
| >10% | Always | 19 | 21.8 | | - | |  |  |  |
|  | Usually | 48 | 55.2 | | 0.85 | | 0.41 | 1.76 | 0.66 |
|  | Sometimes | 3 | 3.4 | | 0.98 | | 0.52 | 1.81 | 0.94 |
|  | **Never** | 17 | 19.5 | | **0.11** | | 0.03 | 0.38 | <0.001 |
| Treat lambs with SFR with antibiotic injection | | |  | |  | |  |  |  |
| ≤2% | Always | 61 | 13.6 | | Reference category | | |  |  |
|  | Usually | 206 | 45.9 | | - | |  |  |  |
|  | Sometimes | 122 | 27.2 | | - | |  |  |  |
|  | Never | 60 | 13.4 | | - | |  |  |  |
| >2-5% | Always | 64 | 16.0 | | - | |  |  |  |
|  | Usually | 181 | 45.1 | | 0.95 | | 0.58 | 1.57 | 0.85 |
|  | Sometimes | 90 | 22.4 | | 0.80 | | 0.53 | 1.19 | 0.27 |
|  | Never | 66 | 16.5 | | 0.67 | | 0.43 | 1.04 | 0.08 |
| >5-10% | Always | 19 | 13.1 | | - | |  |  |  |
|  | Usually | 83 | 57.2 | | 0.89 | | 0.44 | 1.82 | 0.75 |
|  | Sometimes | 22 | 15.2 | | 1.15 | | 0.66 | 2.01 | 0.62 |
|  | **Never** | 21 | 14.5 | | **0.52** | | 0.26 | 1.01 | 0.05 |
| >10% | Always | 11 | 12.9 | | - | |  |  |  |
|  | Usually | 44 | 51.8 | | 0.64 | | 0.28 | 1.47 | 0.29 |
|  | Sometimes | 13 | 15.3 | | 0.75 | | 0.40 | 1.41 | 0.38 |
|  | **Never** | 17 | 20.0 | | 0.38 | | 0.17 | 0.82 | 0.01 |
| Treat lambs with SFR with foot spray | | |  | |  | |  |  |  |
| ≤2% | Always | 114 | 24.8 | | Reference category | | |  |  |
|  | Usually | 80 | 17.4 | | - | |  |  |  |
|  | Sometimes | 28 | 6.1 | | - | |  |  |  |
|  | Never | 237 | 51.6 | | - | |  |  |  |
| >2-5% | Always | 85 | 21.0 | | - | |  |  |  |
|  | **Usually** | 54 | 13.3 | | **0.69** | | 0.49 | 0.96 | 0.03 |
|  | **Sometimes** | 9 | 2.2 | | **0.62** | | 0.42 | 0.92 | 0.02 |
|  | **Never** | 257 | 63.5 | | **0.30** | | 0.14 | 0.64 | <0.001 |
| >5-10% | Always | 41 | 28.3 | | - | |  |  |  |
|  | Usually | 15 | 10.3 | | 0.98 | | 0.64 | 1.51 | 0.93 |
|  | **Sometimes** | 2 | 1.4 | | **0.51** | | 0.28 | 0.93 | 0.03 |
|  | **Never** | 87 | 60.0 | | **0.19** | | 0.05 | 0.83 | 0.03 |
| >10% | Always | 22 | 25.9 | | - | |  |  |  |
|  | Usually | 8 | 9.4 | | 0.85 | | 0.49 | 1.46 | 0.55 |
|  | **Sometimes** | 1 | 1.2 | | **0.44** | | 0.20 | 0.96 | 0.04 |
|  | Never | 54 | 63.5 | | 0.16 | | 0.02 | 1.18 | 0.07 |
| Treat ewes with ID with foot trim | |  |  | |  | |  |  |  |
| ≤2% | Always | 69 | 15.5 | | Reference category | | |  |  |
|  | Usually | 201 | 45.2 | | - | |  |  |  |
|  | Sometimes | 124 | 27.9 | | - | |  |  |  |
|  | Never | 51 | 11.5 | | - | |  |  |  |
| >2-5% | Always | 66 | 17.3 | | - | |  |  |  |
|  | Usually | 172 | 45.1 | | 1.19 | | 0.70 | 2.03 | 0.52 |
|  | Sometimes | 102 | 26.8 | | 1.06 | | 0.67 | 1.68 | 0.79 |
|  | Never | 41 | 10.8 | | 1.02 | | 0.63 | 1.67 | 0.93 |
| >5-10% | Always | 26 | 18.3 | | - | |  |  |  |
|  | Usually | 61 | 43.0 | | 1.01 | | 0.51 | 2.02 | 0.97 |
|  | Sometimes | 36 | 25.4 | | 0.81 | | 0.45 | 1.48 | 0.50 |
|  | Never | 19 | 13.4 | | 0.78 | | 0.41 | 1.48 | 0.45 |
| >10% | Always | 18 | 22.2 | | - | |  |  |  |
|  | Usually | 41 | 50.6 | | 1.21 | | 0.53 | 2.78 | 0.65 |
|  | Sometimes | 11 | 13.6 | | 0.95 | | 0.45 | 1.97 | 0.88 |
|  | **Never** | 11 | 13.6 | | **0.41** | | 0.17 | 1.01 | 0.05 |
| Treat ewes with ID with foot spray | |  |  | |  | |  |  |  |
| ≤2% | Always | 117 | 24.8 | | Reference category | | |  |  |
|  | Usually | 56 | 11.9 | | - | |  |  |  |
|  | Sometimes | 24 | 5.1 | | - | |  |  |  |
|  | Never | 275 | 58.3 | | - | |  |  |  |
| >2-5% | Always | 88 | 22.3 | | - | |  |  |  |
|  | Usually | 44 | 11.1 | | 0.80 | | 0.58 | 1.11 | 0.19 |
|  | Sometimes | 6 | 1.5 | | 0.84 | | 0.55 | 1.29 | 0.43 |
|  | **Never** | 257 | 65.1 | | **0.27** | | 0.11 | 0.67 | <0.001 |
| >5-10% | Always | 34 | 23.4 | | - | |  |  |  |
|  | Usually | 13 | 9.0 | | 0.84 | | 0.54 | 1.32 | 0.45 |
|  | Sometimes | 3 | 2.1 | | 0.67 | | 0.35 | 1.28 | 0.23 |
|  | Never | 95 | 65.5 | | 0.36 | | 0.11 | 1.23 | 0.10 |
| >10% | Always | 23 | 26.7 | | - | |  |  |  |
|  | Usually | 12 | 14.0 | | 1.13 | | 0.65 | 1.94 | 0.67 |
|  | Sometimes | 3 | 3.5 | | 1.23 | | 0.61 | 2.46 | 0.56 |
|  | Never | 48 | 55.8 | | 0.72 | | 0.21 | 2.47 | 0.60 |
| Treat lambs with ID with foot trim | |  |  | |  | |  |  |  |
| ≤2% | Always | 37 | 8.6 | | Reference category | | |  |  |
|  | Usually | 198 | 45.8 | | - | |  |  |  |
|  | Sometimes | 177 | 41.0 | | - | |  |  |  |
|  | Never | 20 | 4.6 | | - | |  |  |  |
| >2-5% | Always | 42 | 11.1 | | - | |  |  |  |
|  | Usually | 177 | 46.7 | | 0.78 | | 0.38 | 1.61 | 0.51 |
|  | Sometimes | 131 | 34.6 | | 0.62 | | 0.34 | 1.13 | 0.12 |
|  | **Never** | 29 | 7.7 | | **0.51** | | 0.28 | 0.94 | 0.03 |
| >5-10% | Always | 13 | 9.1 | | - | |  |  |  |
|  | **Usually** | 65 | 45.5 | | **0.39** | | 0.16 | 0.96 | 0.04 |
|  | **Sometimes** | 47 | 32.9 | | **0.36** | | 0.18 | 0.73 | <0.001 |
|  | **Never** | 18 | 12.6 | | **0.30** | | 0.14 | 0.60 | <0.001 |
| >10% | Always | 9 | 11.3 | | - | |  |  |  |
|  | Usually | 44 | 55.0 | | 0.54 | | 0.19 | 1.58 | 0.26 |
|  | Sometimes | 18 | 22.5 | | 0.49 | | 0.21 | 1.16 | 0.10 |
|  | **Never** | 9 | 11.3 | | **0.23** | | 0.09 | 0.57 | <0.001 |
| Treat lambs with ID with antibiotic injection | | |  | |  | |  |  |  |
| ≤2% | Always | 33 | 7.8 | | Reference category | | |  |  |
|  | Usually | 151 | 35.5 | | - | |  |  |  |
|  | Sometimes | 226 | 53.2 | | - | |  |  |  |
|  | Never | 15 | 3.5 | | - | |  |  |  |
| >2-5% | Always | 31 | 8.0 | | - | |  |  |  |
|  | Usually | 131 | 33.9 | | 0.74 | | 0.32 | 1.71 | 0.48 |
|  | Sometimes | 205 | 53.1 | | 0.68 | | 0.33 | 1.40 | 0.30 |
|  | Never | 19 | 4.9 | | 0.72 | | 0.35 | 1.45 | 0.35 |
| >5-10% | Always | 8 | 5.5 | | - | |  |  |  |
|  | Usually | 56 | 38.6 | | 0.61 | | 0.18 | 2.06 | 0.42 |
|  | Sometimes | 75 | 51.7 | | 0.93 | | 0.34 | 2.51 | 0.88 |
|  | Never | 6 | 4.1 | | 0.83 | | 0.31 | 2.22 | 0.71 |
| >10% | Always | 3 | 3.9 | | - | |  |  |  |
|  | Usually | 36 | 47.4 | | 0.23 | | 0.05 | 1.03 | 0.06 |
|  | Sometimes | 31 | 40.8 | | 0.60 | | 0.22 | 1.64 | 0.32 |
|  | **Never** | 6 | 7.9 | | **0.34** | | 0.12 | 0.95 | 0.04 |
| Treat lambs with ID with foot spray | |  |  | |  | |  |  |  |
| ≤2% | Always | 100 | 21.8 | | Reference category | | |  |  |
|  | Usually | 83 | 18.1 | | - | |  |  |  |
|  | Sometimes | 26 | 5.7 | | - | |  |  |  |
|  | Never | 250 | 54.5 | | - | |  |  |  |
| >2-5% | Always | 84 | 21.3 | | - | |  |  |  |
|  | Usually | 57 | 14.5 | | 0.85 | | 0.60 | 1.19 | 0.34 |
|  | Sometimes | 5 | 1.3 | | 0.69 | | 0.47 | 1.01 | 0.06 |
|  | **Never** | 248 | 62.9 | | **0.19** | | 0.07 | 0.51 | <0.001 |
| >5-10% | Always | 40 | 26.1 | | - | |  |  |  |
|  | Usually | 14 | 9.2 | | 1.04 | | 0.67 | 1.61 | 0.85 |
|  | **Sometimes** | 3 | 2.0 | | **0.44** | | 0.24 | 0.81 | 0.01 |
|  | **Never** | 96 | 62.7 | | **0.30** | | 0.09 | 1.02 | 0.05 |
| >10% | Always | 18 | 22.0 | | - | |  |  |  |
|  | Usually | 11 | 13.4 | | 0.85 | | 0.47 | 1.52 | 0.58 |
|  | Sometimes | 0 | 0.0 | | 0.63 | | 0.31 | 1.25 | 0.19 |
|  | Never | 53 | 64.6 | | 0.00 | | 0.00 | 2.20E+37 | 0.85 |
| Use of Lincospectin foot spray | |  |  | |  | |  |  |  |
| ≤2% | No | 510 | 92.2 | | Reference category | | |  |  |
|  | Yes | 43 | 7.8 | | - | |  |  |  |
| 2-5% | No | 406 | 89.0 | | - | |  |  |  |
|  | Yes | 50 | 11.0 | | 1.46 | | 0.95 | 2.24 | 0.08 |
| >5-10% | No | 144 | 87.3 | | - | |  |  |  |
|  | **Yes** | 21 | 12.7 | | **1.73** | | 0.99 | 3.01 | 0.05 |
| >10% | No | 79 | 81.4 | | - | |  |  |  |
|  | **Yes** | 18 | 18.6 | | **2.70** | | 1.48 | 4.92 | <0.001 |
| Use of antibiotic aerosol foot spray | |  |  | |  | |  |  |  |
| <2% | No | 91 | 16.5 | | Reference category | | |  |  |
|  | Yes | 462 | 83.5 | | - | |  |  |  |
| >2-5% | No | 56 | 12.3 | | - | |  |  |  |
|  | Yes | 400 | 87.7 | | 1.41 | | 0.98 | 2.01 | 0.06 |
| >5-10% | No | 19 | 11.5 | | - | |  |  |  |
|  | Yes | 146 | 88.5 | | 1.51 | | 0.89 | 2.57 | 0.12 |
| >10% | No | 7 | 7.2 | | - | |  |  |  |
|  | **Yes** | 90 | 92.8 | | **2.53** | | 1.14 | 5.64 | 0.02 |
| Routine foot trim the flock | |  |  | | | |  |  |  |
| <2% | Did not trim | 243 | | 46.2 | | Reference category | |  |  |
|  | Trimmed but no bleeding | 46 | 8.2 | | - | |  |  |  |
|  | Caused bleeding | 237 | 45.1 | | - | |  |  |  |
| >2-5% | Did not trim | 183 | 42.3 | | - | |  |  |  |
|  | Trimmed but no bleeding | 24 | 5.5 | | 0.69 | | 0.41 | 1.18 | 0.17 |
|  | Caused bleeding | 226 | 52.2 | | 1.27 | | 0.97 | 1.65 | 0.08 |
| >5-10% | Did not trim | 74 | 48.4 | | - | |  |  |  |
|  | Trimmed but no bleeding | 7 | 4.6 | | 0.50 | | 0.22 | 1.15 | 0.10 |
|  | Caused bleeding | 72 | 47.1 | | 1.00 | | 0.69 | 1.45 | 0.99 |
| >10% | Did not trim | 25 | 25.5 | | - | |  |  |  |
|  | Trimmed but no bleeding | 5 | 5.3 | | 1.10 | | 0.4 | 3.03 | 0.85 |
|  | **Caused bleeding** | 65 | 69.2 | | **2.78** | | 1.68 | 4.58 | <0.001 |
| Footbath of ewes | |  |  | |  | |  |  |  |
| ≤2% | No | 257 | 46.5 | | Reference category | | |  |  |
|  | Yes | 296 | 53.5 | | - | |  |  |  |
| >2-5% | No | 170 | 62.7 | | - | |  |  |  |
|  | **Yes** | 286 | 12.1 | | **1.46** | | 1.13 | 1.88 | <0.001 |
| >5-10% | No | 55 | 33.3 | | - | |  |  |  |
|  | **Yes** | 110 | 66.7 | | **1.74** | | 1.21 | 2.50 | <0.001 |
| >10% | No | 27 | 27.8 | | - | |  |  |  |
|  | Yes | 70 | 72.2 | | **2.25** | | 1.40 | 3.62 | <0.001 |
| Footbath of lambs | |  |  | |  | |  |  |  |
| <2% | No | 290 | 52.4 | | Reference category | | |  |  |
|  | Yes | 263 | 47.6 | | - | |  |  |  |
| >2-5% | No | 179 | 60.7 | | - | |  |  |  |
|  | **Yes** | 277 | 12.3 | | **1.71** | | 1.33 | 2.19 | <0.001 |
| >5-10% | No | 56 | 33.9 | | - | |  |  |  |
|  | **Yes** | 109 | 66.1 | | **2.15** | | 1.49 | 3.09 | <0.001 |
| >10% | No | 28 | 28.9 | | - | |  |  |  |
|  | **Yes** | 69 | 71.1 | | **2.72** | | 1.70 | 4.35 | <0.001 |
| Did not footbath | |  |  | |  | |  |  |  |
| ≤2% | No | 318 | 57.5 | | Reference category | | |  |  |
|  | Yes | 235 | 42.5 | | - | |  |  |  |
| >2-5% | No | 305 | 33.1 | | - | |  |  |  |
|  | **Yes** | 151 | 25.9 | | **0.67** | | 0.52 | 0.87 | <0.001 |
| >5-10% | No | 118 | 71.5 | | - | |  |  |  |
|  | **Yes** | 47 | 28.5 | | **0.54** | | 0.37 | 0.79 | <0.001 |
| >10% | No | 74 | 76.3 | | - | |  |  |  |
|  | **Yes** | 23 | 23.7 | | **0.42** | | 0.26 | 0.69 | <0.001 |
| Footbath to treat ID | |  |  | |  | |  |  |  |
| ≤2% | No | 326 | 59.0 | | Reference category | | |  |  |
|  | Yes | 227 | 41.0 | | - | |  |  |  |
| >2-5% | No | 230 | 49.6 | | - | |  |  |  |
|  | **Yes** | 226 | 12.7 | | **1.41** | | 1.10 | 1.81 | 0.01 |
| >5-10% | No | 58 | 35.2 | | - | |  |  |  |
|  | **Yes** | 107 | 64.8 | | **2.65** | | 1.85 | 3.80 | <0.001 |
| >10% | No | 37 | 38.1 | | - | |  |  |  |
|  | **Yes** | 60 | 61.9 | | **2.33** | | 1.49 | 3.63 | <0.001 |
| Footbath to treat SFR | |  |  | |  | |  |  |  |
| ≤2% | No | 385 | 69.6 | | Reference category | | |  |  |
|  | Yes | 168 | 30.4 | | - | |  |  |  |
| >2-5% | No | 285 | 37.5 | | - | |  |  |  |
|  | **Yes** | 171 | 21.9 | | **1.37** | | 1.06 | 1.79 | 0.02 |
| >5-10% | No | 100 | 60.6 | | - | |  |  |  |
|  | **Yes** | 65 | 39.4 | | **1.49** | | 1.04 | 2.14 | 0.03 |
| >10% | No | 44 | 45.4 | |  | |  |  |  |
|  | **Yes** | 53 | 54.6 | | **2.76** | | 1.78 | 4.28 | <0.001 |
| Footbath to prevent ID | |  |  | |  | |  |  |  |
| ≤2% | No | 367 | 66.4 | | Reference category | | |  |  |
|  | Yes | 186 | 33.6 | | - | |  |  |  |
| >2-5% | No | 286 | 37.3 | | - | |  |  |  |
|  | Yes | 170 | 22.1 | | 1.17 | | 0.91 | 1.52 | 0.23 |
| >5-10% | No | 101 | 61.2 | | - | |  |  |  |
|  | Yes | 64 | 38.8 | | 1.25 | | 0.87 | 1.79 | 0.22 |
| >10% | No | 64 | 66.0 | | - | |  |  |  |
|  | Yes | 33 | 34.0 | | 1.02 | | 0.65 | 1.60 | 0.94 |
| Footbath to prevent SFR | |  |  | |  | |  |  |  |
| ≤2% | No | 360 | 65.1 | | Reference category | | |  |  |
|  | Yes | 193 | 34.9 | | - | |  |  |  |
| >2-5% | No | 291 | 36.2 | | - | |  |  |  |
|  | Yes | 165 | 23.9 | | 1.06 | | 0.82 | 1.37 | 0.67 |
| >5-10% | No | 109 | 66.1 | | - | |  |  |  |
|  | Yes | 56 | 33.9 | | 0.96 | | 0.66 | 1.38 | 0.82 |
| >10% | No | 59 | 60.8 | | - | |  |  |  |
|  | Yes | 38 | 39.2 | | 1.20 | | 0.77 | 1.87 | 0.42 |
| Footbath lambs before housing | |  |  | |  | |  |  |  |
| ≤2% | No | 519 | 93.9 | | Reference category | | |  |  |
|  | Yes | 34 | 6.1 | | - | |  |  |  |
| >2-5% | No | 427 | 6.4 | | - | |  |  |  |
|  | Yes | 29 | 32.0 | | 1.04 | | 0.62 | 1.73 | 0.89 |
| >5-10% | No | 146 | 88.5 | | - | |  |  |  |
|  | **Yes** | 19 | 11.5 | | **1.99** | | 1.10 | 3.59 | 0.02 |
| >10% | No | 87 | 89.7 | | - | |  |  |  |
|  | Yes | 10 | 10.3 | | 1.76 | | 0.84 | 3.68 | 0.14 |
| Footbath lambs when moving field | |  |  | |  | |  |  |  |
| ≤2% | No | 456 | 82.5 | | Reference category | | |  |  |
|  | Yes | 97 | 17.5 | | - | |  |  |  |
| >2-5% | No | 360 | 21.1 | | - | |  |  |  |
|  | Yes | 96 | 27.2 | | 1.25 | | 0.92 | 1.72 | 0.16 |
| >5-10% | No | 124 | 75.2 | | - | |  |  |  |
|  | **Yes** | 41 | 24.8 | | **1.55** | | 1.03 | 2.36 | 0.04 |
| >10% | No | 70 | 72.2 | | - | |  |  |  |
|  | **Yes** | 27 | 27.8 | | **1.81** | | 1.11 | 2.98 | 0.02 |
| Footbath lambs after gathering | |  |  | |  | |  |  |  |
| ≤2% | No | 414 | 74.9 | | Reference category | | |  |  |
|  | Yes | 139 | 25.1 | | - | |  |  |  |
| >2-5% | No | 328 | 28.1 | | - | |  |  |  |
|  | Yes | 128 | 21.5 | | 1.16 | | 0.88 | 1.54 | 0.29 |
| >5-10% | No | 98 | 59.4 | | - | |  |  |  |
|  | **Yes** | 67 | 40.6 | | **2.04** | | 1.41 | 2.93 | <0.001 |
| >10% | No | 54 | 55.7 | | - | |  |  |  |
|  | **Yes** | 43 | 44.3 | | **2.37** | | 1.52 | 3.70 | <0.001 |
| Footbath lambs when sheep return to farm | | |  | |  | |  |  |  |
| ≤2% | No | 520 | 94.0 | | Reference category | | |  |  |
|  | Yes | 33 | 6.0 | | - | |  |  |  |
| >2-5% | No | 431 | 5.5 | | - | |  |  |  |
|  | Yes | 25 | 32.5 | | 0.91 | | 0.54 | 1.56 | 0.74 |
| >5-10% | No | 148 | 89.7 | | - | |  |  |  |
|  | Yes | 17 | 10.3 | | 1.81 | | 0.98 | 3.34 | 0.06 |
| >10% | No | 85 | 87.6 | | - | |  |  |  |
|  | **Yes** | 12 | 12.4 | | **2.22** | | 1.11 | 4.48 | 0.03 |
| Footbath ewes when moving field | |  |  | |  | |  |  |  |
| ≤2% | No | 457 | 82.6 | | Reference category | | |  |  |
|  | Yes | 96 | 17.4 | | - | |  |  |  |
| >2-5% | No | 366 | 19.7 | | - | |  |  |  |
|  | Yes | 90 | 27.6 | | 1.17 | | 0.85 | 1.61 | 0.33 |
| >5-10% | No | 126 | 76.4 | | - | |  |  |  |
|  | Yes | 39 | 23.6 | | 1.47 | | 0.97 | 2.25 | 0.07 |
| >10% | No | 67 | 69.1 | | - | |  |  |  |
|  | **Yes** | 30 | 30.9 | | **2.13** | | 1.31 | 3.46 | <0.001 |
| Footbath ewes after gathering | |  |  | |  | |  |  |  |
| ≤2% | No | 417 | 75.4 | | Reference category | | |  |  |
|  | Yes | 136 | 24.6 | | - | |  |  |  |
| >2-5% | No | 315 | 30.9 | | - | |  |  |  |
|  | **Yes** | 141 | 22.1 | | **1.37** | | 1.04 | 1.81 | 0.03 |
| >5-10% | No | 101 | 61.2 | | - | |  |  |  |
|  | **Yes** | 64 | 38.8 | | **1.94** | | 1.34 | 2.81 | <0.001 |
| >10% | No | 58 | 59.8 | | - | |  |  |  |
|  | **Yes** | 39 | 40.2 | | **2.06** | | 1.32 | 3.23 | <0.001 |
| Footbath ewes when sheep return to farm | | |  | |  | |  |  |  |
| ≤2% | No | 508 | 91.9 | | Reference category | | |  |  |
|  | Yes | 45 | 8.1 | | - | |  |  |  |
| >2-5% | No | 418 | 8.3 | | - | |  |  |  |
|  | Yes | 38 | 31.4 | | 1.03 | | 0.65 | 1.61 | 0.91 |
| >5-10% | No | 143 | 86.7 | | - | |  |  |  |
|  | **Yes** | 22 | 13.3 | | **1.74** | | 1.01 | 2.99 | 0.05 |
| >10% | No | 84 | 86.6 | | - | |  |  |  |
|  | Yes | 13 | 13.4 | | 1.75 | | 0.90 | 3.38 | 0.10 |
| Routine footbathing of lambs at pasture | | |  | |  | |  |  |  |
| ≤2% | Once a week | 3 | 0.9 | | Reference category | | |  |  |
|  | Once a fortnight | 36 | 10.9 | | - | |  |  |  |
|  | Once a month | 67 | 20.2 | | - | |  |  |  |
|  | Other | 74 | 22.4 | | - | |  |  |  |
|  | Did not do routinely | 151 | 45.6 | | - | |  |  |  |
| >2-5% | Once a week | 7 | 2.3 | | - | |  |  |  |
|  | Once a fortnight | 35 | 11.4 | | 0.42 | | 0.10 | 1.74 | 0.23 |
|  | Once a month | 87 | 28.3 | | 0.56 | | 0.14 | 2.23 | 0.41 |
|  | Other | 65 | 21.2 | | 0.38 | | 0.09 | 1.52 | 0.17 |
|  | Did not do routinely | 113 | 36.8 | | 0.32 | | 0.08 | 1.27 | 0.10 |
| >5-10% | Once a week | 6 | 5.0 | | - | |  |  |  |
|  | **Once a fortnight** | 14 | 11.8 | | **0.19** | | 0.04 | 0.89 | 0.03 |
|  | Once a month | 33 | 27.7 | | 0.25 | | 0.06 | 1.05 | 0.06 |
|  | **Other** | 25 | 21.0 | | **0.17** | | 0.04 | 0.73 | 0.02 |
|  | **Did not do routinely** | 41 | 34.5 | | **0.14** | | 0.03 | 0.57 | 0.01 |
| >10% | Once a week | 2 | 2.6 | | - | |  |  |  |
|  | Once a fortnight | 11 | 14.5 | | 0.46 | | 0.07 | 3.10 | 0.42 |
|  | Once a month | 24 | 31.6 | | 0.54 | | 0.08 | 3.41 | 0.51 |
|  | Other | 20 | 26.3 | | 0.41 | | 0.06 | 2.59 | 0.34 |
|  | Did not do routinely | 19 | 25.0 | | 0.19 | | 0.03 | 1.20 | 0.08 |
| Routine footbathing of housed ewes | |  |  | |  | |  |  |  |
| ≤2% | Once a week | 20 | 6.6 | | Reference category | | |  |  |
|  | Once a fortnight | 21 | 6.9 | | - | |  |  |  |
|  | Once a month | 32 | 10.5 | | - | |  |  |  |
|  | Other | 1 | 0.3 | | - | |  |  |  |
|  | No routine footbathing | 158 | 51.8 | | - | |  |  |  |
|  | Did not house ewes | 73 | 23.9 | | - | |  |  |  |
| >2-5% | Once a week | 9 | 3.0 | | - | |  |  |  |
|  | Once a fortnight | 21 | 7.1 | | 2.22 | | 0.82 | 5.99 | 0.11 |
|  | **Once a month** | 36 | 12.2 | | **2.50** | | 1.00 | 6.27 | 0.05 |
|  | Other | 2 | 0.7 | | 4.44 | | 0.36 | 55.58 | 0.25 |
|  | No routine footbathing | 144 | 48.6 | | 2.03 | | 0.89 | 4.59 | 0.09 |
|  | **Did not house ewes** | 84 | 28.4 | | **2.56** | | 1.10 | 5.96 | 0.03 |
| >5-10% | Once a week | 6 | 5.4 | | - | |  |  |  |
|  | Once a fortnight | 12 | 10.7 | | 1.90 | | 0.60 | 6.05 | 0.27 |
|  | Once a month | 16 | 14.3 | | 1.67 | | 0.56 | 4.97 | 0.36 |
|  | Other | 1 | 0.9 | | 3.33 | | 0.18 | 61.69 | 0.42 |
|  | No routine footbathing | 51 | 45.5 | | 1.08 | | 0.41 | 2.83 | 0.88 |
|  | Did not house ewes | 26 | 23.2 | | 1.19 | | 0.43 | 3.28 | 0.74 |
| >10% | Once a week | 2 | 2.9 | | - | |  |  |  |
|  | Once a fortnight | 3 | 4.4 | | 1.43 | | 0.22 | 9.47 | 0.71 |
|  | Once a month | 10 | 14.7 | | 3.12 | | 0.62 | 15.75 | 0.17 |
|  | Other | 1 | 1.5 | | 10.00 | | 0.44 | 228.70 | 0.15 |
|  | No routine footbathing | 34 | 50.0 | | 2.15 | | 0.48 | 9.64 | 0.32 |
|  | Did not house ewes | 18 | 26.5 | | 2.47 | | 0.53 | 11.53 | 0.25 |
| Number of times a sheep was lame before culling | | | | |  | |  |  |  |
| ≤2% | Did not cull when lame | 277 | 56.3 | | Reference category | | |  |  |
|  | Lame once | 25 | 5.1 | | - | |  |  |  |
|  | Lame twice | 70 | 14.2 | | - | |  |  |  |
|  | Lame >2 times | 104 | 21.1 | | - | |  |  |  |
|  | If persistently lame | 16 | 3.3 | | - | |  |  |  |
| >2-5% | Did not cull when lame | 212 | 52.1 | | - | |  |  |  |
|  | Lame once | 9 | 2.2 | | 0.47 | | 0.22 | 1.03 | 0.06 |
|  | Lame twice | 45 | 11.1 | | 0.84 | | 0.55 | 1.27 | 0.41 |
|  | **Lame >2 times** | 113 | 27.8 | | **1.42** | | 1.03 | 1.96 | 0.03 |
|  | If persistently lame | 28 | 6.9 | | **2.29** | | 1.21 | 4.34 | 0.01 |
| >5-10% | Did not cull when lame | 70 | 45.2 | | - | |  |  |  |
|  | Lame once | 4 | 2.6 | | 0.63 | | 0.21 | 1.88 | 0.41 |
|  | Lame twice | 19 | 12.3 | | 1.07 | | 0.61 | 1.90 | 0.81 |
|  | **Lame >2 times** | 56 | 36.1 | | **2.13** | | 1.40 | 3.23 | <0.001 |
|  | If persistently lame | 6 | 3.9 | | 1.48 | | 0.56 | 3.93 | 0.43 |
| >10% | Did not cull when lame | 38 | 46.9 | | - | |  |  |  |
|  | Lame once | 0 | 0.0 | | 0.00 | | 0.00 | 3.15E+109 | 0.93 |
|  | Lame twice | 9 | 11.1 | | 0.94 | | 0.43 | 2.03 | 0.87 |
|  | **Lame >2 times** | 27 | 33.3 | | **1.89** | | 1.10 | 3.25 | 0.02 |
|  | **If persistently lame** | 7 | 8.6 | | **3.19** | | 1.23 | 8.25 | 0.02 |
| Use EID ear tag to identify sheep for culling | | |  | |  | |  |  |  |
| ≤2% | No | 516 | 93.3 | | Reference category | | |  |  |
|  | Yes | 37 | 6.7 | | - | |  |  |  |
| >2-5% | No | 440 | 96.5 | | - | |  |  |  |
|  | Yes | 16 | 3.5 | | **0.51** | | 0.28 | 0.92 | 0.03 |
| >5-10% | No | 153 | 92.7 | | - | |  |  |  |
|  | Yes | 12 | 7.3 | | 1.09 | | 0.56 | 2.15 | 0.79 |
| >10% | No | 94 | 96.9 | | - | |  |  |  |
|  | Yes | 3 | 3.1 | | 0.45 | | 0.13 | 1.47 | 0.19 |
| Vaccinate of sheep with footrot | |  |  | |  | |  |  |  |
| ≤2% | No | 542 | 98.0 | | Reference category | | |  |  |
|  | Yes | 11 | 2.0 | | - | |  |  |  |
| 2-5% | No | 439 | 96.3 | | - | |  |  |  |
|  | Yes | 17 | 3.7 | | 1.91 | | 0.88 | 4.12 | 0.10 |
| 5-10% | No | 160 | 97.0 | | - | |  |  |  |
|  | Yes | 5 | 3.0 | | 1.54 | | 0.53 | 4.50 | 0.43 |
| >10% | No | 93 | 95.9 | | - | |  |  |  |
|  | Yes | 4 | 4.1 | | 2.12 | | 0.66 | 6.80 | 0.21 |
| Use oxytetracyline to treat the whole flock | | |  | |  | |  |  |  |
| ≤2% | No | 519 | 93.9 | | Reference category | | |  |  |
|  | Yes | 34 | 6.2 | | - | |  |  |  |
| 2-5% | No | 421 | 92.3 | | - | |  |  |  |
|  | Yes | 35 | 7.7 | | 1.27 | | 0.78 | 2.07 | 0.34 |
| 5-10% | No | 149 | 90.3 | | - | |  |  |  |
|  | Yes | 16 | 9.7 | | 1.64 | | 0.88 | 3.05 | 0.12 |
| >10% | No | 84 | 86.6 | | - | |  |  |  |
|  | **Yes** | 13 | 13.4 | | **2.36** | | 1.20 | 4.66 | 0.01 |
| Feet of new sheep checked at purchase by farmer | | | | |  | |  |  |  |
| ≤2% | Never | 73 | 13.4 | | Reference category | | |  |  |
|  | Sometimes | 70 | 12.8 | | - | |  |  |  |
|  | Usually | 92 | 16.8 | | - | |  |  |  |
|  | Always | 176 | 32.2 | | - | |  |  |  |
|  | No new sheep purchased | 136 | 24.9 | | - | |  |  |  |
| >2-5% | Never | 75 | 16.7 | | - | |  |  |  |
|  | Sometimes | 72 | 16.0 | | 1.00 | | 0.63 | 1.59 | 1.00 |
|  | Usually | 109 | 24.3 | | 1.15 | | 0.75 | 1.76 | 0.51 |
|  | **Always** | 111 | 24.7 | | **0.61** | | 0.41 | 0.92 | 0.02 |
|  | **No new sheep purchased** | 82 | 18.3 | | **0.59** | | 0.38 | 0.90 | 0.01 |
| >5-10% | Never | 25 | 15.7 | | - | |  |  |  |
|  | Sometimes | 23 | 14.5 | | 0.96 | | 0.50 | 1.85 | 0.90 |
|  | Usually | 31 | 19.5 | | 0.98 | | 0.53 | 1.81 | 0.96 |
|  | Always | 44 | 27.7 | | 0.73 | | 0.42 | 1.28 | 0.27 |
|  | No new sheep purchased | 36 | 22.6 | | 0.77 | | 0.43 | 1.39 | 0.39 |
| >10% | Never | 16 | 16.5 | | - | |  |  |  |
|  | Sometimes | 19 | 19.6 | | 1.24 | | 0.59 | 2.60 | 0.57 |
|  | Usually | 27 | 27.8 | | 1.34 | | 0.67 | 2.67 | 0.41 |
|  | Always | 20 | 20.6 | | 0.52 | | 0.25 | 1.06 | 0.07 |
|  | No new sheep purchased | 15 | 15.5 | | 0.50 | | 0.24 | 1.08 | 0.08 |
| Feet of new sheep checked on arrival by farmer | | | | |  | |  |  |  |
| ≤2% | Never | 59 | 11.0 | | Reference category | | |  |  |
|  | Sometimes | 64 | 11.9 | | - | |  |  |  |
|  | Usually | 86 | 16.0 | | - | |  |  |  |
|  | Always | 197 | 36.8 | | - | |  |  |  |
|  | No new sheep purchased | 130 | 24.3 | | - | |  |  |  |
| >2-5% | Never | 60 | 13.5 | | - | |  |  |  |
|  | Sometimes | 58 | 13.0 | | 0.89 | | 0.54 | 1.48 | 0.65 |
|  | Usually | 110 | 24.7 | | 0.89 | | 0.80 | 1.99 | 0.33 |
|  | Always | 136 | 30.5 | | 0.68 | | 0.45 | 1.03 | 0.07 |
|  | **No new sheep purchased** | 82 | 18.4 | | **0.62** | | 0.39 | 0.98 | 0.04 |
| >5-10% | Never | 19 | 12.3 | | - | |  |  |  |
|  | Sometimes | 18 | 11.6 | | 0.87 | | 0.42 | 1.82 | 0.72 |
|  | Usually | 31 | 20.0 | | 1.12 | | 0.58 | 2.17 | 0.74 |
|  | Always | 57 | 36.8 | | 0.90 | | 0.50 | 1.63 | 0.72 |
|  | No new sheep purchased | 30 | 19.4 | | 0.72 | | 0.37 | 1.38 | 0.32 |
| >10% | Never | 10 | 10.5 | | - | |  |  |  |
|  | Sometimes | 14 | 14.7 | | 1.29 | | 0.53 | 3.13 | 0.57 |
|  | Usually | 28 | 29.5 | | 1.92 | | 0.87 | 4.25 | 0.11 |
|  | Always | 30 | 31.6 | | 0.90 | | 0.41 | 1.95 | 0.79 |
|  | No new sheep purchased | 13 | 13.7 | | 0.59 | | 0.24 | 1.42 | 0.24 |
| Feet of new sheep treated by farmer at arrival | | |  | |  | |  |  |  |
| ≤2% | Never | 140 | 27.6 | | Reference category | | |  |  |
|  | Sometimes | 79 | 15.6 | | - | |  |  |  |
|  | Usually | 40 | 7.9 | | - | |  |  |  |
|  | Always | 122 | 24.0 | | - | |  |  |  |
|  | No new sheep purchased | 127 | 25.0 | | - | |  |  |  |
| >2-5% | Never | 119 | 27.2 | | - | |  |  |  |
|  | Sometimes | 83 | 19.0 | | 1.24 | | 0.83 | 1.83 | 0.29 |
|  | **Usually** | 55 | 12.6 | | **1.62** | | 1.01 | 2.60 | 0.05 |
|  | Always | 100 | 22.8 | | 0.96 | | 0.67 | 1.38 | 0.84 |
|  | No new sheep purchased | 81 | 18.5 | | 0.75 | | 0.52 | 1.09 | 0.13 |
| >5-10% | Never | 42 | 28.0 | | - | |  |  |  |
|  | Sometimes | 24 | 16.0 | | 1.01 | | 0.57 | 1.79 | 0.97 |
|  | Usually | 16 | 10.7 | | 1.33 | | 0.68 | 2.62 | 0.40 |
|  | Always | 37 | 24.7 | | 1.01 | | 0.61 | 1.67 | 0.97 |
|  | No new sheep purchased | 31 | 20.7 | | 0.81 | | 0.48 | 1.37 | 0.44 |
| >10% | Never | 23 | 25.3 | | - | |  |  |  |
|  | Sometimes | 16 | 17.6 | | 1.23 | | 0.62 | 2.47 | 0.56 |
|  | Usually | 11 | 12.1 | | 1.67 | | 0.75 | 3.72 | 0.21 |
|  | Always | 28 | 30.8 | | 1.40 | | 0.76 | 2.55 | 0.28 |
|  | No new sheep purchased | 13 | 14.3 | | 0.62 | | 0.30 | 1.28 | 0.20 |
| Isolation of new sheep on arrival | |  |  | |  | |  |  |  |
| ≤2% | Never | 56 | 10.5 | | Reference category | | |  |  |
|  | Sometimes | 40 | 7.5 | | - | |  |  |  |
|  | Usually | 68 | 12.7 | | - | |  |  |  |
|  | Always | 250 | 46.6 | | - | |  |  |  |
|  | No new sheep purchased | 122 | 22.8 | | - | |  |  |  |
| >2-5% | Never | 59 | 13.2 | | - | |  |  |  |
|  | Sometimes | 45 | 10.1 | | 1.07 | | 0.61 | 1.87 | 0.82 |
|  | Usually | 70 | 15.7 | | 0.98 | | 0.60 | 1.60 | 0.93 |
|  | Always | 195 | 43.6 | | 0.74 | | 0.49 | 1.12 | 0.15 |
|  | **No new sheep purchased** | 78 | 17.5 | | **0.61** | | 0.38 | 0.96 | 0.03 |
| >5-10% | Never | 24 | 15.3 | | - | |  |  |  |
|  | Sometimes | 11 | 7.0 | | 0.64 | | 0.28 | 1.46 | 0.29 |
|  | Usually | 27 | 17.2 | | 0.93 | | 0.48 | 1.78 | 0.82 |
|  | Always | 64 | 40.8 | | 0.60 | | 0.34 | 1.04 | 0.07 |
|  | No new sheep purchased | 31 | 19.8 | | 0.59 | | 0.32 | 1.10 | 0.10 |
| >10% | Never | 14 | 14.7 | | - | |  |  |  |
|  | Sometimes | 8 | 8.4 | | 0.80 | | 0.31 | 2.09 | 0.65 |
|  | Usually | 17 | 17.9 | | 1.00 | | 0.45 | 2.21 | 1.00 |
|  | Always | 43 | 45.3 | | 0.69 | | 0.35 | 1.34 | 0.27 |
|  | **No new sheep purchased** | 13 | 13.7 | | **0.43** | | 0.19 | 0.97 | 0.04 |
| Length of isolation of new sheep | |  |  | |  | |  |  |  |
| ≤2% | <1 week | 32 | 8.8 | | Reference category | | |  |  |
|  | 1-3 weeks | 180 | 49.5 | | - | |  |  |  |
|  | >3 weeks | 152 | 41.8 | | - | |  |  |  |
| >2-5% | <1 week | 27 | 8.5 | | - | |  |  |  |
|  | 1-3 weeks | 172 | 54.3 | | 1.13 | | 0.65 | 1.97 | 0.66 |
|  | >3 weeks | 118 | 37.2 | | 0.92 | | 0.52 | 1.62 | 0.77 |
| >5-10% | <1 week | 14 | 13.3 | | - | |  |  |  |
|  | 1-3 weeks | 48 | 45.7 | | 0.61 | | 0.30 | 1.23 | 0.17 |
|  | >3 weeks | 43 | 41.0 | | 0.65 | | 0.32 | 1.32 | 0.23 |
| >10% | <1 week | 10 | 15.2 | | - | |  |  |  |
|  | 1-3 weeks | 36 | 54.6 | | 0.64 | | 0.29 | 1.42 | 0.27 |
|  | **>3 weeks** | 20 | 30.3 | | **0.42** | | 0.18 | 0.98 | 0.05 |
| No sheep left and returned to the farm | | |  | |  | |  |  |  |
| ≤2% | No | 208 | 37.6 | | Reference category | | |  |  |
|  | Yes | 345 | 62.4 | | - | |  |  |  |
| >2-5% | No | 171 | 37.5 | | - | |  |  |  |
|  | Yes | 285 | 62.5 | | 1.00 | | 0.78 | 1.30 | 0.97 |
| >5-10% | No | 76 | 46.1 | | - | |  |  |  |
|  | **Yes** | 89 | 53.9 | | **0.71** | | 0.50 | 1.00 | 0.05 |
| >10% | No | 49 | 50.5 | | - | |  |  |  |
|  | **Yes** | 48 | 49.5 | | **0.59** | | 0.38 | 0.91 | 0.02 |
| Sheep returned to farm from summer grazing | | |  | |  | |  |  |  |
| ≤2% | No | 481 | 87.0 | | Reference category | | |  |  |
|  | Yes | 72 | 13.0 | | - | |  |  |  |
| >2-5% | No | 386 | 84.7 | | - | |  |  |  |
|  | Yes | 70 | 15.4 | | 1.21 | | 0.85 | 1.73 | 0.29 |
| >5-10% | No | 140 | 84.9 | | - | |  |  |  |
|  | Yes | 25 | 15.2 | | 1.19 | | 0.73 | 1.95 | 0.48 |
| >10% | No | 76 | 78.4 | | - | |  |  |  |
|  | **Yes** | 21 | 21.7 | | **1.85** | | 1.07 | 3.18 | 0.03 |
| Sheep returned to farm from winter grazing | | |  | |  | |  |  |  |
| ≤2% | No | 420 | 76.0 | | Reference category | | |  |  |
|  | Yes | 133 | 24.1 | | - | |  |  |  |
| >2-5% | No | 344 | 75.4 | | - | |  |  |  |
|  | Yes | 112 | 24.6 | | 1.03 | | 0.77 | 1.37 | 0.85 |
| >5-10% | No | 112 | 67.9 | | - | |  |  |  |
|  | **Yes** | 53 | 32.1 | | **1.49** | | 1.02 | 2.19 | 0.04 |
| >10% | No | 64 | 66.0 | | - | |  |  |  |
|  | **Yes** | 33 | 34.0 | | **1.63** | | 1.02 | 2.59 | 0.04 |
| Isolation of returning sheep | |  |  | |  | |  |  |  |
| ≤2% | Never | 113 | 50.5 | | Reference category | | |  |  |
|  | Sometimes | 36 | 16.1 | | - | |  |  |  |
|  | Usually | 45 | 20.1 | | - | |  |  |  |
|  | Always | 30 | 13.4 | | - | |  |  |  |
| >2-5% | Never | 112 | 57.1 | | - | |  |  |  |
|  | Sometimes | 32 | 16.3 | | 0.90 | | 0.52 | 1.54 | 0.69 |
|  | Usually | 29 | 14.8 | | 0.65 | | 0.38 | 1.11 | 0.11 |
|  | Always | 23 | 11.7 | | 0.77 | | 0.42 | 1.41 | 0.40 |
| >5-10% | Never | 37 | 45.1 | | - | |  |  |  |
|  | Sometimes | 20 | 24.4 | | 1.70 | | 0.88 | 3.29 | 0.12 |
|  | Usually | 15 | 18.3 | | 1.02 | | 0.51 | 2.03 | 0.96 |
|  | Always | 10 | 12.2 | | 1.02 | | 0.45 | 2.28 | 0.97 |
| >10% | Never | 37 | 66.1 | | - | |  |  |  |
|  | Sometimes | 7 | 12.5 | | 0.59 | | 0.24 | 1.45 | 0.25 |
|  | Usually | 10 | 17.9 | | 0.68 | | 0.31 | 1.48 | 0.33 |
|  | **Always** | 2 | 3.6 | | **0.20** | | 0.05 | 0.89 | 0.03 |
| Mixing of sheep with neighbouring flocks | | |  | |  | |  |  |  |
| ≤2% | No | 19 | 3.5 | | Reference category | | |  |  |
|  | Yes | 513 | 95.7 | | - | |  |  |  |
|  | Unknown | 4 | 0.8 | | - | |  |  |  |
| >2-5% | No | 28 | 6.3 | | - | |  |  |  |
|  | **Yes** | 414 | 93.0 | | **0.55** | | 0.30 | 0.99 | 0.05 |
|  | Unknown | 3 | 0.7 | | 0.51 | | 0.10 | 2.54 | 0.41 |
| >5-10% | No | 13 | 8.1 | | - | |  |  |  |
|  | **Yes** | 147 | 91.9 | | 0.42 | | 0.20 | 0.87 | 0.02 |
|  | Unknown | 0 | 0.0 | | 0.00 | | 0.00 | 3.35E+68 | 0.91 |
| >10% | No | 2 | 2.1 | | - | |  |  |  |
|  | Yes | 95 | 97.9 | | 1.77 | | 0.40 | 7.75 | 0.45 |
|  | Unknown | 0 | 0.0 | | 0.00 | | 0.00 | 2.85E+28 | 0.86 |
| Farmer sex |  |  |  | |  | |  |  |  |
| ≤2% | Male | 465 | 84.4 | | Reference category | | |  |  |
|  | Female | 86 | 15.6 | | - | |  |  |  |
| >2-5% | Male | 392 | 87.1 | | - | |  |  |  |
|  | Female | 58 | 12.9 | | 0.80 | | 0.56 | 1.15 | 0.22 |
| >5-10% | Male | 150 | 90.9 | | - | |  |  |  |
|  | **Female** | 15 | 9.1 | | **0.54** | | 0.30 | 0.96 | 0.04 |
| >10% | Male | 76 | 81.7 | | - | |  |  |  |
|  | Female | 17 | 18.3 | | 1.21 | | 0.68 | 2.15 | 0.52 |
| Organic status | |  |  | |  | |  |  |  |
| ≤2% | Yes | 46 | 8.4 | | Reference category | | |  |  |
|  | No | 499 | 91.6 | | - | |  |  |  |
| >2-5% | Yes | 12 | 2.7 | | - | |  |  |  |
|  | **No** | 439 | 97.3 | | **3.37** | | 1.76 | 6.45 | <0.01 |
| >5-10% | Yes | 6 | 3.7 | | - | |  |  |  |
|  | **No** | 158 | 96.3 | | **2.43** | | 1.02 | 5.79 | 0.05 |
| >10% | Yes | 1 | 1.0 | | - | |  |  |  |
|  | **No** | 95 | 99.0 | | **8.75** | | 1.19 | 64.17 | 0.03 |
| Ewe stocking rate | |  |  | |  | |  |  |  |
| ≤2% | <4 ewes/acre | 239 | 45.0 | | Reference category | | |  |  |
|  | 4-8 ewes/acre | 267 | 50.3 | | - | |  |  |  |
|  | >8 ewes/acre | 25 | 4.7 | | - | |  |  |  |
| >2-5% | <4 ewes/acre | 213 | 47.8 | | - | |  |  |  |
|  | 4-8 ewes/acre | 215 | 48.2 | | 0.90 | | 0.70 | 1.17 | 0.44 |
|  | >8 ewes/acre | 18 | 4.0 | | 0.81 | | 0.43 | 1.52 | 0.51 |
| >5-10% | <4 ewes/acre | 71 | 45.2 | | - | |  |  |  |
|  | 4-8 ewes/acre | 81 | 51.6 | | 1.02 | | 0.71 | 1.47 | 0.91 |
|  | >8 ewes/acre | 5 | 3.2 | | 0.67 | | 0.25 | 1.82 | 0.44 |
| >10% | <4 ewes/acre | 31 | 32.6 | | - | |  |  |  |
|  | **4-8 ewes/acre** | 58 | 61.1 | | **1.67** | | 1.05 | 2.68 | 0.03 |
| Home bred replacement ewes | |  |  | |  | |  |  |  |
| ≤2% | No | 160 | 28.9 | | Reference category | | |  |  |
|  | Yes | 393 | 71.1 | | - | |  |  |  |
| >2-5% | No | 168 | 36.8 | | - | |  |  |  |
|  | **Yes** | 288 | 63.2 | | **0.70** | | 0.54 | 0.91 | 0.01 |
| >5-10% | No | 56 | 33.9 | | - | |  |  |  |
|  | Yes | 109 | 66.1 | | 0.79 | | 0.55 | 1.15 | 0.22 |
| >10% | No | 38 | 39.2 | | - | |  |  |  |
|  | **Yes** | 59 | 60.8 | | **0.63** | | 0.40 | 0.99 | 0.04 |

N: number, %: percent, OR: odds ratio, CI: confidence interval. Odds ratios significantly different from the baseline (according to Wald’s test for significance) are marked in bold. Significance was defined when p≤0.05.

Variables included had p≤0.05 in at least one category in the univariable analysis, or became significant in the final multivariate model.

**Supplementary Table 3: Multivariable multinomial regression models of nine sub-categories of variables from the questionnaire.**

a) Multinomial sub-model of variables to do with catching and recognising lame sheep associated with prevalence of lameness in lambs in 1222 flocks of sheep in England, 2012-2013.

| **Prevalence of lameness** | | **N** | **%** | **OR** | **Lower and upper 95% CI** | | **P-value** |
| --- | --- | --- | --- | --- | --- | --- | --- |
| **Locomotion score that farmer recognised sheep as lame at** | | | | |  |  |  |
| ≤2% | 1 | 300 | 56.3 | Reference Category | |  |  |
|  | 2 | 167 | 31.3 | - |  |  |  |
|  | 3 | 62 | 11.6 | - |  |  |  |
|  | >4 | 4 | 0.8 | - |  |  |  |
| >2-5% | 1 | 229 | 51.9 | - |  |  |  |
|  | 2 | 165 | 37.4 | 1.15 | 0.85 | 1.55 | 0.38 |
|  | 3 | 42 | 9.5 | 0.71 | 0.44 | 1.14 | 0.16 |
|  | >4 | 5 | 1.1 | 1.26 | 0.32 | 5.01 | 0.74 |
| >5-10% | 1 | 75 | 48.1 | - |  |  |  |
|  | 2 | 63 | 40.4 | 1.25 | 0.82 | 1.90 | 0.31 |
|  | **3** | 11 | 7.1 | **0.48** | 0.23 | 1.01 | 0.05 |
|  | **>4** | 7 | 4.5 | **4.85** | 1.23 | 19.19 | 0.02 |
| >10% | 1 | 40 | 43.5 | - |  |  |  |
|  | ***2*** | 35 | 38.0 | 1.66 | 0.95 | 2.89 | 0.08 |
|  | 3 | 14 | 15.2 | 1.80 | 0.83 | 3.89 | 0.14 |
|  | **>4** | 3 | 3.3 | **7.93** | 1.49 | 42.33 | 0.02 |
| **Minimum locomotion score when farmer decided to treat lame sheep** | | | | | |  |  |
| ≤2% | 1 | 161 | 30.2 | Reference Category | |  |  |
|  | 2 | 215 | 40.3 | - |  |  |  |
|  | 3 | 120 | 22.5 | - |  |  |  |
|  | >4 | 29 | 5.4 | - |  |  |  |
|  | Individuals not treated | 8 | 1.5 | - |  |  |  |
| >2-5% | 1 | 107 | 24.3 | - |  |  |  |
|  | 2 | 185 | 42.0 | 1.17 | 0.83 | 1.64 | 0.37 |
|  | **3** | 117 | 26.5 | **1.49** | 1.00 | 2.22 | 0.05 |
|  | >4 | 26 | 5.9 | 1.47 | 0.78 | 2.75 | 0.23 |
|  | Individuals not treated | 6 | 1.4 | 3.07 | 0.50 | 18.80 | 0.22 |
| >5-10% | 1 | 32 | 20.5 | - |  |  |  |
|  | 2 | 67 | 43.0 | 1.33 | 0.80 | 2.22 | 0.27 |
|  | 3 | 41 | 26.3 | 1.66 | 0.93 | 2.98 | 0.09 |
|  | **>4** | 15 | 9.6 | **2.43** | 1.06 | 5.56 | 0.04 |
|  | Individuals not treated | 1 | 0.6 | 2.23 | 0.15 | 32.99 | 0.56 |
| >10% | 1 | 27 | 29.4 | - |  |  |  |
|  | 2 | 38 | 41.3 | 0.80 | 0.44 | 1.45 | 0.46 |
|  | 3 | 22 | 23.9 | 0.79 | 0.39 | 1.61 | 0.52 |
|  | >4 | 5 | 5.4 | 0.65 | 0.20 | 2.09 | 0.47 |
|  | **Individuals not treated** | 0 | 0.0 | **0.00** | 0.00 | 0.00 | <0.001 |
| **Number of sheep lame when treated at minimum locomotion score recognised as lame** | | | | |  |  |  |
| ≤2% | 1 | 92 | 17.3 | Reference Category | |  |  |
|  | 2-5 | 289 | 54.2 | - |  |  |  |
|  | 6-10 | 79 | 14.8 | - |  |  |  |
|  | >10 | 64 | 12.0 | - |  |  |  |
|  | Individuals not treated | 9 | 1.7 | - |  |  |  |
| >2-5% | 1 | 52 | 11.8 | - |  |  |  |
|  | 2-5 | 238 | 54.0 | 1.30 | 0.88 | 1.93 | 0.19 |
|  | 6-10 | 77 | 17.5 | 1.52 | 0.94 | 2.46 | 0.09 |
|  | **>10** | 70 | 15.9 | **1.76** | 1.07 | 2.89 | 0.03 |
|  | Individuals not treated | 4 | 0.9 | 0.41 | 0.06 | 2.84 | 0.36 |
| >5-10% | 1 | 17 | 10.9 | - |  |  |  |
|  | 2-5 | 70 | 44.9 | 1.06 | 0.58 | 1.94 | 0.84 |
|  | **6-10** | 38 | 24.4 | **2.01** | 1.02 | 3.97 | 0.04 |
|  | **>10** | 31 | 19.9 | **2.06** | 1.02 | 4.16 | 0.04 |
|  | **Individuals not treated** | 0 | 0.0 | **0.00** | 0.00 | 0.00 | <0.01 |
| >10% | 1 | 9 | 9.8 | - |  |  |  |
|  | 2-5 | 38 | 41.3 | 1.22 | 0.55 | 2.68 | 0.63 |
|  | **6-10** | 22 | 23.9 | **2.44** | 1.02 | 5.82 | 0.05 |
|  | **>10** | 23 | 25.0 | **3.07** | 1.29 | 7.30 | 0.01 |
|  | **Individuals not treated** | 0 | 0.0 | **0.00** | 0.00 | 0.00 | <0.001 |
| **Time to treatment once sheep recognised as lame** | | | |  |  |  |  |
| ≤2% | <1 day | 53 | 9.9 | Reference Category | |  |  |
|  | 1-<3 days | 243 | 45.6 | - |  |  |  |
|  | >3-7 days | 179 | 33.6 | - |  |  |  |
|  | >7 days | 54 | 10.1 | - |  |  |  |
|  | Individuals not treated | 4 | 0.8 | - |  |  |  |
| >2-5% | <1 day | 26 | 5.9 | - |  |  |  |
|  | 1-<3 days | 190 | 43.1 | 1.55 | 0.92 | 2.60 | 0.10 |
|  | **>3-7 days** | 177 | 40.1 | **1.81** | 1.06 | 3.10 | 0.03 |
|  | >7 days | 47 | 10.7 | 1.50 | 0.79 | 2.84 | 0.22 |
|  | Individuals not treated | 1 | 0.2 | 0.57 | 0.04 | 8.88 | 0.69 |
| >5-10% | <1 day | 6 | 3.9 | - |  |  |  |
|  | 1-<3 days | 57 | 36.5 | 2.07 | 0.83 | 5.20 | 0.12 |
|  | **1-<3 days** | 72 | 46.2 | **3.18** | 1.26 | 8.03 | 0.01 |
|  | >3-7 days | 21 | 13.5 | 2.69 | 0.96 | 7.53 | 0.06 |
|  | **>7 days** | 0 | 0.0 | **0.11** | 0.11 | 0.11 | <0.001 |
| >10% | <1 day | 2 | 2.2 | - |  |  |  |
|  | 1-<3 days | 40 | 43.5 | 3.92 | 0.89 | 17.40 | 0.07 |
|  | **>3-7 days** | 40 | 43.5 | **4.83** | 1.08 | 21.65 | 0.04 |
|  | >7 days | 10 | 10.9 | 3.44 | 0.68 | 17.36 | 0.14 |
|  | **Individuals not treated** | 0 | 0.0 | **0.51** | 0.51 | 0.51 | <0.001 |
| **Use central handling facility to catch lame sheep** | | | | |  |  |  |
| ≤2% | No | 235 | 44.1 | Reference category | |  |  |
|  | Yes | 298 | 55.9 | - |  |  |  |
| >2-5% | No | 192 | 43.5 | - |  |  |  |
|  | Yes | 249 | 56.5 | 1.00 | 0.76 | 1.31 | 0.99 |
| >5-10% | No | 60 | 38.5 | - |  |  |  |
|  | Yes | 96 | 61.5 | 1.28 | 0.87 | 1.90 | 0.21 |
| >10% | No | 27 | 29.4 | - |  |  |  |
|  | **Yes** | 65 | 70.7 | **1.95** | 1.17 | 3.25 | 0.01 |
| **Use dog that can catch individuals to catch lame sheep** | | | | |  |  |  |
| ≤2% | No | 467 | 87.6 | Reference Category | |  |  |
|  | Yes | 66 | 12.4 | - |  |  |  |
| >2-5% | No | 382 | 86.6 | - |  |  |  |
|  | Yes | 59 | 13.4 | 1.13 | 0.77 | 1.67 | 0.53 |
| >5-10% | No | 128 | 82.1 | - |  |  |  |
|  | **Yes** | 28 | 18.0 | **1.79** | 1.08 | 2.99 | 0.03 |
| >10% | No | 72 | 78.3 | - |  |  |  |
|  | **Yes** | 20 | 21.7 | **2.67** | 1.47 | 4.84 | <0.001 |

b) Multinomial sub-model of variables to do with treating lambs lame with SFR and ID and prevalence of lameness in lambs in 899 flocks of sheep in England, 2012-2013.

| **Prevalence of lameness** | | **N** | **%** | **OR** | **Lower and upper 95% CI** | | **P-value** |
| --- | --- | --- | --- | --- | --- | --- | --- |
| **Treat lambs with SFR with antibiotic injection** | | | | |  |  |  |
| ≤2% | Always | 43 | 11.7 | Reference Category | | |  |
|  | Usually | 53 | 14.4 | - |  |  |  |
|  | Sometimes | 174 | 47.3 | - |  |  |  |
|  | Never | 98 | 26.6 | - |  |  |  |
| >2-5% | Always | 59 | 17.7 | - |  |  |  |
|  | Usually | 57 | 17.1 | 0.81 | 0.47 | 1.42 | 0.47 |
|  | **Sometimes** | 147 | 44.0 | **0.61** | 0.38 | 0.96 | 0.03 |
|  | **Never** | 71 | 21.3 | **0.55** | 0.33 | 0.93 | 0.02 |
| >5-10% | Always | 17 | 13.4 | - |  |  |  |
|  | Usually | 17 | 13.4 | 0.89 | 0.40 | 1.98 | 0.77 |
|  | Sometimes | 75 | 59.1 | 1.13 | 0.59 | 2.15 | 0.72 |
|  | Never | 18 | 14.2 | 0.51 | 0.23 | 1.10 | 0.09 |
| >10% | Always | 14 | 20.0 | - |  |  |  |
|  | Usually | 9 | 12.9 | 0.56 | 0.22 | 1.46 | 0.24 |
|  | Sometimes | 35 | 50.0 | 0.55 | 0.26 | 1.16 | 0.32 |
|  | **Never** | 12 | 17.1 | **0.38** | 0.15 | 0.92 | 0.03 |
| **Treat lambs with SFR with foot trim** | | | |  |  |  |  |
| ≤2% | Always | 53 | 14.4 | Reference Category | | |  |
|  | Usually | 76 | 20.7 | - |  |  |  |
|  | Sometimes | 153 | 41.6 | - |  |  |  |
|  | Never | 86 | 23.4 | - |  |  |  |
| >2-5% | Always | 65 | 19.5 | - |  |  |  |
|  | Usually | 72 | 21.6 | 0.86 | 0.51 | 1.47 | 0.59 |
|  | Sometimes | 156 | 46.7 | 1.04 | 0.64 | 1.69 | 0.88 |
|  | **Never** | 41 | 12.3 | **0.46** | 0.25 | 0.85 | 0.01 |
| >5-10% | Always | 32 | 25.2 | - |  |  |  |
|  | Usually | 25 | 19.7 | **0.71** | 0.35 | 1.43 | <0.01 |
|  | Sometimes | 64 | 50.4 | 0.94 | 0.50 | 1.76 | 0.84 |
|  | **Never** | 6 | 4.7 | **0.15** | 0.05 | 0.42 | <0.01 |
| >10% | Always | 15 | 21.4 | - |  |  |  |
|  | Usually | 15 | 21.4 | 0.97 | 0.39 | 2.42 | 0.94 |
|  | Sometimes | 38 | 54.3 | 1.53 | 0.66 | 3.53 | 0.32 |
|  | **Never** | **2** | **2.87** | **0.19** | 0.04 | 0.97 | 0.05 |
| **Treat lambs with ID with foot trim** | | | |  |  |  |  |
| ≤2% | Always | 15 | 4.1 | Reference Category | | |  |
|  | Usually | 35 | 9.5 | - |  |  |  |
|  | Sometimes | 167 | 45.4 | - |  |  |  |
|  | Never | 151 | 41.0 | - |  |  |  |
| >2-5% | Always | 23 | 6.7 | - |  |  |  |
|  | Usually | 36 | 10.8 | 0.72 | 0.30 | 1.71 | 0.46 |
|  | Sometimes | 157 | 47.1 | 0.63 | 0.29 | 1.34 | 0.23 |
|  | Never | 118 | 35.3 | 0.66 | 0.30 | 1.47 | 0.31 |
| >5-10% | Always | 16 | 12.6 | - |  |  |  |
|  | **Usually** | 11 | 8.7 | **0.33** | 0.11 | 0.96 | 0.04 |
|  | **Sometimes** | 59 | 46.5 | **0.33** | 0.14 | 0.81 | 0.01 |
|  | Never | 41 | 32.3 | 0.43 | 0.17 | 1.10 | 0.08 |
| >10% | Always | 8 | 11.4 | - |  |  |  |
|  | Usually | 8 | 11.4 | 0.39 | 0.11 | 1.46 | 0.16 |
|  | Sometimes | 40 | 57.1 | 0.35 | 0.12 | 1.06 | 0.06 |
|  | **Never** | 14 | 20.0 | **0.22** | 0.06 | 0.74 | 0.01 |
| **Use Lincospectin foot spray** | | |  |  |  |  |  |
| ≤2% | No | 340 | 92.4 | Reference Category | | |  |
|  | Yes | 28 | 7.6 | - |  |  |  |
| >2-5% | No | 293 | 87.7 | - |  |  |  |
|  | Yes | 41 | 12.3 | 1.72 | 1.02 | 2.89 | 0.72 |
| >5-10% | No | 114 | 89.8 | - |  |  |  |
|  | Yes | 13 | 10.2 | 1.48 | 0.72 | 3.04 | 0.28 |
| >10% | No | 57 | 81.4 | - |  |  |  |
|  | **Yes** | **13** | **18.6** | **3.37** | **1.59** | **7.13** | **<0.01** |
| **Use antibiotic aerosol foot spray** | | |  |  |  |  |  |
| ≤2% | No | 47 | 12.8 | Reference Category | | |  |
|  | Yes | 321 | 87.2 | - |  |  |  |
| >2-5% | No | 42 | 12.6 | - |  |  |  |
|  | Yes | 292 | 87.4 | 1.09 | 0.68 | 1.73 | 0.79 |
| >5-10% | No | 11 | 8.7 | - |  |  |  |
|  | Yes | 116 | 91.3 | 1.56 | 0.76 | 3.20 | 0.23 |
| >10% | No | 3 | 4.3 | - |  |  |  |
|  | **Yes** | 67 | 95.7 | **3.79** | 1.11 | 12.91 | 0.03 |

c) Multinomial sub-model of the association between lamb lameness prevalence and variables to do with treating ewes lame with SFR and ID in 980 flocks of sheep in England, 2012-2013.

| **Prevalence of lameness** | | **N** | **%** | **OR** | **Lower and upper 95% CI** | | **P-value** |
| --- | --- | --- | --- | --- | --- | --- | --- |
| **Treat ewes with SFR with antibiotic injection** | | | | |  |  |  |
| ≤2% | Always | 101 | 24.63 | Reference category | |  |  |
|  | Usually | 103 | 25.1 | - |  |  |  |
|  | Sometimes | 170 | 41.5 | - |  |  |  |
|  | Never | 36 | 8.8 | - |  |  |  |
| >2-5% | Always | 92 | 25.1 | - |  |  |  |
|  | Usually | 81 | 22.1 | 0.90 | 0.60 | 1.36 | 0.62 |
|  | Sometimes | 170 | 46.3 | 1.13 | 0.79 | 1.62 | 0.51 |
|  | Never | 24 | 6.5 | 0.81 | 0.44 | 1.49 | 0.49 |
| >5-10% | Always | 23 | 18.1 | - |  |  |  |
|  | **Usually** | 46 | 36.2 | **2.01** | 1.12 | 3.59 | 0.02 |
|  | Sometimes | 52 | 40.9 | 1.36 | 0.78 | 2.38 | 0.28 |
|  | Never | 6 | 4.7 | 0.82 | 0.30 | 2.22 | 0.69 |
| >10% | Always | 20 | 26.3 | - |  |  |  |
|  | Usually | 22 | 29.0 | 0.98 | 0.49 | 1.96 | 0.96 |
|  | Sometimes | 32 | 42.1 | 0.84 | 0.44 | 1.58 | 0.59 |
|  | Never | 2 | 2.6 | 0.29 | 0.06 | 1.35 | 0.11 |
| **Treat ewes with ID with foot trim** | | | |  |  |  |  |
| ≤2% | Always | 43 | 10.5 | Reference Category | |  |  |
|  | Usually | 66 | 16.1 | - |  |  |  |
|  | Sometimes | 187 | 45.6 | - |  |  |  |
|  | Never | 114 | 27.8 | - |  |  |  |
| >2-5% | Always | 38 | 10.4 | - |  |  |  |
|  | Usually | 62 | 16.9 | 1.11 | 0.63 | 1.95 | 0.72 |
|  | Sometimes | 166 | 45.2 | 1.03 | 0.63 | 1.68 | 0.92 |
|  | Never | 101 | 27.5 | 1.11 | 0.65 | 1.89 | 0.69 |
| >5-10% | Always | 14 | 11.0 | - |  |  |  |
|  | Usually | 22 | 17.3 | 0.99 | 0.45 | 2.18 | 0.98 |
|  | Sometimes | 56 | 44.1 | 0.87 | 0.44 | 1.72 | 0.68 |
|  | Never | 35 | 27.6 | 0.96 | 0.46 | 2.00 | 0.91 |
| >10% | Always | 10 | 13.2 | - |  |  |  |
|  | Usually | 16 | 21.1 | 0.95 | 0.39 | 2.36 | 0.92 |
|  | Sometimes | 39 | 51.3 | 0.77 | 0.35 | 1.70 | 0.52 |
|  | **Never** | 11 | 14.5 | **0.33** | 0.13 | 0.87 | 0.03 |
| **Treat ewes with SFR with foot spray** | | | |  |  |  |  |
| ≤2% | Always | 260 | 63.4 | Reference Category | |  |  |
|  | Usually | 89 | 21.7 | - |  |  |  |
|  | Sometimes | 45 | 11.0 | - |  |  |  |
|  | Never | 16 | 3.9 | - |  |  |  |
| >2-5% | Always | 262 | 71.4 | - |  |  |  |
|  | Usually | 71 | 19.4 | 0.78 | 0.54 | 1.13 | 0.18 |
|  | **Sometimes** | 29 | 7.9 | **0.60** | 0.36 | 1.01 | 0.05 |
|  | **Never** | 5 | 1.4 | **0.30** | 0.10 | 0.87 | 0.03 |
| >5-10% | Always | 87 | 68.5 | - |  |  |  |
|  | Usually | 26 | 20.5 | 0.81 | 0.49 | 1.36 | 0.43 |
|  | Sometimes | 13 | 10.2 | 0.81 | 0.41 | 1.61 | 0.55 |
|  | Never | 1 | 0.8 | 0.23 | 0.03 | 1.91 | 0.18 |
| >10% | Always | 52 | 68.4 | - |  |  |  |
|  | Usually | 14 | 18.4 | 0.89 | 0.46 | 1.72 | 0.72 |
|  | Sometimes | 9 | 11.8 | 1.19 | 0.52 | 2.71 | 0.68 |
|  | Never | 1 | 1.3 | 0.87 | 0.10 | 7.57 | 0.90 |
| **Use Lincospectin foot spray** | | |  |  |  |  |  |
| ≤2% | No | 375 | 91.5 | Reference Category | |  |  |
|  | Yes | 35 | 8.5 | - |  |  |  |
| >2-5% | No | 328 | 89.4 | - |  |  |  |
|  | Yes | 39 | 10.6 | 1.27 | 0.78 | 2.09 | 0.34 |
| >5-10% | No | 113 | 89.0 | - |  |  |  |
|  | Yes | 14 | 11.0 | 1.36 | 0.69 | 2.67 | 0.37 |
| >10% | No | 59 | 77.6 | - |  |  |  |
|  | **Yes** | 17 | 22.4 | **3.75** | 1.92 | 7.34 | <0.01 |
| **Use antibiotic aerosol foot spray** | | | |  |  |  |  |
| ≤2% | No | 51 | 12.4 | Reference Category | |  |  |
|  | Yes | 259 | 63.2 | - |  |  |  |
| >2-5% | No | 328 | 89.4 | - |  |  |  |
|  | Yes | 39 | 10.6 | 1.27 | 0.78 | 2.09 | 0.65 |
| >5-10% | No | 11 | 8.7 | - |  |  |  |
|  | Yes | 116 | 91.3 | 1.2 | 0.58 | 2.48 | 0.63 |
| >10% | No | 3 | 4.0 | - |  |  |  |
|  | **Yes** | 73 | 96.1 | **3.72** | 1.06 | 13.04 | 0.04 |

d) Multinomial sub-model of associations between routine foot trim of the flock and prevalence of lameness in lambs in 1206 flocks in England, 2012-2013.

| **Prevalence of lameness** | | **N** | **%** | **OR** | **Lower and upper 95% CI** | | **P value** |
| --- | --- | --- | --- | --- | --- | --- | --- |
| **Routine foot trim the flock** | | | | |  |  |  |
| ≤2% | Did not trim | 243 | 46.2 | Reference category | |  |  |
|  | Trimmed but no bleeding | 46 | 8.2 | - |  |  |  |
|  | Caused bleeding | 237 | 45.1 | - |  |  |  |
| ->2-5% | Did not trim | 183 | 42.3 | - |  |  |  |
|  | Trimmed but no bleeding | 24 | 5.5 | 0.69 | 0.41 | 1.18 | 0.17 |
|  | Caused bleeding | 226 | 52.2 | 1.27 | 0.97 | 1.65 | 0.08 |
| >5-10% | Did not trim | 74 | 48.4 | - |  |  |  |
|  | Trimmed but no bleeding | 7 | 4.6 | 0.50 | 0.22 | 1.15 | 0.10 |
|  | Caused bleeding | 72 | 47.1 | 1.00 | 0.69 | 1.45 | 0.99 |
| >10% | Did not trim | 25 | 25.5 | - |  |  |  |
|  | Trimmed but no bleeding | 5 | 5.3 | 1.10 | 0.40 | 3.03 | 0.85 |
|  | **Caused bleeding** | 65 | 69.2 | **2.78** | 1.68 | 4.58 | <0.001 |

e) Multinomial sub-model of associations between variables to do with footbathing the flock and prevalence of lameness in lambs in 833 flocks, in England, 2012-2013.

| **Prevalence of lameness** | | **N** | **%** | **OR** | **Lower and upper 95% CI** | | **P-value** |
| --- | --- | --- | --- | --- | --- | --- | --- |
| **Footbath lambs** | |  |  |  |  |  |  |
| ≤2% | No | 72 | 21.8 | Reference category | |  |  |
|  | Yes | 259 | 78.3 | - |  |  |  |
| >2-5% | No | 33 | 10.8 | 1.00 |  |  |  |
|  | **Yes** | 274 | 89.3 | **2.25** | 1.34 | 3.79 | <0.001 |
| >5-10% | No | 11 | 9.2 | 1.00 |  |  |  |
|  | Yes | 108 | 90.8 | 1.47 | 0.68 | 3.17 | 0.33 |
| >10% | No | 7 | 9.2 | 1.00 |  |  |  |
|  | Yes | 69 | 90.8 | 1.82 | 0.72 | 4.61 | 0.21 |
| **Footbath to treat SFR** | |  |  |  |  |  |  |
| ≤2% | No | 167 | 50.5 | Reference category | | |  |
|  | Yes | 164 | 49.6 | - | | |  |
| >2-5% | No | 138 | 45.0 | 1.00 |  |  |  |
|  | Yes | 169 | 55.1 | 1.15 | 0.82 | 1.61 | 0.41 |
| >5-10% | No | 55 | 46.2 | 1.00 |  |  |  |
|  | Yes | 64 | 53.8 | 0.83 | 0.53 | 1.30 | 0.42 |
| >10% | No | 23 | 30.3 | 1.00 |  |  |  |
|  | **Yes** | 53 | 69.7 | **2.08** | 1.18 | 3.67 | 0.01 |
| **Footbath to treat ID** | |  |  |  |  |  |  |
| ≤2% | No | 108 | 32.6 | Reference category | | |  |
|  | Yes | 223 | 67.4 | - |  |  |  |
| >2-5% | No | 84 | 27.4 | 1.00 |  |  |  |
|  | Yes | 221 | 72.0 | 0.98 | 0.66 | 1.45 | 0.92 |
| >5-10% | No | 13 | 10.9 | 1.00 |  |  |  |
|  | **Yes** | 106 | 89.1 | **3.76** | 1.90 | 7.43 | <0.001 |
| >10% | No | 16 | 21.1 | 1.00 |  |  |  |
|  | Yes | 60 | 79.0 | 1.25 | 0.64 | 2.45 | 0.51 |
| **Footbath to prevent ID** | |  |  |  |  |  |  |
| ≤2% | No | 148 | 44.7 | Reference category | | |  |
|  | Yes | 183 | 55.3 | - |  |  |  |
| >2-5% | No | 140 | 45.6 | 1.00 |  |  |  |
|  | Yes | 167 | 54.4 | 0.78 | 0.55 | 1.10 | 0.15 |
| >5-10% | No | 56 | 47.1 | 1.00 |  |  |  |
|  | Yes | 63 | 52.9 | 0.68 | 0.43 | 1.08 | 0.10 |
| >10% | No | 43 | 56.6 | 1.00 |  |  |  |
|  | **Yes** | 33 | 43.4 | **0.42** | 0.24 | 0.73 | <0.001 |
| **Routine footbathing of lambs at pasture** | | |  |  |  |  |  |
| ≤2% | Did not do routinely | 151 | 45.6 | Reference category | |  |  |
|  | Once a week | 3 | 0.9 | *-* |  |  |  |
|  | Once a fortnight | 36 | 10.9 | *-* |  |  |  |
|  | Once a month | 67 | 20.2 | *-* |  |  |  |
|  | Other | 74 | 22.4 | *-* |  |  |  |
| >2-5% | Did not do routinely | 113 | 36.8 | 1.00 |  |  |  |
|  | Once a week | 7 | 2.3 | 2.88 | 0.71 | 11.66 | 0.14 |
|  | Once a fortnight | 35 | 11.4 | 1.18 | 0.67 | 2.06 | 0.57 |
|  | **Once a month** | 87 | 28.3 | **1.56** | 1.01 | 2.42 | 0.05 |
|  | Other | 65 | 21.2 | 1.01 | 0.65 | 1.57 | 0.96 |
| >5-10% | Did not do routinely | 41 | 34.5 | 1.00 |  |  |  |
|  | **Once a week** | 6 | 5.0 | **8.06** | 1.82 | 35.67 | 0.01 |
|  | Once a fortnight | 14 | 11.8 | 1.35 | 0.63 | 2.88 | 0.43 |
|  | Once a month | 33 | 27.7 | 1.78 | 0.98 | 3.22 | 0.06 |
|  | Other | 25 | 21.0 | 1.12 | 0.61 | 2.04 | 0.71 |
| >10% | Did not do routinely | 19 | 25.0 | - |  |  |  |
|  | **Once a week** | 2 | 2.6 | **6.79** | 0.99 | 46.65 | 0.05 |
|  | **Once a fortnight** | 11 | 14.5 | **3.15** | 1.28 | 7.76 | 0.01 |
|  | **Once a month** | 24 | 31.6 | **3.32** | 1.59 | 6.90 | <0.001 |
|  | **Other** | 20 | 26.3 | **2.19** | 1.05 | 4.56 | 0.04 |
| **Footbathing of ewes before housing** | | |  |  |  |  |  |
| ≤2% | No | 215 | 65.0 | Reference Category | |  |  |
|  | Yes | 116 | 35.1 | *-* |  |  |  |
| >2-5% | No | 207 | 67.4 | - |  |  |  |
|  | Yes | 100 | 32.6 | 0.78 | 0.56 | 1.10 | 0.16 |
| >5-10% | No | 78 | 65.6 | - |  |  |  |
|  | Yes | 41 | 34.5 | 0.83 | 0.52 | 1.33 | 0.44 |
| >10% | **No** | 57 | 75.0 | - |  |  |  |
|  | **Yes** | 19 | 25.0 | **0.46** | 0.25 | 0.83 | 0.01 |

f) Multinomial sub-model of associations between variables to do with culling and replacing ewes and prevalence of lameness in lambs in 1135 flocks, in England, 2012-2013.

| **Prevalence of lameness** | | **N** | **%** | **OR** | **Lower and upper 95% CI** | | **P-value** |
| --- | --- | --- | --- | --- | --- | --- | --- |
| **Number of times sheep lame before culling** | | |  |  |  |  |  |
| ≤2% | Did not cull when lame | 227 | 56.3 | Reference category | |  |  |
|  | 1 | 25 | 5.1 | *-* |  |  |  |
|  | 2 | 70 | 14.2 | - |  |  |  |
|  | 2 or more | 104 | 21.1 | - |  |  |  |
|  | Persistently lame | 16 | 3.3 | - |  |  |  |
| >2-5% | Did not cull when lame | 212 | 52.1 | - |  |  |  |
|  | 1 | 9 | 2.2 | 0.51 | 0.23 | 1.13 | 0.10 |
|  | 2 | 45 | 11.1 | 0.91 | 0.59 | 1.38 | 0.64 |
|  | **2 or more** | 113 | 27.8 | **1.49** | 1.08 | 2.06 | 0.02 |
|  | **Persistently lame** | 28 | 6.9 | **2.24** | 1.17 | 4.26 | 0.01 |
| >5-10% | Did not cull when lame | 70 | 45.2 | - |  |  |  |
|  | 1 | 4 | 2.6 | 0.65 | 0.22 | 1.93 | 0.44 |
|  | 2 | 19 | 12.3 | 1.10 | 0.62 | 1.95 | 0.75 |
|  | **2 or more lame** | 56 | 36.1 | **2.16** | 1.41 | 3.29 | <0.001 |
|  | Persistently | 6 | 3.9 | 1.46 | 0.55 | 3.89 | 0.44 |
| >10% | Did not cull when lame | 38 | 46.9 | - |  |  |  |
|  | 1 | 0 | 0.0 | 0.00 | 0.00 | Inf | 0.97 |
|  | 2 | 9 | 11.1 | 1.01 | 0.46 | 2.20 | 0.98 |
|  | **2 or more** | 27 | 33.3 | **2.00** | 1.16 | 3.45 | 0.01 |
|  | **Persistently lame** | 7 | 8.6 | **3.21** | 1.23 | 8.33 | 0.02 |
| **Use of EID ear tag to identify sheep for culling** | | | | |  |  |  |
| ≤2% | No | 457 | 92.9 | Reference category | |  |  |
|  | Yes | 35 | 7.1 | - |  |  |  |
| >2-5% | No | 391 | 96.1 | - |  |  |  |
|  | **Yes** | 16 | 3.9 | **0.53** | 0.29 | 1.00 | 0.05 |
| >5-10% | No | 144 | 92.9 | - |  |  |  |
|  | Yes | 11 | 7.1 | 0.88 | 0.43 | 1.82 | 0.73 |
| >10% | No | 78 | 96.3 | - |  |  |  |
|  | Yes | 3 | 3.7 | 0.47 | 0.14 | 1.59 | 0.22 |
| **Did not breed replacement ewes** | | |  |  |  |  |  |
| ≤2% | No | 391 | 79.5 | Reference category | |  |  |
|  | Yes | 101 | 20.5 | - |  |  |  |
| >2-5% | No | 294 | 72.2 | - |  |  |  |
|  | **Yes** | 113 | 27.8 | **1.41** | 1.03 | 1.92 | 0.03 |
| >5-10% | No | 119 | 76.8 | - |  |  |  |
|  | Yes | 36 | 23.2 | 1.15 | 0.74 | 1.78 | 0.53 |
| >10% | No | 61 | 75.3 | - |  |  |  |
|  | Yes | 20 | 24.7 | 1.17 | 0.67 | 2.05 | 0.57 |

*g*) Vaccination of the whole flock with FootVax

No variables significant at p≤0.05 in univariable analysis so no sub-model built.

h) Multinomial sub-model of associations between variables to do with whole flock antibiotic treatment and prevalence of lameness in lambs in 1271 flocks in England, 2012-2013.

| **Prevalence of lameness** | | **N** | **%** | **OR** | **Lower and upper 95% CI** | | **P-value** |
| --- | --- | --- | --- | --- | --- | --- | --- |
| **Use of oxytetracycline LA for whole flock antibiotic injection** | | | | |  |  |  |
| ≤2% | No | 519 | 93.5 | Reference category | | |  |
|  | Yes | 34 | 6.2 | - |  |  |  |
| >2-5% | No | 421 | 92.3 | - |  |  |  |
|  | Yes | 35 | 7.7 | 1.27 | 0.78 | 2.07 | 0.34 |
| >5-10% | No | 149 | 90.3 | - |  |  |  |
|  | Yes | 6 | 3.6 | 1.64 | 0.88 | 3.05 | 0.12 |
| **>10%** | No | 84 | 86.6 | - |  |  |  |
|  | **Yes** | 13 | 13.4 | **2.36** | 1.20 | 4.66 | 0.01 |

h) Multinomial sub-model of associations between variables to do with farm biosecurity and prevalence of lameness in lambs in 1252 flocks in England, 2012-2013.

| **Prevalence of lameness** | | **N** | **%** | **OR** | **Lower and upper 95% CI** | | **P-value** |
| --- | --- | --- | --- | --- | --- | --- | --- |
| **Feet of new sheep checked before purchase** | | | |  |  |  |  |
| ≤2% | Never | 69 | 13.0 | Reference category | |  |  |
|  | Sometimes | 70 | 13.2 | - |  |  |  |
|  | Usually | 89 | 16.7 | - |  |  |  |
|  | Always | 170 | 32.0 | - |  |  |  |
|  | Did not purchase | 134 | 25.2 | - |  |  |  |
| >2-5% | Never | 73 | 16.6 | - |  |  |  |
|  | Sometimes | 71 | 16.2 | 0.95 | 0.59 | 1.51 | 0.82 |
|  | Usually | 106 | 24.2 | 1.13 | 0.73 | 1.74 | 0.59 |
|  | **Always** | 108 | 24.6 | **0.59** | 0.39 | 0.89 | 0.01 |
|  | **Did not purchase** | 81 | 18.5 | **0.57** | 0.37 | 0.88 | 0.01 |
| >5-10% | Never | 23 | 14.8 | - |  |  |  |
|  | Sometimes | 23 | 14.8 | 0.98 | 0.50 | 1.91 | 0.95 |
|  | Usually | 31 | 14.8 | 1.05 | 0.56 | 1.96 | 0.88 |
|  | Always | 42 | 27.1 | 0.73 | 0.41 | 1.31 | 0.29 |
|  | Did not purchase | 36 | 23.2 | 0.82 | 0.45 | 1.49 | 0.52 |
| >10% | Never | 16 | 16.5 | - |  |  |  |
|  | Sometimes | 19 | 19.6 | 1.19 | 0.57 | 2.51 | 0.64 |
|  | Usually | 27 | 27.8 | 1.29 | 0.65 | 2.60 | 0.47 |
|  | Always | 20 | 20.6 | 0.51 | 0.25 | 1.04 | 0.06 |
|  | Did not purchase | 15 | 15.5 | 0.49 | 0.23 | 1.04 | 0.06 |
| **Having sheep that did not return to the farm** | | | |  |  |  |  |
| ≤2% | No | 201 | 37.8 | Reference category | |  |  |
|  | Yes | 331 | 62.2 | - |  |  |  |
| >2-5% | No | 167 | 38.0 | - |  |  |  |
|  | Yes | 272 | 62.0 | 1.01 | 0.78 | 1.32 | 0.91 |
| >5-10% | No | 70 | 45.2 | 1.00 |  |  |  |
|  | Yes | 85 | 54.8 | 0.76 | 0.53 | 1.09 | 0.14 |
| >10% | No | 49 | 50.5 | 1.00 |  |  |  |
|  | **Yes** | 49 | 49.5 | **0.60** | 0.39 | 0.93 | 0.02 |
| **Mixing sheep with neighbouring flocks** | | |  |  |  |  |  |
| ≤2% | Yes | 19 | 3.6 | Reference category | |  |  |
|  | No | 509 | 95.7 | - |  |  |  |
|  | Unknown | 4 | 0.8 | *-* |  |  |  |
| >2-5% | Yes | 28 | 6.4 | - |  |  |  |
|  | **No** | 408 | 92.9 | **0.54** | 0.29 | 0.99 | 0.04 |
|  | Unknown | 3 | 0.7 | 0.48 | 0.09 | 2.44 | 0.38 |
| >5-10% | Yes | 12 | 7.7 | - |  |  |  |
|  | **No** | 143 | 92.3 | **0.46** | 0.22 | 0.97 | 0.04 |
|  | **Unknown** | 0 | 0.0 | **0.00** | 0.00 | 0.00 | <0.001 |
| >10% | Yes | 2 | 2.1 | - |  |  |  |
|  | No | 95 | 97.9 | 1.92 | 0.44 | 8.44 | 0.39 |
|  | Unknown | 0 | 0.0 | 0.00 | 0.00 | 1.21E+237 | 0.97 |

i) Multinomial sub-model of associations between variables relating to farm and farmer characteristics and prevalence of lameness in lambs in 1189 flocks in England, 2012-2013.

| **Prevalence of lameness** | | **N** | **%** | **OR** | **Lower and upper 95% CI** | | **P-value** |
| --- | --- | --- | --- | --- | --- | --- | --- |
| **Bought in replacement ewes** | | | |  |  |  |  |
| ≤2% | No | 252 | 47.5 | Reference category | |  |  |
|  | Yes | 279 | 52.5 | - |  |  |  |
|  |  |  |  |  |  |  |  |
| >2-5% | No | 167 | 37.4 | - |  |  |  |
|  | **Yes** | 279 | 62.6 | **1.52** | 1.18 | 1.97 | <0.001 |
| >5-10% | No | 74 | 47.1 | - |  |  |  |
|  | Yes | 83 | 52.9 | 1.01 | 0.71 | 1.45 | 0.95 |
| >10% | No | 74 | 47.1 | - |  |  |  |
|  | Yes | 83 | 52.9 | 1.01 | 0.71 | 1.45 | 0.95 |
| **Ewe stocking rate** | |  |  |  |  |  |  |
| ≤2% | <4 ewes/acre | 239 | 45.0 | Reference category | |  |  |
|  | 4-8 ewes/acre | 267 | 50.3 | - |  |  |  |
|  | >8 ewes/acre | 25 | 4.7 | - |  |  |  |
| >2-5% | <4 ewes/acre | 213 | 47.8 | - |  |  |  |
|  | 4-8 ewes/acre | 215 | 48.2 | 0.88 | 0.68 | 1.14 | 0.32 |
|  | >8 ewes/acre | 18 | 4.0 | 0.80 | 0.42 | 1.51 | 0.49 |
| >5-10% | <4 ewes/acre | 71 | 45.2 | - |  |  |  |
|  | 4-8 ewes/acre | 81 | 51.6 | 1.02 | 0.71 | 1.47 | 0.91 |
|  | >8 ewes/acre | 5 | 3.2 | 0.67 | 0.25 | 1.82 | 0.44 |
| >10% | <4 ewes/acre | 31 | 32.6 | - |  |  |  |
|  | **4-8 ewes/acre** | 58 | 61.1 | **1.62** | 1.01 | 2.59 | 0.05 |
|  | >8 ewes/acre | 6 | 6.3 | 1.83 | 0.70 | 4.83 | 0.22 |

N: number, %: percent, OR: odds ratio, CI: confidence interval. Odds ratios significantly different from the baseline (according to Wald’s test for significance) are marked in bold. Significance was defined when p≤0.05.

**Supplementary Table 4:** Strength of association between a treatment variable used in ewes and lambs for 732 flocks with complete responses to these questions in 2012-2013. (Cramer’s V statistics)

| **Treatment of ewes** | **Treatment of lambs** | | | | | |
| --- | --- | --- | --- | --- | --- | --- |
|  | Foot trimming | | Antibiotic Injection | | Foot Spray | |
|  | SFR | ID | SFR | ID | SFR | ID |
| Foot trimming (SFR) | 0.35* | 0.20* | 0.09 | 0.09 | 0.15* | 0.10* |
| Foot trimming (ID) | 0.21* | X | 0.08 | X | 0.10* | 0.10* |
| Antibiotic injection (SFR) | 0.12* | 0.10* | 0.50* | 0.21* | 0.14* | 0.10* |
| Antibiotic injection (ID) | 0.08 | X | X | X | 0.08 | 0.10* |
| Foot spray (SFR) | 0.13* | 0.11* | 0.11* | 0.08* | 0.63* | 0.53* |
| Foot spray (ID) | 0.12* | 0.12* | 0.11* | 0.11* | 0.48* | 0.65* |
| Separation of ewes (SFR) | X | X | X | X | 0.10 | 0.09 |
| Separation of ewes (ID) | X | X | X | X | X | X |

* indicates where p<0.05 from the chi squared test, indicating a significant association between the practices. X is where expected values were less than 5, so a p value from the chi squared test could not be calculated. Categories for the treatments include never, sometimes, usually and always.

The strongest associations were between using the same treatment for SFR in ewes and lambs, particularly for treatment of SFR - Cramer’s V of 0.50 for antibiotic injection, 0.35 for foot trimming and 0.63 for foot spray use, indicating moderate to strong associations between these variables.

**Calculation of Cramer’s V:**

Cramer’s V is a measure of effect size for nominal variables, calculated using the *lsr* package (Navarro, 2015). Many variables were significantly associated by chi-square tests, and use of Cramer’s V meant that some discrimination could be applied to which variables were most strongly associated with each other.

$\mathrm{Crame}r^{'}s V=\sqrt{\frac{x^{2}}{n(k-1)}}$ , where *x*^2^ is chi-square statistic, k is the number of rows or columns in the table and n is the total of observed counts.

**Interpretation of Cramer’s V:** Effect size was interpreted following guidelines from Rea and Parker (1992), where 0.00-<0.10 indicated

negligible association, 0.10-<0.20 indicated weak association, 0.20-<0.40 indicated moderate association, 0.40-<0.60 indicated relatively strong association, 0.60-<0.80 indicated a strong association and 0.80-1.00 indicated very strong association.

**Supplementary Table 5: Associations between variables significant in the multivariable multinomial model of lamb lameness prevalence in 842 flocks, 2012-2013.**

| **Variable** | **1** | **2** | **3** | **4** | **5** | **6** | **7** | **8** | **9** |
| --- | --- | --- | --- | --- | --- | --- | --- | --- | --- |
| 1. Use of foot trimming to treat lambs with SFR |  | 0.17 | 0.13 | 0.15 | X | X | 0.09 |  |  |
| 2. Use of antibiotic injection to treat lambs with SFR | 0.17 |  |  | 0.13 | 0.10 | X |  | 0.14 |  |
| 3. Causing bleeding in any sheep in routine trimming | 0.13 |  |  |  | 0.10 |  |  | 0.10 |  |
| 4. Footbathing to treat SFR | 0.15 | 0.13 |  |  |  | X | 0.13 | 0.11 | 0.10 |
| 5. Vaccinating sheep with SFR | X | 0.10 | 0.10 |  |  | X | X | X |  |
| 6. Score farmer recognised a sheep as lame at | X | X |  | X | X |  | X | X |  |
| 7. Number of times a sheep was lame before culling | 0.09 |  |  | 0.13 | X | X |  |  |  |
| 8. Isolation of new sheep on arrival |  | 0.14 | 0.10 | 0.11 | X | X |  |  | 0.27 |
| 9. Home breeding replacement ewes |  |  |  | 0.10 |  |  |  | 0.27 |  |

The Cramer’s V statistic for significant associations from the chi squared test of association (p<0.05) between variables that were significant in the final risk factor model are shown in the table. X indicates where an expected value was <5 so the chi squared statistic could not be calculated. Cramer’s V statistics are not shown for non-significant associations.

**Interpretation of Cramer’s V:**

Effect size was interpreted following guidelines from Rea and Parker (1992), where 0.00-<0.10 indicated

negligible association, 0.10-<0.20 indicated weak association, 0.20-<0.40 indicated moderate association, 0.40-<0.60 indicated relatively strong association, 0.60-<0.80 indicated a strong association and 0.80-1.00 indicated very strong association.

**Supplementary Table 6: Associations between variables significant in the multivariable multinomial model of prevalence of lameness in lambs – Model 1 (columns) and in a sub-model but not in the final model (rows) in 1271 flocks* of sheep, 2012-2013.**

|  | **Foot trimming lambs to treat SFR** | **Antibiotic injection to treat lambs with SFR** | **Causing bleeding in any sheep** | **Footbathing to treat SFR** | **Vaccinating sheep with SFR** | **Score farmer recognised a sheep as lame** | **Number of times a sheep was lame before culling** | **Isolation of new sheep on arrival** | **Home breeding replacement ewes** |
| --- | --- | --- | --- | --- | --- | --- | --- | --- | --- |
| Number of sheep treated at score lame | X | X |  | X | X | X | X | X | 0.09 |
| Score lame sheep treated at | X | X |  |  | X | X | X | X |  |
| Time to treatment | X | X | X | X | X | X | X | X | X |
| Catching with a central handling facility |  |  | 0.08 |  |  |  |  |  |  |
| Catching individual sheep with a dog |  | 0.15 | 0.12 |  |  | X | 0.14 | 0.10 |  |
| Foot trimming to treat lambs with ID | 0.44 | 0.08 | 0.18 |  | X | X | X |  |  |
| Foot spray to treat lambs with ID | 0.22 | 0.18 |  | 0.13 |  |  | 0.10 | 0.09 |  |
| Foot spray to treat lambs with SFR |  | 0.29 | 0.07 |  |  |  | 0.18 | 0.12 |  |
| Antibiotic injection to treat ewes with SFR |  |  |  |  | X | X | 0.12 |  |  |
| Foot trimming to treat ewes with ID |  |  |  |  |  |  | 0.11 |  |  |
| Use of Lincospectin spray to treat SFR/ID | 0.12 | 0.16 | 0.11 | 0.49 |  |  | 0.18 | 0.14 | 0.07 |
| Use of antibiotic aerosol spray to treat SFR/ID | 0.11 | 0.13 | 0.08 | 0.29 |  |  |  | 0.09 |  |
| Footbathing lambs | 0.09 | 0.15 |  | 0.55 |  |  | 0.18 | 0.13 | 0.07 |
| Footbathing to prevent ID |  |  |  | 0.13 |  |  |  |  |  |
| Footbathing to treat ID |  |  |  | 0.29 |  | X | 0.11 | 0.13 |  |
| Routine footbathing of lambs at pasture |  | 0.09 |  |  | X | X | X | 0.11 | 0.09 |
| Footbathing sheep before housing |  |  |  | 0.11 |  | X | 0.09 | 0.22 | 0.70 |
| Lameness indicator – EID ear tag |  |  |  |  | X | X | X |  |  |
| No replacement ewes bred | 0.08 | 0.09 | 0.10 | 0.10 |  | X | 0.11 | 0.48 | 0.32 |
| Oxytetracycline LA for whole flock injection |  | 0.11 |  | 0.09 |  |  | 0.09 |  | 0.10 |
| Checking feet before purchase | X | X | X | X | X | X | X | X | X |
| Having no returning sheep |  | 0.09 |  | 0.07 |  |  |  | 0.55 | 0.55 |
| Mixing with neighbouring flocks |  |  | 0.07 |  | X | X | X |  |  |
| Bought in replacement ewes | X | X |  | X | X | X | X | X | 0.09 |
| Ewe stocking rate | X | X |  |  | X | X | X | X |  |

Cramer’s V statistic is shown for any significant associations from the chi squared test of association (p<0.05). X indicates where an expected value was <5 so the chi squared statistic could not be calculated. Cramer’s V statistics are not shown for non-significant associations. *not all flocks have complete data for all variables.

**Supplementary Table 7a: Multivariable multinomial model of management practices and treatments used on lambs associated with prevalence of lameness in lambs in 842 flocks of sheep in England, 2012-2013.**

| **Variable and % lamb lameness category** | | **N** | **%** | **OR** | **Lower and Upper 95% CI** | | **P-value** |
| --- | --- | --- | --- | --- | --- | --- | --- |
| Antibiotic injection to treat lambs with SFR | | | |  |  |  |  |
| ≤2% | Always | 38 | 11.0 | Reference category | | |  |
|  | Usually | 48 | 13.9 | - |  |  |  |
|  | Sometimes | 160 | 46.4 | - |  |  |  |
|  | Never | 99 | 28.7 | - |  |  |  |
| >2-5% | Always | 58 | 18.0 | - |  |  |  |
|  | Usually | 55 | 17.1 | 0.64 | 0.36 | 1.16 | 0.14 |
|  | **Sometimes** | 141 | 43.8 | **0.48** | 0.29 | 0.80 | <0.01 |
|  | **Never** | 68 | 21.1 | **0.42** | 0.24 | 0.73 | <0.01 |
| >5-10% | Always | 15 | 13.5 | - |  |  |  |
|  | Usually | 15 | 13.5 | 0.82 | 0.34 | 1.96 | 0.65 |
|  | Sometimes | 63 | 56.8 | 1.01 | 0.49 | 2.07 | 0.98 |
|  | Never | 18 | 16.2 | 0.56 | 0.24 | 1.30 | 0.18 |
| >10% | Always | 11 | 17.2 | - |  |  |  |
|  | **Usually** | 7 | 10.9 | **0.32** | 0.10 | 1.00 | 0.05 |
|  | Sometimes | 37 | 57.8 | 0.54 | 0.23 | 1.25 | 0.15 |
|  | **Never** | 9 | 14.1 | **0.27** | 0.09 | 0.76 | 0.01 |
| Treat lambs with SFR by foot trim | | |  |  |  |  |  |
| <2% | Always | 54 | 15.7 | Reference category | | |  |
|  | Usually | 64 | 18.6 | - |  |  |  |
|  | Sometimes | 145 | 42.0 | - |  |  |  |
|  | Never | 82 | 23.8 | - |  |  |  |
| >2-5% | Always | 58 | 18.0 | - |  |  |  |
|  | Usually | 74 | 23.0 | 1.07 | 0.64 | 1.80 | 0.80 |
|  | Sometimes | 145 | 45.0 | 1.10 | 0.69 | 1.75 | 0.68 |
|  | Never | 45 | 14.0 | 0.69 | 0.40 | 1.19 | 0.18 |
| >5-10% | Always | 28 | 25.2 | - |  |  |  |
|  | Usually | 22 | 19.8 | 0.82 | 0.34 | 1.96 | 0.65 |
|  | Sometimes | 55 | 49.5 | 1.01 | 0.49 | 2.07 | 0.98 |
|  | Never | 6 | 5.4 | 0.56 | 0.24 | 1.30 | 0.18 |
|  | Always | 12 | 18.8 | - |  |  |  |
| >10% | Usually | 16 | 25.0 | 1.16 | 0.47 | 2.83 | 0.75 |
|  | Sometimes | 35 | 54.7 | 1.30 | 0.59 | 2.89 | 0.52 |
|  | **Never** | 1 | 1.6 | **0.11** | 0.01 | 0.87 | 0.04 |
| Vaccinate sheep with SFR | |  |  |  |  |  |  |
| ≤2% | No | 341 | 98.8 | Reference category | | |  |
|  | Yes | 4 | 1.2 | - |  |  |  |
| >2-5% | No | 309 | 96.0 | - |  |  |  |
|  | **Yes** | 13 | 4.0 | **3.32** | 1.03 | 10.67 | 0.04 |
| >5-10% | No | 108 | 97.3 | - |  |  |  |
|  | Yes | 3 | 2.7 | 1.89 | 0.39 | 9.18 | 0.43 |
| >10% | No | 62 | 96.9 | - |  |  |  |
|  | Yes | 2 | 3.1 | 3.65 | 0.59 | 22.71 | 0.16 |
| Footbath to treat SFR | |  |  |  |  |  |  |
| <2% | No | 242 | 70.1 | Reference category | | |  |
|  | Yes | 103 | 29.9 | - |  |  |  |
| >2-5% | No | 202 | 62.7 | - |  |  |  |
|  | Yes | 120 | 37.3 | 1.19 | 0.85 | 1.68 | 0.31 |
| >5-10% | No | 65 | 58.6 | - |  |  |  |
|  | Yes | 46 | 41.4 | 1.30 | 0.81 | 2.08 | 0.28 |
| >10% | No | 27 | 42.2 | - |  |  |  |
|  | **Yes** | 37 | 57.8 | **2.63** | 1.45 | 4.75 | <0.01 |
| Locomotion score farmer recognised sheep as lame | | | |  |  |  |  |
| ≤2% | 1 | 200 | 58.0 | Reference category | | |  |
|  | 2 | 112 | 32.5 | - |  |  |  |
|  | 3 | 31 | 9.0 | - |  |  |  |
|  | 4 or more | 2 | 0.6 | - |  |  |  |
| >2-5% | 1 | 167 | 51.9 | - |  |  |  |
|  | 2 | 121 | 37.6 | 1.30 | 0.92 | 1.83 | 0.14 |
|  | 3 | 32 | 9.9 | 1.27 | 0.72 | 2.22 | 0.41 |
|  | 4 or more | 2 | 0.6 | 1.12 | 0.15 | 8.20 | 0.91 |
| >5-10% | 1 | 52 | 46.8 | - |  |  |  |
|  | 2 | 46 | 41.4 | 1.58 | 0.98 | 2.57 | 0.06 |
|  | 3 | 9 | 8.1 | 1.18 | 0.51 | 2.75 | 0.70 |
|  | **4 or more** | 4 | 3.6 | **7.37** | 1.23 | 44.30 | 0.03 |
| >10% | 1 | 26 | 40.6 | - |  |  |  |
|  | 2 | 24 | 37.5 | 1.78 | 0.94 | 3.37 | 0.08 |
|  | **3** | 11 | 17.2 | **2.83** | 1.17 | 6.82 | 0.02 |
|  | **4 or more** | 3 | 4.7 | **10.61** | 1.47 | 76.28 | 0.02 |
| Routine foot trim the flock | |  |  |  |  |  |  |
| ≤2% | Did not trim | 163 | 47.2 | Reference category | | |  |
|  | Trimmed without bleeding | 26 | 7.5 | - |  |  |  |
|  | Caused bleeding | 156 | 45.2 | - |  |  |  |
| >2-5% | Did not trim | 132 | 41.0 | - |  |  |  |
|  | Trimmed without bleeding | 19 | 5.9 | 1.04 | 0.53 | 2.02 | 0.92 |
|  | Caused bleeding | 171 | 53.1 | 1.39 | 0.99 | 1.94 | 0.06 |
| >5-10% | Did not trim | 59 | 53.2 | - |  |  |  |
|  | Trimmed without bleeding | 2 | 1.8 | 0.24 | 0.05 | 1.08 | 0.06 |
|  | Caused bleeding | 50 | 45.0 | 0.79 | 0.50 | 1.27 | 0.33 |
| >10% | Did not trim | 12 | 18.8 | - |  |  |  |
|  | Trimmed without bleeding | 4 | 6.3 | 2.91 | 0.80 | 10.57 | 0.10 |
|  | **Caused bleeding** | 48 | 75.0 | **4.16** | 2.03 | 8.53 | 0.00 |
| Number of times sheep lame before culling | | | |  |  |  |  |
| ≤2% | Did not cull when lame | 183 | 53.0 | Reference category | | |  |
|  | Lame once | 18 | 5.2 | - | | |  |
|  | Lame twice | 50 | 14.5 | - |  |  |  |
|  | Lame >2 times | 79 | 22.9 | - |  |  |  |
|  | If persistently lame | 15 | 4.3 | - |  |  |  |
| >2-5% | Did not cull when lame | 154 | 47.8 | - |  |  |  |
|  | Lame once | 9 | 2.8 | 0.60 | 0.25 | 1.42 | 0.25 |
|  | Lame twice | 26 | 8.1 | 0.87 | 0.53 | 1.43 | 0.58 |
|  | Lame >2 times | 96 | 29.8 | 1.39 | 0.94 | 2.05 | 0.09 |
|  | **If persistently lame** | 27 | 8.4 | **2.10** | 1.04 | 4.21 | 0.04 |
| >5-10% | Did not cull when lame | 47 | 42.3 | - |  |  |  |
|  | Lame once | 2 | 1.8 | 0.48 | 0.10 | 2.25 | 0.35 |
|  | Lame twice | 15 | 13.5 | 1.41 | 0.70 | 2.82 | 0.34 |
|  | **Lame >2 times** | 42 | 37.8 | **2.12** | 1.25 | 3.58 | 0.01 |
|  | If persistently lame | 5 | 4.5 | 1.24 | 0.41 | 3.74 | 0.70 |
| >10% | Did not cull when lame | 33 | 51.6 | - |  |  |  |
|  | Lame once | 0 | 0.0 | 0.00 | 0.00 | 4.18E+109 | 0.94 |
|  | Lame twice | 7 | 10.9 | 0.87 | 0.34 | 2.21 | 0.77 |
|  | Lame >2 times | 20 | 31.3 | 1.54 | 0.78 | 3.04 | 0.21 |
|  | If persistently lame | 4 | 6.3 | 1.40 | 0.40 | 4.84 | 0.60 |
| Isolation of new sheep on arrival | | |  |  |  |  |  |
| ≤2% | Never | 33 | 9.6 | Reference category | | |  |
|  | Sometimes | 28 | 8.1 | - |  |  |  |
|  | Usually | 46 | 13.3 | - |  |  |  |
|  | Always | 164 | 47.5 | - |  |  |  |
|  | No new sheep | 74 | 21.4 | - |  |  |  |
| >2-5% | Never | 46 | 14.3 | - |  |  |  |
|  | Sometimes | 29 | 9.0 | 0.59 | 0.29 | 1.20 | 0.15 |
|  | Usually | 55 | 17.1 | 0.72 | 0.38 | 1.35 | 0.30 |
|  | **Always** | 138 | 42.9 | **0.51** | 0.30 | 0.87 | 0.01 |
|  | **No new sheep** | 54 | 16.8 | **0.53** | 0.29 | 0.97 | 0.04 |
| >5-10% | Never | 17 | 15.3 | - |  |  |  |
|  | Sometimes | 7 | 6.3 | 0.46 | 0.16 | 1.33 | 0.15 |
|  | Usually | 18 | 16.2 | 0.66 | 0.28 | 1.55 | 0.34 |
|  | Always | 49 | 44.1 | 0.52 | 0.25 | 1.06 | 0.07 |
|  | No new sheep | 20 | 18.0 | 0.71 | 0.31 | 1.64 | 0.43 |
| >10% | Never | 11 | 17.2 | - |  |  |  |
|  | Sometimes | 7 | 10.9 | 0.50 | 0.16 | 1.60 | 0.25 |
|  | Usually | 9 | 14.1 | 0.35 | 0.12 | 1.04 | 0.06 |
|  | Always | 30 | 46.9 | 0.45 | 0.19 | 1.06 | 0.07 |
|  | **No new sheep** | 7 | 10.9 | **0.29** | 0.09 | 0.88 | 0.03 |
| Home breeding replacement ewes | | |  |  |  |  |  |
| ≤2% | No | 98 | 28.4 | Reference category | | |  |
|  | Yes | 247 | 71.6 | - |  |  |  |
| >2-5% | No | 112 | 34.8 | - |  |  |  |
|  | Yes | 210 | 65.2 | 0.78 | 0.55 | 1.12 | 0.18 |
| >5-10% | No | 45 | 40.5 | - |  |  |  |
|  | **Yes** | 66 | 59.5 | **0.55** | 0.33 | 0.89 | 0.02 |
| >10% | No | 25 | 39.1 | - |  |  |  |
|  | Yes | 39 | 60.9 | 0.82 | 0.44 | 1.52 | 0.53 |

N: number, %: percent, OR: odds ratio, CI = confidence interval. Where p≤0.05, OR marked in bold, indicating a significant difference from the baseline (according to Wald’s test of significance).

**Supplementary Table 7b: Multivariable multinomial model of management practices and treatments used on ewes associated with prevalence of lameness in lambs in 973 flocks of sheep in England, 2012-2013.**

| **Variable and % lamb lameness category** | | **N** | **%** | **OR** | **Lower and Upper 95% CI** | | **P-value** |
| --- | --- | --- | --- | --- | --- | --- | --- |
| Treat ewes with SFR with antibiotic injection | | | | |  |  |  |
| ≤2% | Always | 99 | 23.6 | Reference category | | |  |
|  | Usually | 101 | 24.0 | - |  |  |  |
|  | Sometimes | 176 | 41.9 | - |  |  |  |
|  | Never | 44 | 10.5 | - |  |  |  |
| 2-5% | Always | 100 | 27.9 | - |  |  |  |
|  | Usually | 85 | 23.7 | 0.71 | 0.47 | 1.08 | 0.11 |
|  | Sometimes | 152 | 42.5 | 0.75 | 0.51 | 1.09 | 0.13 |
|  | **Never** | 21 | 5.9 | **0.47** | 0.25 | 0.88 | 0.02 |
| 5-10% | Always | 21 | 17.2 | - |  |  |  |
|  | **Usually** | 49 | 40.2 | **2.09** | 1.15 | 3.81 | 0.02 |
|  | Sometimes | 46 | 37.7 | 1.23 | 0.68 | 2.23 | 0.49 |
|  | Never | 6 | 4.9 | 0.72 | 0.26 | 1.98 | 0.53 |
| >10% | Always | 19 | 26.0 | - |  |  |  |
|  | Usually | 20 | 27.4 | 0.81 | 0.39 | 1.67 | 0.56 |
|  | Sometimes | 33 | 45.2 | 0.80 | 0.41 | 1.55 | 0.51 |
|  | **Never** | 1 | 1.4 | **0.11** | 0.01 | 0.92 | 0.04 |
| Footbath to treat SFR | |  |  |  |  |  |  |
| ≤2% | No | 300 | 71.4 | Reference category | | |  |
|  | Yes | 120 | 28.6 | - |  |  |  |
| 2-5% | No | 229 | 64.0 | - |  |  |  |
|  | Yes | 129 | 36.0 | 1.28 | 0.94 | 1.75 | 0.12 |
| 5-10% | No | 72 | 59.0 | - |  |  |  |
|  | **Yes** | 50 | 41 | **1.61** | 1.04 | 2.49 | 0.03 |
| >10% | No | 32 | 43.8 | - |  |  |  |
|  | **Yes** | 41 | 56.2 | **2.86** | 1.68 | 4.85 | <0.01 |
| Vaccinate sheep with SFR | |  |  |  |  |  |  |
| ≤2% | No | 416 | 99.0 | Reference category | | |  |
|  | Yes | 4 | 1.0 | - |  |  |  |
| 2-5% | No | 345 | 96.4 | - |  |  |  |
|  | Yes | 13 | 3.6 | 4.46 | 1.40 | 14.20 | 0.01 |
| 5-10% | No | 118 | 96.7 | - |  |  |  |
|  | Yes | 4 | 3.3 | 3.40 | 0.80 | 14.48 | 0.10 |
| >10% | No | 70 | 95.9 | **-** |  |  |  |
|  | **Yes** | 3 | 4.1 | **7.00** | 1.40 | 35.07 | 0.02 |
| Locomotion score farmer recognised sheep as lame | | | | |  |  |  |
| <2% | 1 | 240 | 57.1 | Reference category | | |  |
|  | 2 | 132 | 31.4 | - |  |  |  |
|  | 3 | 45 | 10.7 | - |  |  |  |
|  | 4 or more | 3 | 0.7 | - |  |  |  |
| 2-5% | 1 | 185 | 51.7 | - |  |  |  |
|  | 2 | 134 | 37.4 | 1.33 | 0.97 | 1.83 | 0.08 |
|  | 3 | 36 | 10.1 | 1.08 | 0.66 | 1.78 | 0.75 |
|  | 4 or more | 3 | 0.8 | 1.31 | 0.26 | 6.68 | 0.75 |
| 5-10% | 1 | 58 | 47.5 | - |  |  |  |
|  | 2 | 49 | 40.2 | 1.38 | 0.88 | 2.17 | 0.16 |
|  | 3 | 10 | 8.2 | 0.93 | 0.43 | 2.01 | 0.86 |
|  | **4 or more** | 5 | 4.1 | **6.14** | 1.36 | 27.69 | 0.02 |
| >10% | 1 | 30 | 41.1 | - |  |  |  |
|  | **2** | 29 | 39.7 | **1.81** | 1.02 | 3.22 | 0.04 |
|  | 3 | 11 | 15.1 | 1.98 | 0.87 | 4.49 | 0.10 |
|  | **4 or more** | 3 | 4.1 | **7.14** | 1.28 | 39.85 | 0.03 |
| Routine foot trimming the flock | | |  |  |  |  |  |
| ≤2% | Did not trim | 199 | 47.4 | Reference category | | |  |
|  | Trimmed without bleeding | 34 | 8.1 | - |  |  |  |
|  | Caused bleeding | 187 | 44.5 | - |  |  |  |
| 2-5% | Did not trim | 151 | 42.2 | - |  |  |  |
|  | Trimmed without bleeding | 21 | 5.9 | 0.90 | 0.49 | 1.65 | 0.74 |
|  | **Caused bleeding** | 186 | 52.0 | **1.38** | 1.01 | 1.87 | 0.04 |
| 5-10% | Did not trim | 63 | 51.6 | - |  |  |  |
|  | Trimmed without bleeding | 4 | 3.3 | 0.44 | 0.15 | 1.32 | 0.14 |
|  | Caused bleeding | 55 | 45.1 | 0.95 | 0.62 | 1.47 | 0.83 |
| >10% | Did not trim | 18 | 24.7 | - |  |  |  |
|  | Trimmed without bleeding | 5 | 6.8 | 2.04 | 0.66 | 6.27 | 0.21 |
|  | **Caused bleeding** | 50 | 68.5 | **3.25** | 1.77 | 5.95 | <0.01 |
| Culling sheep when lame | |  |  |  |  |  |  |
| ≤2% | Did not cull | 224 | 53.3 | Reference category | | |  |
|  | 1 | 19 | 4.5 | - |  |  |  |
|  | 1-<2 | 63 | 15.0 | - |  |  |  |
|  | >2 | 98 | 23.3 | - |  |  |  |
|  | Persistently lame | 16 | 3.8 | - |  |  |  |
| 2-5% | Did not cull | 177 | 49.4 | - |  |  |  |
|  | 1 | 9 | 2.5 | 0.62 | 0.27 | 1.42 | 0.25 |
|  | 1-<2 | 41 | 11.5 | 0.75 | 0.47 | 1.18 | 0.22 |
|  | >2 | 103 | 28.8 | 1.21 | 0.85 | 1.73 | 0.29 |
|  | **Persistently lame** | 28 | 7.8 | **2.22** | 1.14 | 4.33 | 0.02 |
| 5-10% | Did not cull | 54 | 44.3 | - |  |  |  |
|  | 1 | 3 | 2.5 | 0.59 | 0.16 | 2.14 | 0.42 |
|  | 1-<2 | 16 | 13.1 | 1.10 | 0.57 | 2.10 | 0.78 |
|  | **>2** | 44 | 36.1 | **1.63** | 1.00 | 2.66 | 0.05 |
|  | Persistently lame | 5 | 4.1 | 1.31 | 0.45 | 3.84 | 0.62 |
| >10% | Did not cull | 36 | 49.3 | - |  |  |  |
|  | **1** | 0 | 0.0 | **0.00** | 0.00 | 0.00 | <0.01 |
|  | 1-<2 | 8 | 11.0 | 0.81 | 0.34 | 1.89 | 0.62 |
|  | >2 | 24 | 32.9 | 1.39 | 0.76 | 2.56 | 0.29 |
|  | Persistently lame | 5 | 6.8 | 1.80 | 0.58 | 5.58 | 0.31 |
| Isolation of new sheep on arrival | | |  |  |  |  |  |
| ≤2% | Never | 41 | 9.8 | Reference category | | |  |
|  | Usually | 33 | 7.9 | - |  |  |  |
|  | Sometimes | 55 | 13.1 | - |  |  |  |
|  | Always | 196 | 46.7 | - |  |  |  |
|  | No new arrivals | 95 | 22.6 | - |  |  |  |
| 2-5% | Never | 49 | 13.7 | - |  |  |  |
|  | Usually | 37 | 10.3 | 0.83 | 0.43 | 1.58 | 0.57 |
|  | Sometimes | 60 | 16.8 | 0.79 | 0.44 | 1.4 | 0.41 |
|  | **Always** | 154 | 43.0 | **0.58** | 0.36 | 0.95 | 0.03 |
|  | **No new arrivals** | 58 | 16.2 | **0.49** | 0.28 | 0.84 | 0.01 |
| 5-10% | Never | 18 | 14.8 | - |  |  |  |
|  | Usually | 9 | 7.4 | 0.52 | 0.20 | 1.35 | 0.18 |
|  | Sometimes | 21 | 17.2 | 0.70 | 0.32 | 1.53 | 0.37 |
|  | Always | 51 | 41.8 | 0.53 | 0.27 | 1.03 | 0.06 |
|  | No new arrivals | 23 | 18.9 | 0.62 | 0.30 | 1.31 | 0.21 |
| >10% | Never | 13 | 17.8 | - |  |  |  |
|  | Usually | 6 | 8.2 | 0.38 | 0.12 | 1.17 | 0.09 |
|  | Sometimes | 11 | 15.1 | 0.44 | 0.17 | 1.16 | 0.10 |
|  | **Always** | 35 | 47.9 | **0.46** | 0.21 | 0.99 | 0.05 |
|  | **No new arrivals** | 8 | 11.0 | **0.25** | 0.09 | 0.69 | 0.01 |

N: number, %: percent, OR: odds ratio, CI = confidence interval. Where p≤0.05, OR marked in bold, indicating a significant difference from the baseline (according to Wald’s test of significance).

**Supplementary Table 8: Multivariable multinomial model of management practices and ewe treatments for severe footrot (SFR) and interdigital dermatitis (ID) associated with prevalence of lameness in ewes in 964 flocks of sheep in England, 2012-2013.**

| **Prevalence of lameness** | | | | **N** | **%** | **OR** | **Lower and upper 95% CI** | | **P-value** |
| --- | --- | --- | --- | --- | --- | --- | --- | --- | --- |
| **Treat ewes with SFR with antibiotic injection** | | | | | |  |  |  |  |
| ≤2% | | Always | | 85 | 27.0 | Reference category | | |  |
|  | | Usually | | 69 | 21.9 | - |  |  |  |
|  | | Sometimes | | 116 | 36.8 | - |  |  |  |
|  | | Never | | 45 | 14.3 | - |  |  |  |
| >2-5% | | Always | | 101 | 24.2 | - |  |  |  |
|  | | Usually | | 116 | 27.8 | 1.29 | 0.83 | 2.01 | 0.25 |
|  | | Sometimes | | 184 | 44.1 | 1.08 | 0.72 | 1.63 | 0.70 |
|  | | **Never** | | 16 | 3.8 | **0.28** | 0.14 | 0.56 | <0.001 |
| >5-10% | | Always | | 37 | 22.3 | - |  |  |  |
|  | | Usually | | 50 | 30.1 | 1.22 | 0.68 | 2.19 | 0.50 |
|  | | Sometimes | | 70 | 42.2 | 0.88 | 0.51 | 1.50 | 0.63 |
|  | | **Never** | | 9 | 5.4 | **0.32** | 0.13 | 0.80 | 0.01 |
| >10% | | Always | | 15 | 22.7 | - |  |  |  |
|  | | Usually | | 17 | 25.8 | 1.18 | 0.51 | 2.73 | 0.70 |
|  | | Sometimes | | 32 | 48.5 | 0.94 | 0.44 | 2.00 | 0.87 |
|  | | Never | | 2 | 3.0 | 0.21 | 0.04 | 1.07 | 0.06 |
| **Footbath to treat SFR** | | | |  |  |  |  |  |  |
| ≤2% | | No | | 241 | 76.5 | Reference category | |  |  |
|  | | Yes | | 74 | 23.5 | - |  |  |  |
| >2-5% | | No | | 263 | 63.1 | - |  |  |  |
|  | | **Yes** | | 154 | 36.9 | **1.51** | 1.05 | 2.18 | 0.03 |
| >5-10% | | No | | 87 | 52.4 | - |  |  |  |
|  | | **Yes** | | 79 | 47.6 | **2.40** | 1.52 | 3.79 | <0.01 |
| >10% | | No | | 34 | 51.5 | - |  |  |  |
|  | | **Yes** | | 32 | 48.5 | **2.81** | 1.51 | 5.22 | <0.01 |
| **Footbath to prevent ID** | | | |  |  |  |  |  |  |
| ≤2% | | No | | 219 | 69.5 | Reference category | |  |  |
|  | | Yes | | 96 | 30.5 | - |  |  |  |
| >2-5% | | No | | 246 | 59.0 | - |  |  |  |
|  | | Yes | | 171 | 41.0 | 1.22 | 0.86 | 1.74 | 0.26 |
| >5-10% | | No | | 99 | 59.6 | - |  |  |  |
|  | | Yes | | 67 | 40.4 | 1.01 | 0.64 | 1.59 | 0.97 |
| >10% | | No | | 50 | 75.8 | - |  |  |  |
|  | | **Yes** | | 16 | 24.2 | **0.44** | 0.22 | 0.87 | 0.02 |
| **Vaccinate ewes** | | | |  |  |  |  |  |  |
| ≤2% | | No | | 248 | 78.7 | Reference category | |  |  |
|  | | Yes | | 67 | 21.3 | - |  |  |  |
| >2-5% | | No | | 347 | 83.2 | - |  |  |  |
|  | | **Yes** | | 70 | 16.8 | **0.62** | 0.41 | 0.94 | 0.02 |
| 5-10% | | No | | 148 | 89.2 | - |  |  |  |
|  | | **Yes** | | 18 | 10.8 | **0.39** | 0.21 | 0.71 | <0.01 |
| >10% | | No | | 56 | 84.8 | - |  |  |  |
|  | | Yes | | 10 | 15.2 | 0.65 | 0.29 | 1.45 | 0.30 |
| **Time to treatment** | | | |  |  |  |  |  |  |
| ≤2% | | First day seen | | 30 | 9.5 | Reference category | |  |  |
|  | | <3 days | | 159 | 50.5 | - |  |  |  |
|  | | <7 days | | 98 | 31.1 | - |  |  |  |
|  | | >7 days | | 26 | 8.3 | - |  |  |  |
|  | | Did not treat any lame sheep | | 2 | 0.6 | - |  |  |  |
| >2-5% | | First day seen | | 17 | 4.1 | - |  |  |  |
|  | | <3 days | | 190 | 45.6 | 1.88 | 0.95 | 3.73 | 0.07 |
|  | | **<7 days** | | 165 | 39.6 | **2.48** | 1.22 | 5.03 | 0.01 |
|  | | **>7 days** | | 45 | 10.8 | **2.81** | 1.19 | 6.59 | 0.02 |
|  | | **Did not treat any lame sheep** | | 0 | 0.0 | **0.00** | 0.00 | 0.00 | <0.01 |
| >5-10% | | First day seen | | 8 | 4.8 |  |  |  |  |
|  | | <3 days | | 56 | 33.7 | 0.94 | 0.38 | 2.35 | 0.89 |
|  | | <7 days | | 85 | 51.2 | 2.13 | 0.85 | 5.33 | 0.11 |
|  | | >7 days | | 17 | 10.2 | 1.63 | 0.54 | 4.95 | 0.39 |
|  | | **Did not treat any lame sheep** | | 0 | 0.0 | **0.00** | 0.00 | 0.00 | <0.01 |
| >10% | | First day seen | | 1 | 1.5 |  |  |  |  |
|  | | <3 days | | 22 | 33.3 | 3.68 | 0.45 | 30.04 | 0.22 |
|  | | **<7 days** | | 33 | 50.0 | **9.44** | 1.15 | 77.58 | 0.04 |
|  | | **>7 days** | | 10 | 15.2 | **11.10** | 1.20 | 102.86 | 0.03 |
|  | | **Did not treat any lame sheep** | | 0 | 0.0 | **0.74** | 0.74 | 0.74 | <0.01 |
|  |  | |  |  |  |  |  |  |  |
| ≤2% | | 1 | | 67 | 21.3 | Reference category | |  |  |
|  | | 2-5 | | 177 | 56.2 | - |  |  |  |
|  | | 6-10 | | 41 | 13.0 | - |  |  |  |
|  | | >10 | | 27 | 8.6 | - |  |  |  |
|  | | Did not treat individuals | | 3 | 1.0 | - |  |  |  |
| >2-5% | | 1 | | 54 | 12.9 | - |  |  |  |
|  | | 2-5 | | 223 | 53.5 | 1.19 | 0.76 | 1.86 | 0.45 |
|  | | 6-10 | | 78 | 18.7 | 1.52 | 0.86 | 2.67 | 0.15 |
|  | | **>10** | | 59 | 14.1 | **1.88** | 1.00 | 3.51 | 0.05 |
|  | | Did not treat individuals | | 3 | 0.7 | 2.42 | 0.20 | 28.77 | 0.49 |
| >5-10% | | 1 | | 9 | 5.4 | - |  |  |  |
|  | | **2-5** | | 89 | 53.6 | **2.78** | 1.26 | 6.10 | 0.01 |
|  | | **6-10** | | 36 | 21.7 | **3.85** | 1.59 | 9.29 | <0.01 |
|  | | **>10** | | 30 | 18.1 | **5.99** | 2.36 | 15.22 | <0.01 |
|  | | Did not treat individuals | | 2 | 1.2 | 8.69 | 0.56 | 134.73 | 0.12 |
| >10% | | 1 | | 8 | 12.1 | - |  |  |  |
|  | | 2-5 | | 16 | 24.2 | 0.46 | 0.18 | 1.18 | 0.11 |
|  | | 6-10 | | 24 | 36.4 | 2.34 | 0.89 | 6.15 | 0.08 |
|  | | **>10** | | 18 | 27.3 | **2.96** | 1.05 | 8.38 | 0.04 |
|  | | **Did not treat individuals** | | 0 | 0.0 | **0.00** | 0.00 | 0.00 | <0.01 |
| **Routine foot trim the flock** | | | |  |  |  |  |  |  |
| ≤2% | | Did not trim | | 174 | 55.2 | Reference category | |  |  |
|  | | No bleeding caused | | 26 | 8.3 | - |  |  |  |
|  | | Bleeding caused | | 115 | 36.5 | - |  |  |  |
| >2-5% | | Did not trim | | 183 | 43.9 | - |  |  |  |
|  | | No bleeding caused | | 25 | 6.0 | 1.28 | 0.68 | 2.42 | 0.44 |
|  | | **Bleeding caused** | | 209 | 50.1 | **1.71** | 1.22 | 2.40 | <0.01 |
|  | | Did not trim | | 58 | 34.9 | - |  |  |  |
| >5-10% | | No bleeding caused | | 8 | 4.8 | 1.46 | 0.59 | 3.63 | 0.41 |
|  | | **Bleeding caused** | | 100 | 60.2 | **2.42** | 1.56 | 3.76 | <0.01 |
|  | | Did not trim | |  |  | - |  |  |  |
| >10% | | **No bleeding caused** | | 4 | 6.1 | **4.11** | 1.15 | 14.65 | 0.03 |
|  | | **Bleeding caused** | | 47 | 71.2 | **5.53** | 2.80 | 10.93 | <0.01 |
| **Isolation of new sheep on arrival** | | | | |  |  |  |  |  |
| ≤2% | | Never | | 29 | 9.2 | Reference category | |  |  |
|  | | Sometimes | | 24 | 7.6 | - |  |  |  |
|  | | Usually | | 41 | 13.0 | - |  |  |  |
|  | | Always | | 150 | 47.6 | - |  |  |  |
|  | | No new arrivals | | 71 | 22.5 | - |  |  |  |
| >2-5% | | Never | | 56 | 13.4 | - |  |  |  |
|  | | Sometimes | | 34 | 8.2 | 0.49 | 0.23 | 1.03 | 0.06 |
|  | | Usually | | 59 | 14.1 | 0.54 | 0.28 | 1.03 | 0.06 |
|  | | **Always** | | 190 | 45.6 | **0.47** | 0.27 | 0.82 | 0.01 |
|  | | **No new arrivals** | | 78 | 18.7 | **0.55** | 0.30 | 1.00 | 0.05 |
| >5-10% | | Never | | 24 | 14.5 | - |  |  |  |
|  | | Sometimes | | 22 | 13.3 | 0.68 | 0.29 | 1.63 | 0.39 |
|  | | Usually | | 37 | 22.3 | 0.85 | 0.39 | 1.85 | 0.69 |
|  | | **Always** | | 56 | 33.7 | **0.34** | 0.17 | 0.67 | <0.01 |
|  | | **No new arrivals** | | 27 | 16.3 | **0.46** | 0.21 | 0.98 | 0.04 |
| >10% | | Never | | 12 | 18.2 | - |  |  |  |
|  | | **Sometimes** | | 4 | 6.1 | **0.18** | 0.05 | 0.72 | 0.01 |
|  | | Usually | | 9 | 13.6 | 0.35 | 0.12 | 1.06 | 0.06 |
|  | | **Always** | | 32 | 48.5 | **0.38** | 0.16 | 0.90 | 0.03 |
|  | | **No new arrivals** | | 9 | 13.6 | **0.26** | 0.09 | 0.75 | 0.01 |
| **Number of times sheep lame before culling** | | | | | |  |  |  |  |
| ≤2% | | Did not cull lame sheep | | 174 | 55.2 |  |  |  |  |
|  | | 1 | | 20 | 6.3 | Reference category | |  |  |
|  | | 1 to <2 | | 47 | 14.9 | - |  |  |  |
|  | | >2 | | 64 | 20.3 | - |  |  |  |
|  | | Persistently lame | | 10 | 3.2 | - |  |  |  |
| >2-5% | | Did not cull lame sheep | | 188 | 45.1 | - |  |  |  |
|  | | **1** | | 8 | 1.9 | **0.37** | 0.15 | 0.91 | 0.03 |
|  | | 1 to <2 | | 55 | 13.2 | 1.03 | 0.64 | 1.64 | 0.92 |
|  | | **>2** | | 136 | 32.6 | **1.83** | 1.24 | 2.72 | <0.01 |
|  | | **Persistently lame** | | 30 | 7.2 | **2.29** | 1.06 | 4.95 | 0.04 |
| 5-10% | | Did not cull lame sheep | | 88 | 53.0 | - |  |  |  |
|  | | 1 | | 3 | 1.8 | 0.30 | 0.08 | 1.11 | 0.07 |
|  | | 1 to <2 | | 19 | 11.4 | 0.71 | 0.37 | 1.36 | 0.30 |
|  | | >2 | | 46 | 27.7 | 1.25 | 0.75 | 2.09 | 0.39 |
|  | | Persistently lame | | 10 | 6.0 | 1.45 | 0.55 | 3.82 | 0.45 |
| >5-10% | | Did not cull lame sheep | | 33 | 50.0 | - |  |  |  |
|  | | 1 | | 1 | 1.5 | 0.38 | 0.05 | 3.19 | 0.38 |
|  | | 1 to <2 | | 7 | 10.6 | 0.68 | 0.26 | 1.75 | 0.42 |
|  | | >2 | | 21 | 31.8 | 1.65 | 0.83 | 3.30 | 0.16 |
|  | | Persistently lame | | 4 | 6.1 | 1.70 | 0.46 | 6.36 | 0.43 |
|  | |  | |  |  |  |  |  |  |

N: number, %: percent, OR: odds ratio, CI = confidence interval. Where p≤0.05, OR marked in bold, indicating a significant difference from the baseline (according to Wald’s test of significance).

**Supplementary Table 9 : Numbers of observed and predicted flocks in each category of the model (≤2%, >2-5%, >5-10% and >10% LiL or LiE) for the final multivariate models for prevalence of lameness and flock managements as explanatory variables.**

| **Number of flocks observed in category** |  | **Model 1** |  |  |  | **Model 2** |  |  |  | **Model 3** |  |  |
| --- | --- | --- | --- | --- | --- | --- | --- | --- | --- | --- | --- | --- |
|  | **Number of flocks predicted in category** | | | | **Number of flocks predicted in category** | | | | **Number of flocks predicted in category** | | | |
|  | ≤2% | >2-5% | >5-10% | >10% | ≤2% | >2-5% | >5-10% | >10% | ≤2% | >2-5% | >5-10% | >10% |
| ≤2% | 240 | 100 | 0 | 5 | 307 | 110 | 1 | 2 | 157 | 149 | 7 | 2 |
| >2-5% | 142 | 176 | 2 | 2 | 189 | 167 | 2 | 0 | 89 | 310 | 11 | 7 |
| >5-10% | 49 | 52 | 8 | 2 | 67 | 50 | 4 | 1 | 25 | 124 | 14 | 3 |
| >10% | 16 | 42 | 3 | 3 | 28 | 41 | 3 | 1 | 6 | 52 | 4 | 4 |

Model 1: prevalence of lameness in lambs with flock managements and treatments for lambs as explanatory variables

Model 2: prevalence of lameness in lambs with flock managements and treatments for ewes as explanatory variables

Model 3: prevalence of lameness in ewes with flock managements and treatments for ewes as explanatory variables

P values from Hosmer-Lemeshow goodness of fit test test (Jay et al., 2017) indicated no lack of fit of any model:

Model 1: p = 0.8508

Model 2: p = 0.7405

Model 3: p = 0.6596

**Latent Class Analysis**

**Supplementary Table 10: Fit statistics for latent class models**

a) Fit statistics for the latent class models tested (2-7 classes) for models for type and frequency of treatment of lambs with interdigital dermatitis or severe footrot in 823 flocks of sheep in England, 2012-2013.

| **Number of classes** | **Model 1: Treatments for lambs** | | |  |
| --- | --- | --- | --- | --- |
|  | **AIC** | **BIC** | **G^2^** | **Log likelihood** |
| 1 | 13028.20 | 13131.88 | 3485.89 | -6492.10 |
| 2 | 12450.22 | 12662.31 | 2861.92 | -6180.11 |
| 3 | 12207.37 | 12527.85 | 2573.06 | -6035.69 |
| 4 | 12071.02 | 12499.90 | 2390.71 | -5944.51 |
| 5 | 11970.96 | 12508.24 | 2244.65 | -5871.48 |
| 6 | 11891.13 | 12536.81 | 2118.83 | -5808.57 |
| 7 | 11840.86 | 12594.93 | 2022.55 | -5760.43 |

AIC = Akaike Information Criterion; BIC = Bayesian Information Criterion; G^2^ = likelihood/deviance statistic

*All flocks have complete data

b) Fit statistics for the latent class models tested (2-7 classes) for models for type and frequency of treatment of ewes with interdigital dermatitis or severe footrot in 908 flocks of sheep in England, 2012-2013.

| **Number of classes** | **Model 2: Treatments for ewes** | | |  |
| --- | --- | --- | --- | --- |
|  | **AIC** | **BIC** | **G^2^** | **Log likelihood** |
| 1 | 14158.61 | 14264.46 | 3288.63 | -7057.31 |
| 2 | 13619.21 | 13835.71 | 2703.22 | -6764.60 |
| 3 | 13494.80 | 13821.97 | 2532.82 | -6679.40 |
| 4 | 13370.82 | 13808.64 | 2362.84 | -6594.41 |
| 5 | 13289.63 | 13838.11 | 2235.64 | -6530.81 |
| 6 | 13248.76 | 13907.90 | 2148.78 | -6487.38 |
| 7 | 13219.86 | 13989.66 | 2073.88 | -6449.93 |

AIC = Akaike Information Criterion; BIC = Bayesian Information Criterion; G^2^ = likelihood/deviance statistic

*All flocks have complete data

**Supplementary Table 11: Class conditional probabilities and standard errors for the four class model for treatments used for ID and SFR in ewes and lambs**

a) Class conditional response probabilities that a farmer used a type and frequency of treatment for lambs with interdigital dermatitis or severe footrot, and standard errors for 823 flocks of sheep in England, 2012-2013.

| **Treatment** | **Frequency of use** | **Class conditional response probability (standard error)** | | | |
| --- | --- | --- | --- | --- | --- |
|  |  | **LC1** | **LC2** | **LC3** | **LC4** |
| *Treatment for SFR* |  |  |  |  |  |
| Antibiotic injection | Never | 0.44 (0.07) | 0.16 (0.03) | 0.38 (0.03) | 0.00 (0.00) |
|  | Sometimes | 0.47 (0.07) | 0.54 (0.04) | 0.43 (0.03) | 0.50 (0.04) |
|  | Usually | 0.03 (0.02) | 0.25 (0.04) | 0.08 (0.02) | 0.19 (0.03) |
|  | Always | 0.06 (0.03) | 0.05 (0.02) | 0.11 (0.02) | 0.31 (0.04) |
| Foot spray | Never | 0.23 (0.07) | 0.00 (0.00) | 0.00 (0.00) | 0.00 (0.00) |
|  | Sometimes | 0.75 (0.07) | 0.10 (0.03) | 0.00 (0.00) | 0.01 (0.01) |
|  | Usually | 0.02 (0.04) | 0.82 (0.05) | 0.04 (0.02) | 0.01 (0.02) |
|  | Always | 0.00 (0.00) | 0.08 (0.04) | 0.96 (0.02) | 0.98 (0.02) |
| Foot trimming | Never | 0.36 (0.06) | 0.14 (0.03) | 0.08 (0.02) | 0.14 (0.03) |
|  | Sometimes | 0.60 (0.06) | 0.45 (0.04) | 0.47 (0.03) | 0.39 (0.04) |
|  | Usually | 0.01 (0.01) | 0.35 (0.04) | 0.20 (0.03) | 0.19 (0.03) |
|  | Always | 0.02 (0.02) | 0.06 (0.02) | 0.24 (0.03) | 0.28 (0.04) |
| *Treatment for ID* |  |  |  |  |  |
| Antibiotic injection | Never | 0.66 (0.07) | 0.44 (0.05) | 0.99 (0.01) | 0.00 (0.00) |
|  | Sometimes | 0.32 (0.06) | 0.40 (0.04) | 0.01 (0.01) | 0.79 (0.03) |
|  | Usually | 0.00 (0.00) | 0.16 (0.03) | 0.00 (0.00) | 0.11 (0.02) |
|  | Always | 0.02 (0.02) | 0.00 (0.00) | 0.00 (0.00) | 0.10 (0.02) |
| Foot spray | Never | 0.20 (0.06) | 0.00 (0.00) | 0.00 (0.00) | 0.00 (0.00) |
|  | Sometimes | 0.61 (0.07) | 0.15 (0.03) | 0.05 (0.02) | 0.02 (0.01) |
|  | Usually | 0.09 (0.03) | 0.69 (0.05) | 0.08 (0.02) | 0.03 (0.02) |
|  | Always | 0.11 (0.04) | 0.17 (0.04) | 0.86 (0.03) | 0.94 (0.02) |
| Foot trimming | Never | 0.65 (0.06) | 0.33 (0.04) | 0.37 (0.03) | 0.22 (0.04) |
|  | Sometimes | 0.33 (0.06) | 0.49 (0.04) | 0.49 (0.03) | 0.52 (0.04) |
|  | Usually | 0.01 (0.01) | 0.16 (0.03) | 0.07 (0.02) | 0.13 (0.03) |
|  | Always | 0.01 (0.01) | 0.02 (0.01) | 0.08 (0.02) | 0.13 (0.03) |
| Time to treatment |  |  |  |  |  |
|  | <1 day | 0.06 (0.02) | 0.06 (0.02) | 0.07 (0.02) | 0.08 (0.02) |
|  | 1-<3 days | 0.37 (0.05) | 0.43 (0.04) | 0.42 (0.03) | 0.47 (0.03) |
|  | >3-<7 days | 0.38 (0.05) | 0.39 (0.04) | 0.40 (0.03) | 0.37 (0.03) |
|  | >7 days | 0.19 (0.04) | 0.12 (0.03) | 0.12 (0.02) | 0.08 (0.02) |
|  | None treated | 0.01 (0.01) | 0.00 (0.00) | 0.00 (0.00) | 0.00 (0.00) |

b) Class conditional response probabilities that a farmer used a type and frequency of treatment on ewes with interdigital dermatitis or severe footrot, and standard error for 908 flocks of sheep in England, 2012-2013.

| **Treatment** | **Frequency of use** | **Predicted latent class** | |  |  |
| --- | --- | --- | --- | --- | --- |
|  |  | **LC1** | **LC2** | **LC3** | **LC4** |
| *Treatment for SFR* | |  |  |  |  |
| Antibiotic injection | Never | 0.13 (0.06) | 0.00 (0.00) | 0.09 (0.02) | 0.07 (0.01) |
|  | Sometimes | 0.44 (0.07) | 0.26 (0.05) | 0.52 (0.04) | 0.45 (0.03) |
|  | Usually | 0.19 (0.06) | 0.26 (0.04) | 0.35 (0.04) | 0.23 (0.02) |
|  | Always | 0.24 (0.06) | 0.47 (0.05) | 0.05 (0.02) | 0.25 (0.02) |
| Foot spray | Never | 0.21 (0.09) | 0.01 (0.01) | 0.01 (0.01) | 0.00 (0.00) |
|  | Sometimes | 0.66 (0.09) | 0.03 (0.02) | 0.11 (0.03) | 0.00 (0.00) |
|  | Usually | 0.08 (0.05) | 0.11 (0.04) | 0.77 (0.05) | 0.02 (0.01) |
|  | Always | 0.04 (0.05) | 0.85 (0.04) | 0.11 (0.05) | 0.97 (0.01) |
| Foot trimming | Never | 0.09 (0.05) | 0.14 (0.03) | 0.03 (0.01) | 0.00 (0.00) |
|  | Sometimes | 0.41 (0.07) | 0.67 (0.06) | 0.26 (0.04) | 0.01 (0.01) |
|  | Usually | 0.25 (0.06) | 0.19 (0.07) | 0.51 (0.04) | 0.32 (0.03) |
|  | Always | 0.25 (0.06) | 0.00 (0.00) | 0.20 (0.04) | 0.67 (0.03) |
| *Treatment for ID* | |  |  |  |  |
| Antibiotic injection | Never | 0.53 (0.08) | 0.32 (0.05) | 0.56 (0.05) | 0.52 (0.03) |
|  | Sometimes | 0.33 (0.06) | 0.48 (0.05) | 0.31 (0.04) | 0.34 (0.02) |
|  | Usually | 0.07 (0.04) | 0.12 (0.03) | 0.12 (0.03) | 0.08 (0.01) |
|  | Always | 0.07 (0.04) | 0.09 (0.03) | 0.00 (0.00) | 0.05 (0.01) |
| Foot spray | Never | 0.23 (0.08) | 0.00 (0.00) | 0.04 (0.02) | 0.00 (0.00) |
|  | Sometimes | 0.75 (0.09) | 0.01 (0.02) | 0.05 (0.03) | 0.04 (0.01) |
|  | Usually | 0.00 (0.00) | 0.08 (0.03) | 0.76 (0.05) | 0.10 (0.02) |
|  | Always | 0.02 (0.03) | 0.90 (0.04) | 0.15 (0.04) | 0.86 (0.02) |
| Foot trimming | Never | 0.51 (0.09) | 0.57 (0.05) | 0.30 (0.05) | 0.12 (0.02) |
|  | Sometimes | 0.35 (0.06) | 0.43 (0.05) | 0.44 (0.04) | 0.50 (0.03) |
|  | Usually | 0.09 (0.05) | 0.00 (0.00) | 0.22 (0.03) | 0.21 (0.02) |
|  | Always | 0.06 (0.03) | 0.00 (0.00) | 0.03 (0.02) | 0.17 (0.02) |
| Time to treatment | |  |  |  |  |
|  | <1 day | 0.06 (0.03) | 0.08 (0.03) | 0.06 (0.02) | 0.06 (0.01) |
|  | 1-<3 days | 0.28 (0.06) | 0.48 (0.05) | 0.32 (0.04) | 0.49 (0.02) |
|  | >3-<7 days | 0.45 (0.07) | 0.40 (0.05) | 0.46 (0.04) | 0.36 (0.02) |
|  | >7 days | 0.20 (0.05) | 0.04 (0.02) | 0.16 (0.03) | 0.10 (0.01) |
|  | None treated | 0.01 (0.01) | 0.00 (0.00) | 0.01 (0.01) | 0.00 (0.00) |
