# Supplementary material for: Management Practices Associated With Prevalence of Lameness in Lambs in 2012–2013 in 1,271 English Sheep Flocks
Source: Front Vet Sci. 2020 Oct 27;7:519601. doi: 10.3389/fvets.2020.519601 (PMC7653190; doi:10.3389/fvets.2020.519601)
Supplement: Supplementary file 2 [file Table_5.docx]

Table 5 Comparison of the three multivariable multinomial models of factors associated with prevalence of lameness in lambs and ewes in 842, 973 and 964 flocks of sheep (respectively) in England, 2012-2013

| **Predictor** | **Model 1: management of lameness in lambs / prevalence of lameness in lambs** | | | **Model 2: management of lameness in ewes / prevalence of lameness in lambs** | | | **Model 3: management of lameness in ewes / prevalence of lameness in ewes** | | |
| --- | --- | --- | --- | --- | --- | --- | --- | --- | --- |
| **Category of lameness** | **>2-5%** | **>5-10%** | **>10%** | **>2-5%** | **>5-10%** | **>10%** | **>2-5%** | **>5-10%** | **>10%** |
| **Treat lambs with severe footrot with antibiotic injection** (baseline always) | | | |  |  |  |  |  |  |
| Usually | 0.64  (0.36-1.16) | 0.82  (0.34-1.96) | **0.32**  **(0.10-1.00)** | x | x | x | x | x | x |
| Sometimes | **0.48**  **(0.29-0.80)** | 1.01  (0.49-2.21) | 0.54  (0.23-1.25) | x | x | x | x | x | x |
| Never | **0.42**  **(0.24-0.73)** | 0.56  (0.24-1.30) | **0.27**  **(0.09-0.76)** | x | x | x | x | x | x |
| **Treat ewes with severe footrot with antibiotic injection** (baseline always) | | | |  |  |  |  |  |  |
| Usually | x | x | x | 0.71  (0.47-1.08) | **2.09**  **(1.15-3.81)** | 0.81  (0.39-1.67) | 1.29  (0.83-2.01) | 1.22  (0.68-2.19) | 1.18  (0.51-2.73) |
| Sometimes | x | x | x | 0.75  (0.51-1.09) | 1.23  (0.68-2.23) | 0.80  (0.41-1.55) | 1.08  (0.72-1.63) | 0.88  (0.51-1.50) | 0.94  (0.44-2.00) |
| Never | x | x | x | **0.47**  **(0.25-0.88)** | 0.72  (0.26-1.98) | **0.11 (0.01-0.92)** | **0.28**  **(0.14-0.56)** | **0.32**  **(0.13-0.80)** | 0.21  (0.04-1.07) |
| **Foot trimming used to treat lambs with severe footrot** (baseline always) | | | |  |  |  |  |  |  |
| Usually | 1.07  (0.64-1.80) | 0.82  (0.34-1.96) | 1.16  (0.47-2.83) | x | x | x | x | x | x |
| Sometimes | 1.10  (0.69-1.75) | 1.01  (0.49-2.21) | 1.30  (0.59-2.89) | x | x | x | x | x | x |
| Never | 0.69  (0.04-1.19) | 0.56  (0.24-1.30) | **0.11**  **(0.01-0.87)** | x | x | x | x | x | x |
| **Footbath used to prevent interdigital dermatitis (**baseline no) | | | |  |  |  |  |  |  |
| Yes | x | x | x | x | x | x | 1.22  (0.86-1.74) | 1.01  (0.64-1.59) | **0.44**  **(0.22-0.87)** |
| **Footbath used to treat severe footrot** (baseline no) | | |  |  |  |  |  |  |  |
| Yes | 1.19  (0.85-1.68) | 1.30  (0.81-2.08) | **2.63**  **(1.45-4.75)** | 1.28  (0.94-1.75) | **1.61**  **(1.04-2.49)** | **2.86**  **(1.68-4.85)** | **1.51**  **(1.05-2.18)** | **2.40**  **(1.52-3.79)** | **2.81**  **(1.51-5.22)** |
| **Vaccination of sheep with severe footrot** (baseline no) | | | |  |  |  |  |  |  |
| Yes | **3.32**  **(1.03-10.67)** | 1.89  (0.39-9.18) | 3.65  (0.59-22.71) | **4.46**  **(1.40-14.20)** | 3.40  (0.80-14.48) | **7.00**  **(1.40-35.07)** | x | x | x |
| **Vaccination of ewes** (baseline no) | | |  |  |  |  |  |  |  |
| Yes | x | x | x | x | x | x | **0.62**  **(0.41-0.94)** | **0.39**  **(0.21-0.71)** | 0.65  (0.29-1.45) |
| **Routine foot trim the flock** (baseline no) | | |  |  |  |  |  |  |  |
| Trim no bleeding | 1.04  (0.53-2.02) | 0.24  (0.05-1.08) | 2.91  (0.80-10.57) | 0.90  (0.49-1.65) | 0.44  (0.15-1.32) | 2.04  (0.66-6.27) | 1.28  (0.68-2.42) | 1.46  (0.59-3.63) | **4.11**  **(1.15-14.65)** |
| Bleeding | 1.39  (0.99-1.94) | 0.79  (0.50-1.27) | **4.16**  **(2.03-8.53)** | **1.38**  **(1.01-1.87)** | 0.95  (0.62-1.47) | **3.25**  **(1.77-5.95)** | **1.71**  **(1.22-2.40)** | **2.42**  **(1.56-3.76)** | **5.53**  **(2.80-10.93)** |
| **Locomotion score farmer recognised sheep as lame** (baseline score 1) | | | | |  |  |  |  |  |
| 2 | 1.30  (0.92-1.83) | 1.58  (0.98-2.57) | 1.78  (0.94-3.37) | 1.33  (0.97-1.83) | 1.38  (0.88-2.17) | **1.81**  **(1.02-3.22)** | x | x | x |
| 3 | 1.27  (0.72-2.22) | 1.18  (0.51-2.75) | **2.83**  **(1.17-6.82)** | 1.08  (0.66-1.78) | 0.93  (0.43-2.01) | 1.98  (0.87-4.49) | x | x | x |
| 4 or more | 1.12  (0.15-7.77) | **7.37**  **(1.23-44.30)** | **10.61**  **(1.47-76.28)** | 1.31  (0.26-6.68) | **6.14**  **(1.36-27.69)** | **7.14**  **(1.28-39.85)** | x | x | x |
| **Number of times sheep lame before culling** (baseline no culling for lameness) | | | | |  |  |  |  |  |
| 1 | 0.60  (0.25-1.43) | 0.48  (0.01-2.35) | 0.00  (0.00-4.18e+109) | 0.62  (0.27-1.42) | 0.59  (0.16-2.14) | **0.00**  **(0.00-0.00)** | **0.37**  **(0.15-0.91)** | 0.30  (0.08-1.11) | 0.38  (0.05-3.19) |
| 1-<2 | 0.87  (0.53-1.43) | 1.41  (0.70-2.82) | 0.87  (0.34-2.21) | 0.75  (0.47-1.18) | 1.10  (0.57-2.10) | 0.81  (0.34-1.89) | 1.03  (0.64-1.64) | 0.71  (0.37-1.36) | 0.68  (0.26-1.75) |
| >2 | 1.39  (0.94-2.05) | **2.12**  **(1.25-3.58)** | 1.52  (0.78-3.04) | 1.21  (0.85-1.73) | **1.63**  **(1.00-2.66)** | 1.39  (0.76-2.56) | **1.82**  **(1.24-2.72)** | 1.25  (0.75-2.09) | 1.65  (0.83-3.30) |
| Persistently lame | **2.10**  **(1.04-4.21)** | 1.24  (0.41-3.74) | 1.40  (0.40-4.84) | **2.22**  **(1.14-4.33)** | 1.31  (0.45-3.85) | 1.80  (0.58-5.58) | **2.29**  **(1.06-4.95)** | 1.45  (0.55-3.82) | 1.70  (0.46-6.36) |
| **Isolation of new sheep on arrival** (baseline did not isolate) | | | |  |  |  |  |  |  |
| Sometimes | 0.59  (0.29-1.20) | 0.46  (0.16-1.33) | 0.50  (0.16-1.60) | 0.83  (0.43-1.58) | 0.52  (0.20-1.35) | 0.38  (0.12-1.17) | 0.49  (0.23-1.03) | 0.68  (0.29-1.63) | **0.18**  **(0.05-0.72)** |
| Usually | 0.72  (0.38-1.35) | 0.66  (0.28-1.55) | 0.35  (0.12-1.04) | 0.79  (0.44-1.40) | 0.70  (0.32-1.53) | 0.44  (0.17-1.16) | 0.54  (0.28-1.03) | 0.85  (0.39-1.85) | 0.35  (0.12-1.06) |
| Always | **0.51**  **(0.30-0.87)** | 0.52  (0.25-1.06) | 0.45  (0.19-1.06) | **0.58**  **(0.36-0.95)** | 0.53  (0.27-1.03) | **0.46**  **(0.21-0.99)** | **0.47**  **(0.27-0.82)** | **0.34**  **(0.17-0.67)** | **0.38**  **(0.16-0.90)** |
| No new arrivals | **0.53**  **(0.29-0.97)** | 0.71  (0.31-1.64) | **0.29**  **(0.09-0.88)** | **0.49**  **(0.28-0.84)** | 0.62  (0.30-1.31) | **0.25**  **(0.09-0.69)** | **0.55**  **(0.30-1.00)** | **0.46**  **(0.21-0.98)** | **0.26**  **(0.09-0.75)** |
| **Home bred replacement ewes** (baseline no) | | |  |  |  |  |  |  |  |
| Yes | 0.78  (0.55-1.12) | **0.55**  **(0.33-0.89)** | 0.82  (0.44-1.52) | x | x | x |  |  |  |
| **Time to treatment (**baseline first day seen lame) | | | |  |  |  |  |  |  |
| <3 days | x | x | x | x | x | x | 1.88  (0.95-3.73) | 0.94  (0.38-2.35) | 3.68  (0.45-30.04) |
| <7 days | x | x | x | x | x | x | **2.48**  **(1.22-5.03)** | 2.13  (0.85-5.33) | **9.44**  **(1.15-77.58)** |
| >7 days | x | x | x | x | x | x | **2.81**  **(1.19-6.59)** | 1.63  (0.54-4.95) | **11.10**  **(1.20-102.86)** |
| Did not treat any lame sheep | x | x | x | x | x | x | **0.00**  **(0.00-0.00**) | **0.00**  **(0.00-0.00)** | **0.74**  **(0.74-0.74)** |
| **Number of sheep treated at locomotion score farmer recognised sheep lame** (baseline 1 sheep) | | | | | | | |  |  |
| 2-5 | x | x | x | x | x | x | 1.19  (0.76-1.86) | **2.78**  **(1.26-6.10)** | 0.46  (0.18-1.18) |
| 6-10 | x | x | x | x | x | x | 1.52  (0.86-2.67) | **3.85**  **(1.59-9.29)** | 2.34  (0.89-6.15) |
| >10 | x | x | x | x | x | x | **1.88**  **(1.00-3.51)** | **5.99**  **(2.36-15.2)** | **2.96**  **(1.05-8.38)** |
| Did not treat individuals | x | x | x | x | x | x | 2.24  (0.20-28.8) | 8.69  (0.56-134) | **0.00**  **(0.00-0.00)** |

OR: odds ratio, 95% CI: 95% confidence interval (lower, upper), x: management practice not included in model, , Odds ratio significantly (p≤0.05) different from the reference category are highlighted in bold (Wald’s test). Number and percentage of flocks performing each management practice are found in Supplementary Tables 7a, 7b and 8.
